# Structure and spectroscopy of methionyl-methionine for aquaculture

**DOI:** 10.1038/s41598-020-80385-z

**Published:** 2021-01-11

**Authors:** Stewart F. Parker, Nicholas P. Funnell, Kenneth Shankland, Elena A. Kabova, Thomas Häußner, Hans-Joachim Hasselbach, Sascha Braune, Christoph Kobler, Peter W. Albers

**Affiliations:** 1 ISIS Facility, STFC Rutherford Appleton Laboratory, Chilton, Didcot, OX11 0QX UK; 2 School of Pharmacy, University of Reading, Reading, RG6 6AD UK; 3 Evonik Nutrition and Care GmbH, Rodenbacher Chaussee 4, 63457 Hanau-Wolfgang, Germany; 4 Evonik Operations GmbH, Rodenbacher Chaussee 4, 63457 Hanau-Wolfgang, Germany

**Keywords:** Chemistry, Physical chemistry

## Abstract

The amino acid l-methionine is an essential amino acid and is commonly used as a feed supplement in terrestrial animals. It is less suitable for marine organisms because it is readily excreted. It is also highly water soluble and this results in loss of the feed and eutrophication of the water. To address these problems, the dipeptide dl-methionyl-dl-methionine (trade name: AQUAVI Met-Met) has been developed as a dedicated methionine source for aquaculture. The commercial product is a mixture of a racemic crystal form of d-methionyl-d-methionine/l-methionyl-l-methionine and a racemic crystal form of d-methionyl-l-methionine/l-methionyl-d-methionine. In this work, we have computationally, structurally, spectroscopically and by electron microscopy characterised these materials. The microscopy and spectroscopy demonstrate that there is no interaction between the dd–ll and dl–ld racemates on any length scale from the macroscopic to the nanoscale.

## Introduction

**Figure 1:**
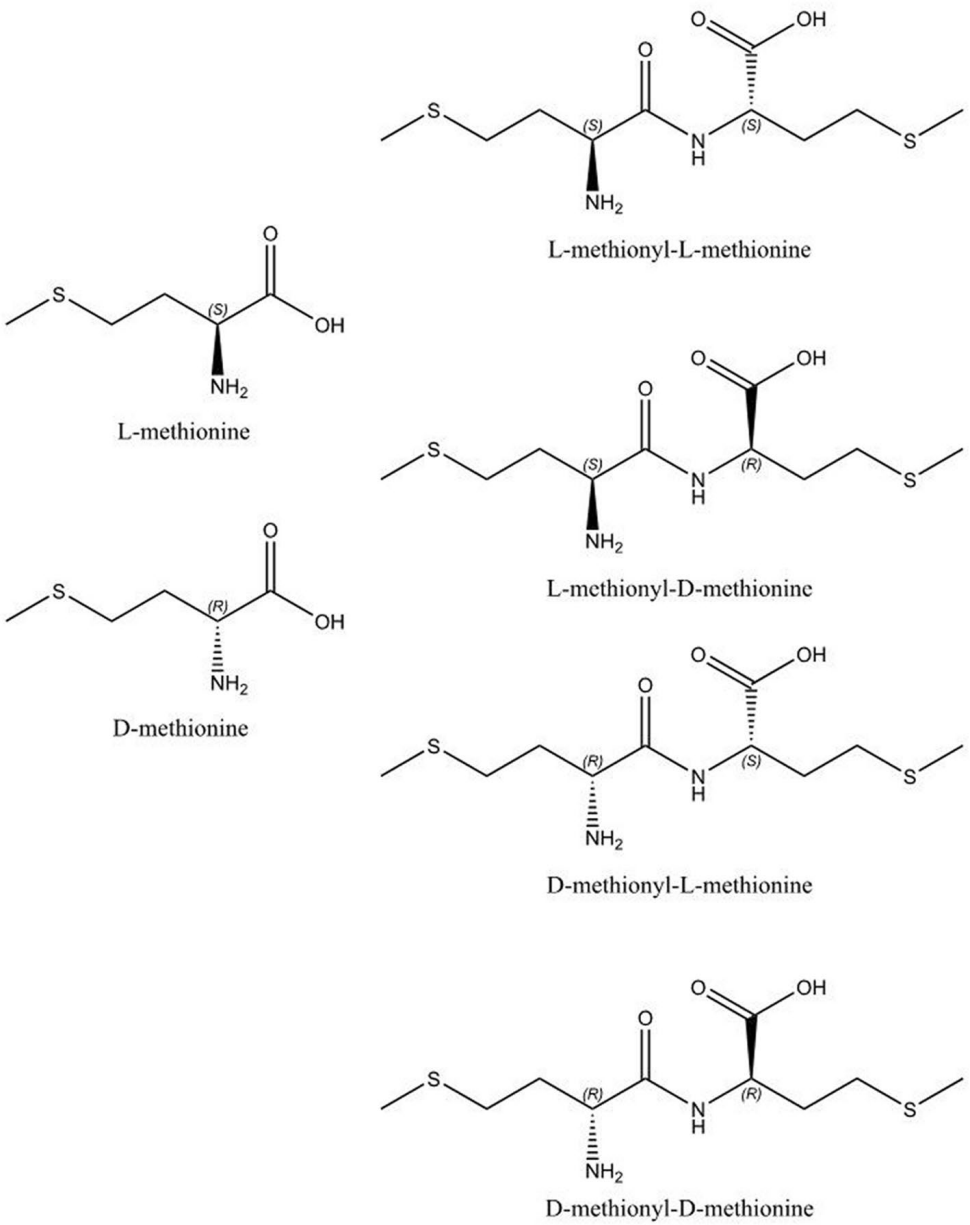
The structures of the two enantiomers of methionine, and the four stereoisomers of methionyl-methionine.

The amino acid l-methionine ((2*S*)‐2‐amino‐4‐(methylsulfanyl)butanoic acid, Fig. 1) is an essential amino acid i.e. it cannot be synthesised in vivo^1^. The industrial synthesis of methionine from hydrogen cyanide, methanethiol and acrolein^2^ results in the racemate, whilst pure l-methionine can be obtained by fermentation^3^. However, dl-methionine is a valuable food additive in animal nutrition^4^, as the “unnatural” d-methionine diastereoisomer can be converted to the desired l-form physiologically^5^.

In contrast to its use as a feed supplement in terrestrial animals, methionine is less suitable for marine organisms. This is because a significant amount of the added methionine is excreted via the gills or kidneys or used as an energy source, rather than promoting weight gain^6^. In addition, methionine is highly water soluble (50–55 g L^−1^) and this results in loss of the feed and eutrophication of the water. To address these problems, the dipeptide dl-methionyl-dl-methionine (trade name: AQUAVI Met-Met, CAS-No.: 52715-93-2) has been developed^7,8^ as a dedicated methionine source for aquaculture e.g. for bottom feeders such as shrimp, prawn and other crustaceans^9–11^. This compound has the property that it is cleaved enzymatically by fish and crustaceans under physiological conditions to provide free d- and l-methionine. Due to its low water solubility and high hydrothermal stability during feed processing, the risk of leaching is minimized in spite of the small particle sizes (90% < 300 μm) used for animal feed, which provide a fine distribution in feed pellets and extrudates, and slow release in the animal’s gut with enhanced nutritional value.

Whilst methionine itself has been studied both structurally^12–14^ and spectroscopically^15–21^, its dimer methionyl-methionine has been much less studied. Methionine contains a single asymmetric carbon, and thus exists as l and d enantiomers, whilst there are four possible stereoisomers (diastereoisomers) of methionyl-methionine: ll, ld, dl and dd, see Fig. 1. The six possible crystalline forms of methionyl-methionine are listed in Table 1 below. Diastereoisomers can have very different physicochemical properties and those of methionyl-methionine have markedly different solubilities (dd–ll: 21 g L^−1^, dl–ld: 0.4 g L^−1^)^7,8^, which provides the basis of a mechanism for the timed release of the nutrient.

**Table 1:** The six possible crystalline forms of methionyl-methionine.

Crystalline form	Status	Abbreviation used in this work
l-methionyl-l-methionine only	Single stereoisomer; reported	ll
l-methionyl-d-methionine only	Single stereoisomer; not reported	ld
d-methionyl-l-methionine only	Single stereoisomer; not reported	dl
d-methionyl-d-methionine only	Single stereoisomer; not reported	dd
d-methionyl-d-methionine/l-methionyl-l-methionine	Racemic crystal of dd and ll stereoisomers; this work	dd–ll
d-methionyl-l-methionine/l-methionyl-d-methionine	Racemic crystal of dl and ld stereoisomers; this work	dl–ld

Of the six possible forms of methionyl-methionine, only the ll form has been structurally determined^22^. The only spectroscopic work on this form was a Raman study in aqueous solution and adsorbed on a silver colloid^23^. In the present work, we have structurally characterised the commercially important d-methionyl-d-methionine/l-methionyl-l-methionine and d-methionyl-l-methionine/l-methionyl-d-methionine, and report complete assignments of their solid-state spectra. To this end, we have used a combination of inelastic neutron scattering (INS), infrared and Raman spectroscopies, with the assignments provided by periodic density functional theory (DFT) calculations of the vibrational spectra.

## Results and discussion

### Electron microscopy

**Figure 2:**
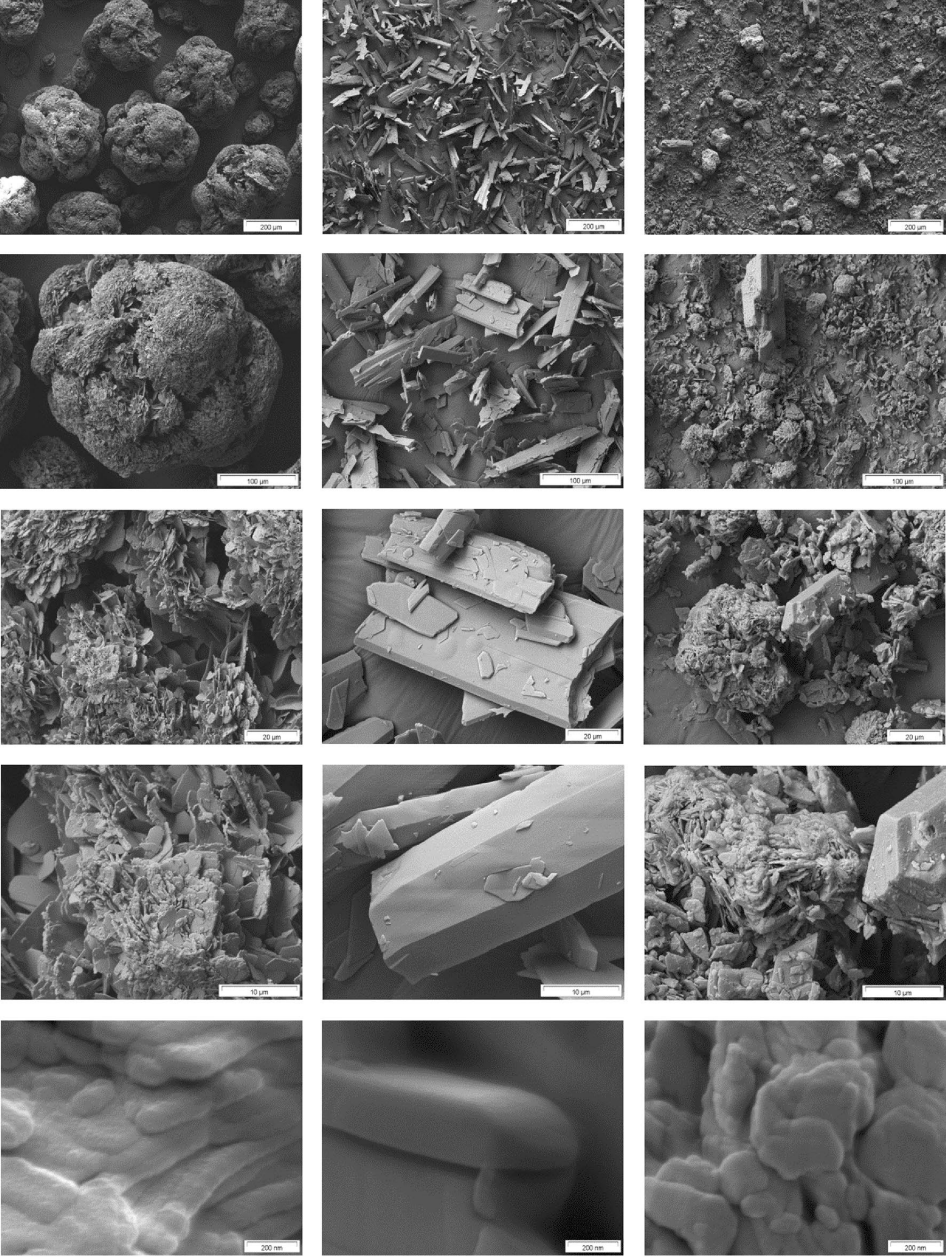
SEM images with increasing magnification of the samples “as received”, dusted onto adhesive carbon tape, show the morphology from the macroscale down to submicron dimensions. From left to right dl–ld isolated/separated dl–ld fraction; dd–ll isolated/separated dd–ll fraction; mixture (37% dd/ll + 63% dl/ld). From top to bottom the scale bars are: 200, 100, 20, 10 μm and 200 nm.

The SEM images, Fig. 2, illustrate the different crystallite size, shape and stacking properties of the primary crystallites in the aggregates and agglomerates. For the dl–ld sample at low magnification, granular spheres consisting of an arrangement of well-adherent, coherently stacked and tilted platelets of fine crystallinity are observed. For dd–ll, comparatively larger, compact linear crystals and plates are formed. Thus there are distinct differences in the morphology of this pairs of diastereoisomers.

Crystallization of a solution consisting of 55% dl–ld and 45% dd-ll, results in a solid product with 63% dl–ld and 37% dd–ll. From the last column of Fig. 2, it can be seen that both different crystal forms are present in the mixture, but at smaller size due to mixing/dispersing treatment. The size difference is particularly apparent in the top right image, where the largest particles are only ~ 50 μm, whereas for the pure materials (top left and centre images) many of the particles exceed 200 μm in size. Only at higher magnification (e.g. 20 μm resolution, middle row of images, rightmost image) are the characteristic spheres and plates of the dl–ld and dd–ll components respectively, clearly seen. As the SEM images of the mixture of dl–ld and dd–ll can be interpreted as being the sum of the components, we conclude that dl–ld and dd–ll crystallize independently and do not form mixed crystals at the macroscale.

**Figure 3:**
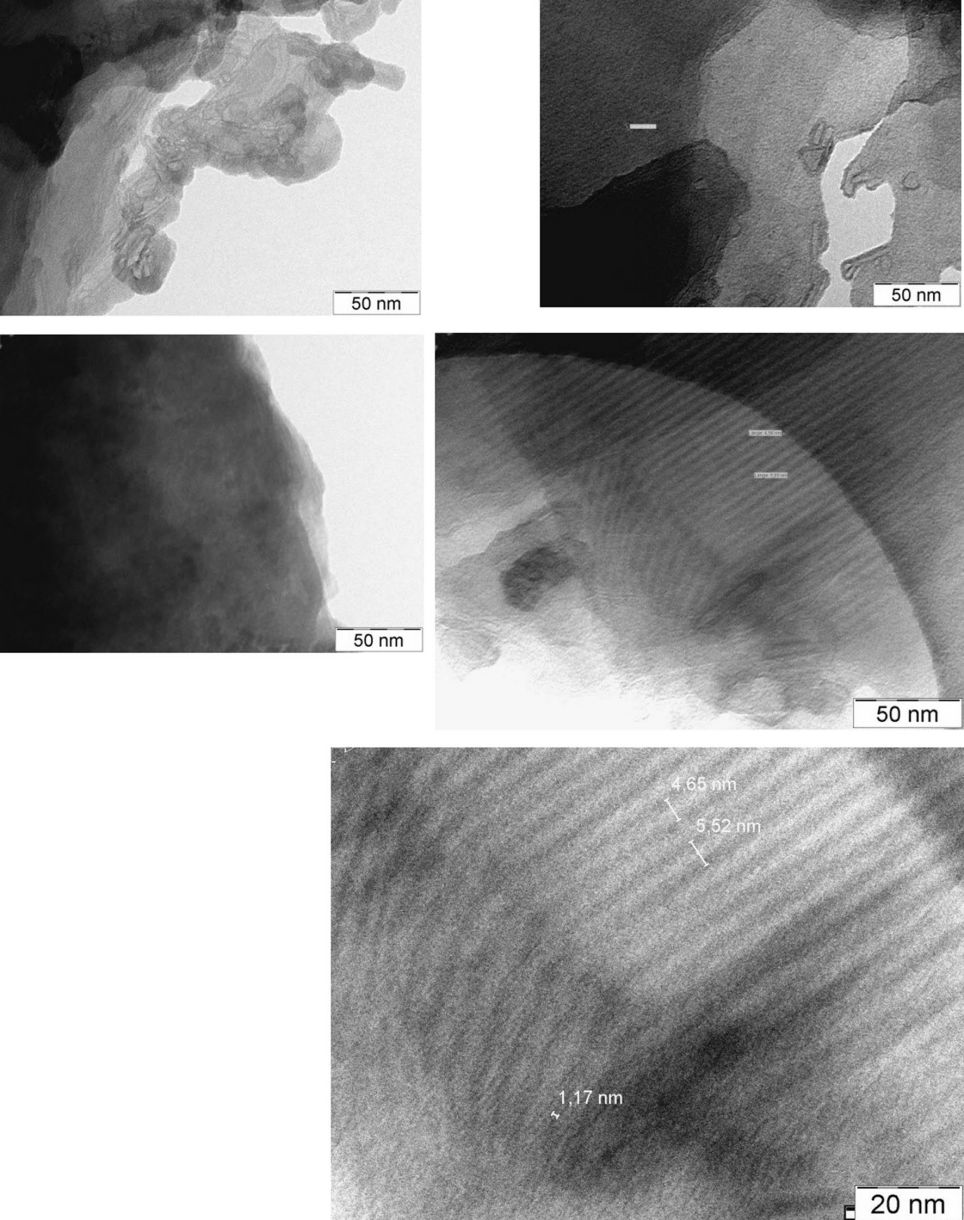
TEM images of: dl–ld (left) and dd–ll (right).

Complementary to the macroscopic differences in the crystal morphologies of the stereoisomers, samples dl–ld and dd–ll, TEM at higher magnification, Fig. 3, suggests also some differences in the internal crystal sizes and degree of long-range ordering. It can be seen that the dl–ld form (left column of Fig. 3) consists of aggregates of very small crystallites (~ 10 nm), whereas sample tilt experiments on Moire-contrast and lattice fringes suggests a more extended ordering inside of the much larger (10–100 μm) crystals of dd–ll (right column of Fig. 3).

### X-ray photoelectron spectroscopy (XPS)

Comparison of the shape and position of the photoelectron signals (Supplementary Figure S1) as recorded with monochromatic AlKα radiation with a spot size of 1 mm^2^, illustrates that even for the topmost atomic layers no significant chemical differences between the samples are present. Mostly alkyl functions (ca. 284–285 eV), peptide-C=O and COO^−^ (286–288 eV), amino N–H (ca. 398–399 eV) and NH_3_^+^ nitrogen (ca. 400–401 eV) and C=O oxygen (ca. 530–531 eV) are detected. There is no evidence for C–OH groups or traces of adsorbed moisture/water. For sulfur, the S2p-doublet is indicated by the line asymmetry, the signal is dominated by the feature around 163 eV (sulfane-S); only traces of minor surface oxidation of traces of S are suggested by weak features at ca. 166 eV. In conclusion, the presence of surface oxidation or traces of crystallisation aids or defoamers in the topmost atomic layers can be ruled out. Thus the XPS data demonstrates that there are no chemical differences present at the surface of the two pairs of diastereoisomers, dd–ll or dl–ld, and this can be eliminated as a possible reason for the difference in solubility of the materials.

### X-ray crystallography

Table 2 below provides a summary comparison of the two crystal structures determined in this work, and the previously known structure of l-methionyl-l-methionine. Full crystal data and structure refinement details for dd–ll and dl–ld are given in Supplementary Table S1.

**Table 2:** A comparison of some key parameters for the three crystal structures of methionyl-methionine. RMSD = root mean square deviation for a 15 molecule overlay of the experimental and optimized (DFT-D, variable cell) crystal structures in Mercury.

	ll	dd–ll	dl–ld
Structure			
Sp. group	*P*2_1_2_1_2_1_	*P*2_1_/*c*	*P*2_1_/*c*
Z, Z′	4, 1	4, 1	4, 1
Volume (Å^3^)	1347.89	1339.45	1334.11
T (K)	298	150	293
ρ (g cm^−3^)	1.382	1.390	1.396
RMSD (Å)	0.048	0.026	0.069

**Figure 4:**
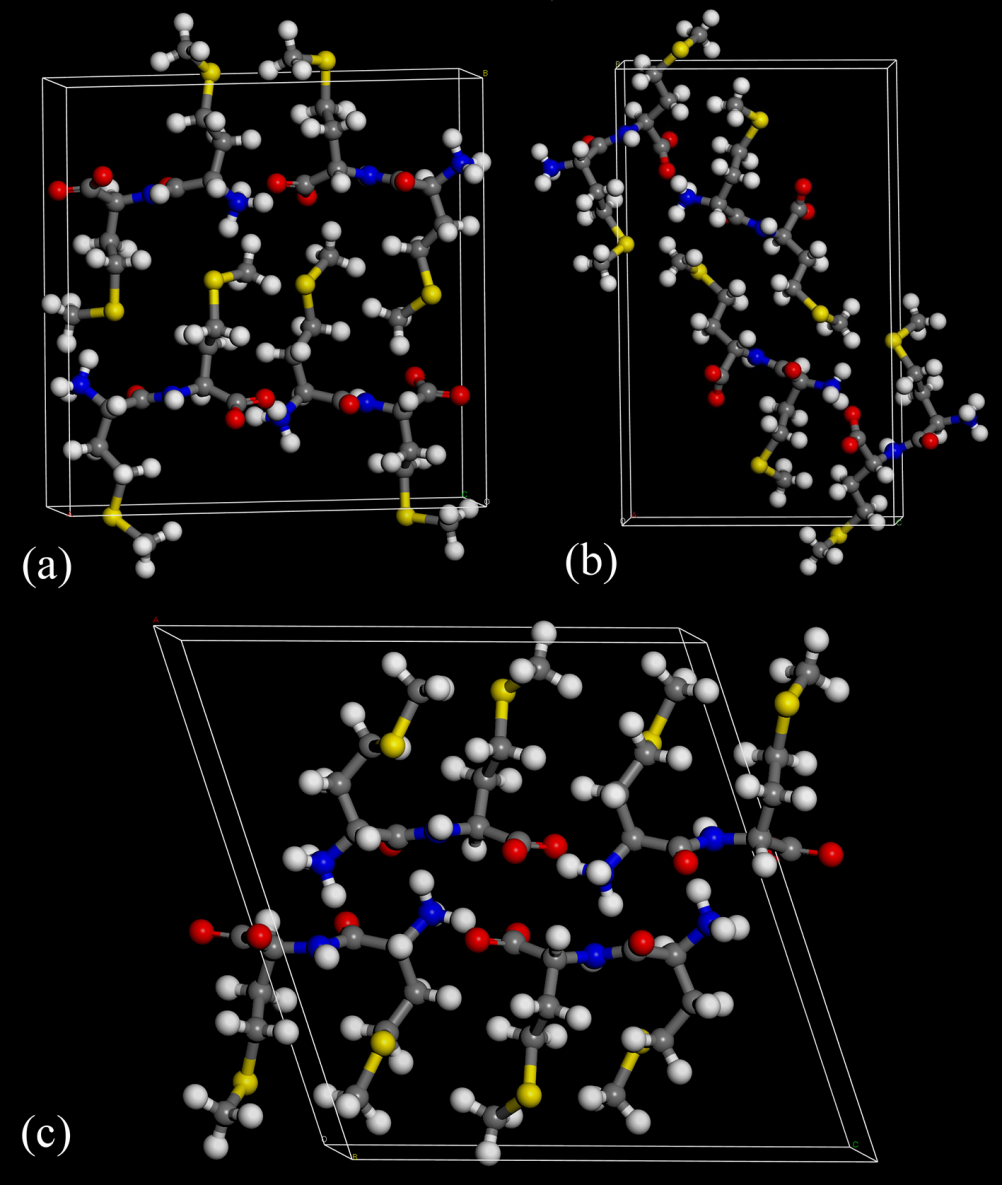
Crystal structures of: (**a**) ll, (**b**) dd–ll and (**c**) dl–ld. (Key: grey = C, white = H, red = O, blue = N, yellow = S). ORTEP plots of the asymmetric units of the novel dd–ll and dl–ld structures reported here are shown in Supplementary Figures S8 and S9.

**Figure 5:**
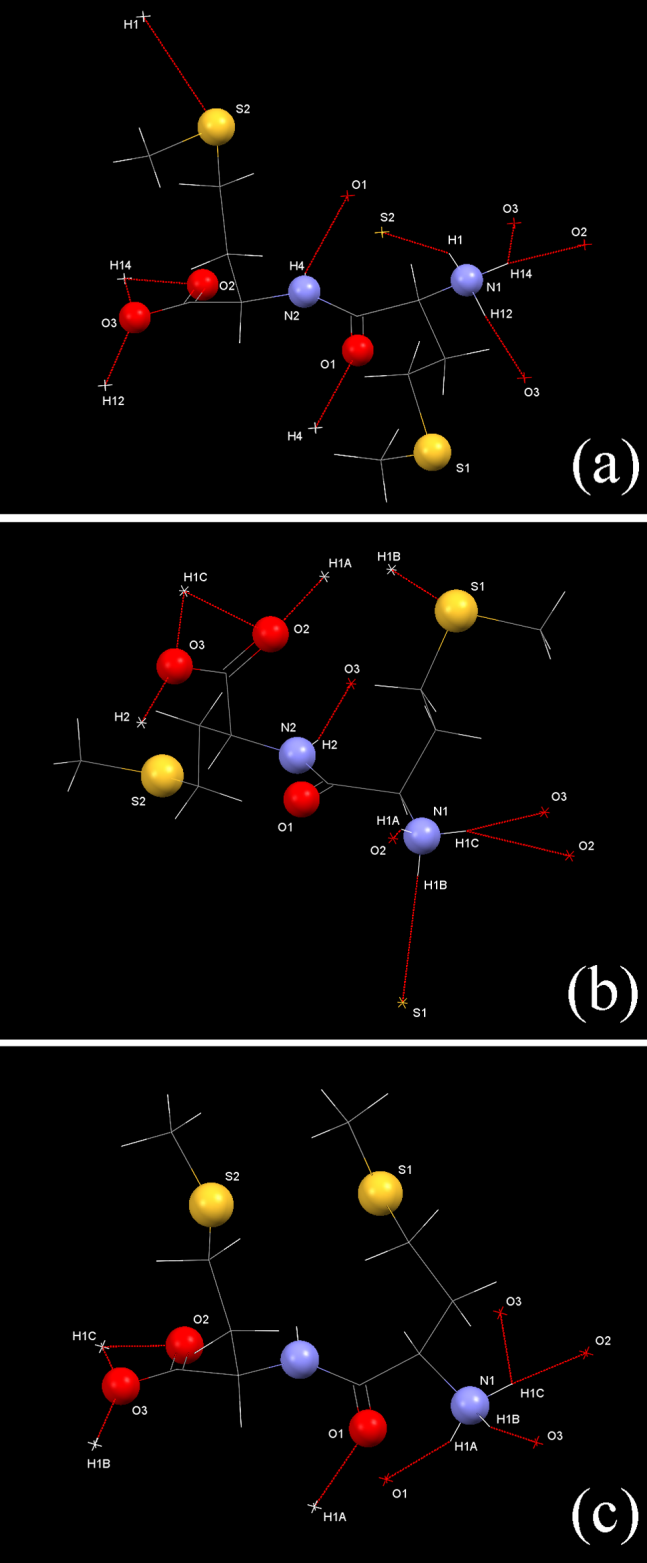
Hydrogen bonding (red dotted lines) around individual molecules in the crystal structures of: (**a**) ll, (**b**) dd–ll and (**c**) dl–ld methyl-methionine.

Figure 4 shows the crystal structures of: ll^22^, dd–ll and dl–ld, whilst Fig. 5 shows the hydrogen bonding around the individual molecules each of the asymmetric units. In all cases, the molecules are in the zwitterionic form, i.e. with –NH_3_^+^ and –CO_2_^−^ functionalities. The conformations of the ions are markedly different in the three structures, the only common feature being the planar peptide linkage. In particular, the dl–ld structure adopts a hairpin-like conformation (S–S distance of 4.86 Å) compared to the extended conformations seen in both ll (S–S distance of 9.75 Å) and dd–ll (S–S distance of 9.06 Å). Crystal packing similarity calculations performed in Mercury confirm that there is no significant structural similarity between any of the three crystal structures.

Hydrogen bonding plays a significant role in all the structures. As noted previously^22^, the major interaction in ll is an asymmetric bifurcated H-bond from H14 of the –NH_3_^+^ to both O2 and O3 of the carboxylate, with bond lengths of 1.758 and 2.307 Å respectively. O3 also participates in a second H-bond (1.679 Å) to H12 of the –NH_3_^+^ of an adjacent molecule. There is also a much longer (2.577 Å) N–H‧‧‧O bond, linking the peptide units in adjacent cells to form chains running parallel to the *c*-axis. Previously unrecognised^22^, there is a long (2.785 Å) N–H‧‧‧S contact from H1 of the –NH_3_^+^ group.

The dd–ll structure also has 5 hydrogen bonds. An asymmetric bifurcated H-bond is again evident, this time from H1C of the –NH_3_^+^ to both O2 and O3 of the carboxylate, with bond lengths of 1.829 and 2.568 Å respectively. O2 and O3 both participate in additional H-bonds to H2 of the amide (2.065 Å) and H1A of the –NH_3_^+^ (2.042 Å) respectively. Again, there is a long (2.799 Å) N–H‧‧‧S contact originating from H1B of –NH_3_^+^ group. Interestingly, though not exceptionally, the carbonyl of the amide group is not involved in any hydrogen bonding.

In dl–ld, there are only 4 hydrogen bonds, with some similarities to the bonding pattern seen in the ll-only structure. The asymmetric bifurcated H-bond is again in evidence from H1C of the –NH_3_^+^ to both O2 and O3 of the carboxylate, with bond lengths of 1.713 and 2.293 Å respectively. O3 again participates in a second H-bond (1.737 Å) to H1B of the –NH_3_^+^ of an adjacent molecule, with the H1C‧‧‧O3‧‧‧H1B pattern linking the molecules in chains parallel to the *b*-axis. The final interaction (1.912 Å) is between O1 of the amide and H1A of the –NH_3_^+^. In this structure, it is the N–H of the amide that does not participate in hydrogen bonding and, unlike the previous two structures, there is no evidence of an N–H‧‧‧S interaction.

A more complete description of the hydrogen bonding in each crystal structure (in the form of hydrogen bonding coordination likelihood calculations and graph set analysis^24–27^) is given in Supplementary Tables S2–S4 and S6–S8. The graph set analysis provides a comprehensive standalone categorisation of hydrogen bonding patterns in the three structures. The hydrogen bonding likelihoods provide a complementary perspective, setting the observed unique hydrogen bonds in each structure against a background of expected hydrogen bond probabilities, based on the 1,000,000 + crystal structures in the CSD. Note that a comparison of the three crystal structures, using the *Crystal Packing Similarity* feature of Mercury, revealed no one-dimensional (column) or two-dimensional (layer) correspondences between the structures. Note also that none of these crystal packing analyses can directly explain the observed solubility differences between the three structures.

### Vibrational spectroscopy

**Figure 6:**
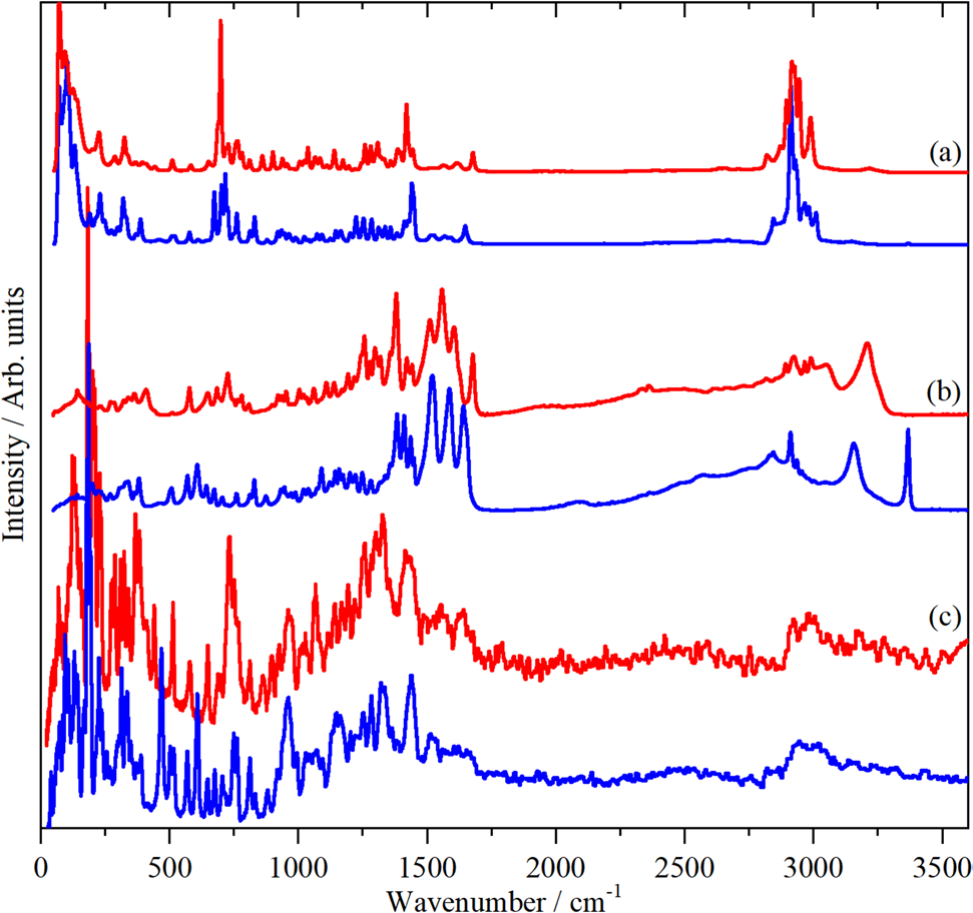
Vibrational spectra of dd-ll- (red) and dl-ld-methionyl-methionine (blue). (**a**) Raman, (**b**) infrared and (**c**) INS spectra.

Figure 6 shows the Raman, infrared and INS spectra of the dd–ll and dl–ld racemates, respectively. The spectra of the two compounds are clearly different and are readily distinguishable. The complementarity of the three forms of vibrational spectra is immediately apparent. The Raman spectra are dominated by the C–H stretch modes (2850–3000 cm^−1^), the C–S stretch modes (~ 700 cm^−1^) and the lattice modes (< 200 cm^−1^). The infrared spectra are dominated by the N–H stretch modes and the associated effects of hydrogen bonding (2000–3300 cm^−1^) and the complex manifold of the carboxylate C–O stretch, the –NH_3_^+^ bending modes and the mixed C=O stretch and in-plane N–H bend modes (amide I and II, 1500–1700 cm^−1^). In the INS spectra, the strongest features are the large amplitude low-energy modes (< 500 cm^−1^), particularly the methyl torsions at ~ 250 cm^−1^. The most striking difference between the two compounds is the strong band at 3370 cm^−1^ in the infrared spectrum of the dl–ld form that is not present in that of the dd–ll form. This is assigned as the N–H stretch mode of the peptide linkage. As noted earlier, in the dd–ll form it is involved in a relatively strong H-bond (1.819 Å), whereas it is only weakly bonded (2.742 Å) in the dl–ld form, thus it approaches the 3440–3460 cm^−1^ range found for unassociated amides^28^.

**Figure 7:**
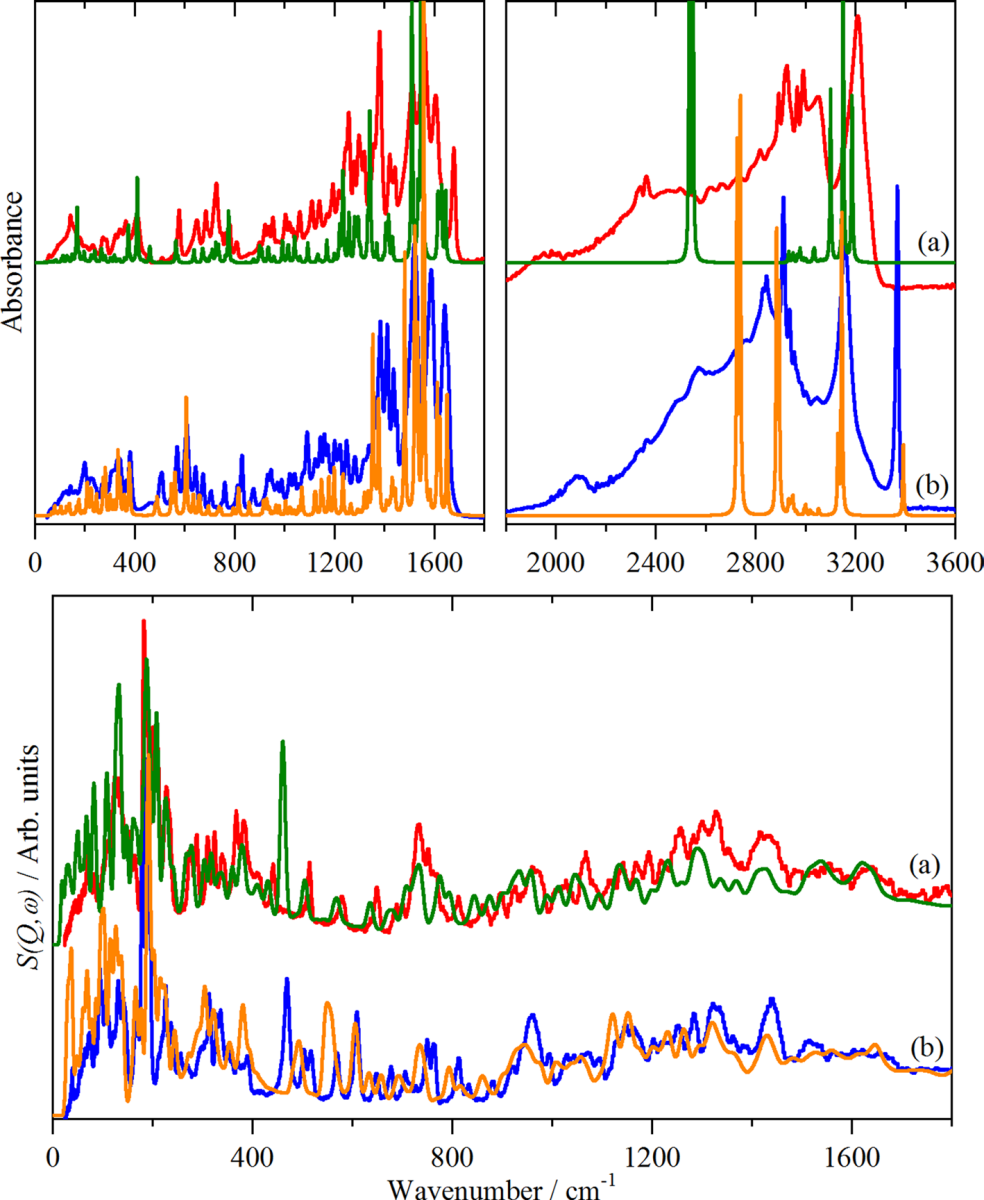
Comparison of observed and calculated spectra of methionyl-methionine: (**a**) the dd–ll racemate (red: experimental, olive: calculated) and (**b**) the dl–ld racemate (blue: experimental, orange: calculated). Upper panel: infrared spectra, lower panel: INS spectra. The experimental infrared spectra in the 1800–3600 cm^−1^ region are × 2 ordinate expanded with respect to the 0–1800 cm^−1^ region, the calculated infrared spectra are × 0.5 ordinate expanded.

Overall, the spectra are typical of amino acids in the zwitterionic form, but with the added complication of the peptide linkage^28^. In order to go beyond simple group frequency assignments, we have used periodic density functional theory to provide more definitive assignments. Comparisons of the experimental and calculated infrared and INS spectra for the dd–ll and dl–ld racemates are shown in Fig. 7.

From Fig. 7 (lower panel), it can be seen that the calculated INS spectra are in good agreement with the observed spectra. The overall agreement is typical of that obtained with periodic-DFT^29,30^. While scaling of the *x*-axis of the calculated spectra would improve the agreement, we have chosen not to use this somewhat empirical approach, allowing a realistic assessment of the quality of the calculation. There are two areas where there are obvious differences in the spectra: the modes below 150 cm^−1^, and those around 400 cm^−1^. The low energy modes are where the whole body motions (translations and librations) occur and, typically, these show significant vibrational dispersion (variation of transition energy with wavevector). Unlike optical spectra, INS spectra are sensitive to all wavevectors, (*k*, Å^−1^) across the entire Brillouin zone; however, the calculations are only for the Γ-point, *k* = 0, so the complexity in the low energy region is not included. The modes around 400 cm^−1^ will be discussed later.

The calculated infrared spectra, Fig. 7 (upper panel), are in generally good agreement with the experimental data for the transition energies, however, the intensities above 1300 cm^−1^ are poorly reproduced and particularly, the mountain of intensity between 2000 and 3400 cm^−1^ is not accounted for in the calculations. The former are the modes that are involved in the hydrogen bonding and this is not well described with the PBE functional^31^. Similar effects have been seen for other systems with hydrogen bonding^32,33^. The latter is a consequence of the electrical anharmonicity coupled with the presence of overtone and combination modes that are in Fermi resonance with the –NH_3_^+^ stretching modes. Both of these effects are outside of the harmonic approximation, so are not included in the calculated spectra. There is also a general pattern, in that, unlike the INS spectra, the infrared peak widths do not change in a predictable manner and this variation is not accounted for in the calculated spectra.

Supplementary Table S5 lists the observed transition energies for both the dd–ll and the dl–ld racemates and the complete list of calculated transition energies and their infrared intensities are given in Tables S9 and S10. As shown in Table 2, both systems crystallise in the monoclinic, centrosymmetric space group, *P*2_1_/*c* with four zwitterions in the primitive cell. This results in four factor group components; however, as is apparent from the spectra and confirmed by the calculations, the factor group splitting is small (less than 10 cm^−1^) for the modes above 400 cm^−1^. At lower energies the spread is larger, this probably a consequence of the lack of symmetry (all the ions are on *C*_1_ sites) which results in extensive mixing between the modes and also with the external (translations and librations) modes. This renders simple description of the modes meaningless, so only for the most obvious cases is there a description for the modes below 200 cm^−1^.

**Figure 8:**
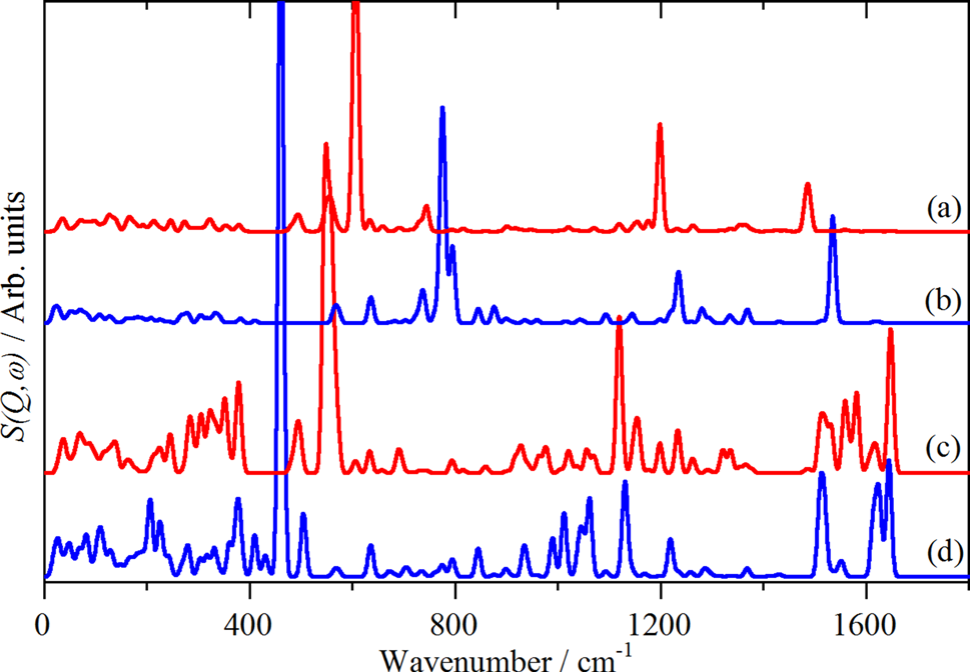
Pseudo-spectra of: (**a**) the peptide amide hydrogen in the dl–ld racemate and (**b**) in the dd–ll racemate, (**c**) the hydrogens in the –NH_3_^+^ moiety in the dl–ld racemate and (**d**) in the dd–ll racemate.

An alternative approach is to isolate the modes that involve significant displacement of a particular atom or moiety to generate “pseudo-spectra”. Figure 8 shows this for the hydrogen of the peptide N–H and the hydrogens of the –NH_3_^+^ for both racemates. It can be seen that there are significant differences between the two materials. For the peptide amide hydrogen, the in-plane N–H bend (amide II) is at 1486 (1520 observed) and 1534 (1558 observed) cm^−1^, for the dd–ll and dl–ld forms respectively. The amide III (OC–NH stretch) is calculated at 1198 (dd–ll) and 1235 (dl–ld) cm^−1^ (1222 and 1256 cm^−1^ observed, both weak in all forms of spectroscopy), the intensity of this mode in the N–H only “spectrum” demonstrates that it is strongly coupled to the amide II mode. The same pattern of the modes occurring at higher energy for the dd–ll than the dl–ld form is repeated for the out-of-plane N–H bend. This is consistent with the dd–ll form having stronger hydrogen bonds involving the peptide N–H, as noted earlier. (For H-bonded systems, the X–H stretch mode transition energy decreases as the hydrogen bond strength increases, while for the bending modes the transition energy increases with increasing hydrogen bond strength^34,35^).

For the –NH_3_^+^ modes the only major difference is the transition energy of the torsional mode: 548 (469 observed, dd–ll) and 461 (368 observed, dl–ld) cm^−1^. This also explains the difference in the 400 cm^−1^ region noted previously in Fig. 7. A mismatch between observed and calculated –NH_3_^+^ torsional energies is commonly found, e.g. for aliphatic amines^36^ and aromatic hydrochlorides^32^.

Pseudo-spectra for other atoms of interest: the methyl groups, the carbonyl and carboxylate oxygen atoms and the sulfur atoms are shown in Supplementary Figs. S2 and S3. In all cases the pseudo-spectra show that the entities are behaving as expected and the transition energies occur in the expected ranges^28,37^.

As noted in the introduction, the stereoisomers of methionyl-methionine have different solubilities (dd–ll: 21 g L^−1^, dl–ld: 0.4 g L^−1^)^7,8^. Thus it is essential to know if there is any interaction between the compounds, as this would affect the solubility. Supplementary Figs. S4–S6 show comparisons of the INS, infrared and Raman spectra of a physical mixture of the dd–ll and dl–ld racemates with synthetic spectra generated by scaled addition of the spectra of the individual components. It can be seen that the measured and synthetic spectra are identical. In all cases the relative proportions of the individual components was the same as that of the physical mixture.

## Conclusions

A full understanding of the physicochemical properties of various crystalline forms of a commercially important material such as Met-Met is critical to understanding its solid-state behaviour, both during processing and in use, and crystal structure determination is a key element of this understanding. Single-crystal X-ray structure determination remains the gold standard for such determinations, but it is not applicable in cases such as dl–ld, where a suitable single crystal could not be grown. Fortunately, crystal structure determination from powder diffraction data is a powerful alternative that in this case allowed us to complete the characterisation of Met-Met forms by solving the dl–ld crystal structure. Whilst modified direct-methods programs^38^ have been used to solve structures from a wide range of polycrystalline materials (e.g.^39,40^), it is global optimisation based approaches (as used in this work) that find particular utility with molecular organic materials such as dl–ld^41^. When solved from high-quality powder diffraction data, and particularly when subsequently verified with DFT-D3 calculations, a crystal structure obtained from a bulk powder is not markedly inferior to that of one obtained from a single crystal and provides an essential starting point for interpreting INS spectra. Recent developments both in electron diffraction^42^ and crystal structure prediction^43^ make it increasingly unlikely that the crystal structures of identified physical forms of important materials will remain undetermined, even when established X-ray crystallographic methods for single crystals and powders fail.

Additional structural insights can come from combining crystallographic results with other techniques. In this work we have computationally, crystallographically, spectroscopically and by electron microscopy characterised the commercial important dl–ld and dd-ll forms of methionyl-methionine. The electron microscopy (Figs. 2 and 3) showed that, at the macroscale, there was no evidence for any interaction between the dl–ld and dd–ll materials. This finding is supported by all three forms of vibrational spectroscopy that demonstrate that there is no interaction between the dd–ll and dl–ld racemates, as the spectra are simply sums of the component spectra. Thus the electron microscopy and the spectroscopy clearly show that the materials behave independently on all length scales from the macroscopic to the atomic.

There is one area where the dd–ll and dl–ld racemates are distinctly different. The SEM and TEM images (Figs. 2 and 3) show that the dl–ld is markedly less crystalline than the dd–ll form, (indeed, this was the reason that the structure of the dl–ld form had to be solved from powder diffraction data) and that they have very different morphologies. For applications in aquaculture, the key property of the materials is that they are much less water soluble than methionine. Compared with the dd–ll racemate, the dl–ld racemate is considerably less water soluble and has a DFT total energy that is lower by ca. 0.1 eV (9.6 kJ mol^−1^). DFT calculations of even higher accuracy (e.g. PBE0 + D3) are planned to confirm this energy difference and its possible significance in explaining the marked solubility difference between these two racemates.

## Materials and methods

### Materials

The samples were provided by Evonik Nutrition and Care GmbH, Business Line Animal Nutrition: dl–ld isolated/separated dl ld fraction, dd–ll isolated/separated dd ll fraction and a mixture (37% dd/ll packages + 63% dl/ld packages). They were prepared as described in^7,8^.

### X-ray crystallography

For the structure determination of the dd–ll form, a small crystal of dimensions ca. 0.03 × 0.07 × 0.07 mm was cut from one of the needle-like crystals obtained by slow recrystallization of methionyl-methionine from water/ethanol. Single-crystal X-ray diffraction data were collected on an Oxford Diffraction Gemini diffractometer, using Cu Kα incident radiation, with the crystal held at a temperature of 150 K using an Oxford Instruments Cryojet device. A redundancy constraint of 6 was specified for the collection, leading to a total data collection time of ca. 18 h. The crystal structure was solved^44^ and refined^45^ using the Olex2^46^ system.

The structure of dl–ld was determined from the powder. Powder X-ray diffraction (PXRD) was carried out using a Bruker D8 Advance diffractometer equipped with a LynxEye detector and monochromatic Cu Kα_1_ (λ = 1.54056 Å) radiation, operating in transmission capillary mode. A powdered sample of dl–ld methionyl-methionine was filled into a 0.7 mm borosilicate glass capillary, and data were collected over an angular range of 3°–70° 2θ with a step size of 0.017°. A variable-counting time scheme^47^ was used and the total data collection time was 18 h. Structure indexing and structure solution were performed using the *DASH* software package^48–50^. The starting molecular model used in the *DASH* global optimization procedure was derived from manipulation of the ll structure^22^ obtained directly from the Cambridge Structural Database (CSD Refcode METMET)^51^ The solved crystal structure was subjected to rigid-body Rietveld refinement ^52^ using *TOPAS*^53^. Periodic density functional theory with van der Waals dispersion corrections (DFT-D) was used for geometry optimization of the refined crystal structure. The Perdew–Burke–Ernzerhof functional was used with projector-augmented wave pseudopotentials and the Grimme D3 correction, as implemented in the *pw.x* executable of the *QuantumEspresso* program^54,55^. The lengths of bonds involving hydrogen atoms were normalized using *Mercury CSD*^56^, and input files for *pw.x* were then created from the normalized CIF using the *cif2qe* script of *QuantumEspresso*. Automatic *k*-point sampling was used; the kinetic energy cut-offs for plane waves and charge density were 50 and 400 Ry, respectively. The convergence thresholds on total energy and forces were set to 0.0001 and 0.001 a.u., respectively. Initial geometry optimization was carried out with lattice parameters fixed at their crystallographic values. All calculations were carried out on a Dell Precision T7810 workstation equipped with two 2.40 GHz 8-core Intel Xeon E5-2630 v3 CPUs, running the Microsoft Windows 10 operating system and using the Windows Subsystem for Linux feature to allow the Linux-compiled, MPI-enabled *pw.x* executable to utilize multiple cores. The geometry-optimised structure was subjected to a final iteration of rigid-body refinement, using the bootstrap method implemented in *TOPAS* to obtain error estimates on the atomic coordinates. The fit to the PXRD data are shown in Figure S7. Final fixed-cell and subsequent variable cell geometry optimizations of the reported structure were used as part of the validation process^57^.

The crystallographic information files (CIFs) for methionyl-methionine can also be obtained free of charge from The Cambridge Crystallographic Data Centre via http://www.ccdc.cam.ac.uk/data_request/cif; CCDC 2004931 (dd–ll) and CCDC 2004932 (dl–ld).

### Vibrational spectroscopy

INS spectra were recorded with the high resolution, broad band spectrometer TOSCA^58^ at the ISIS Pulsed Neutron and Muon Facility^59^. The samples, ~ 2 g, were loaded into indium sealed aluminium cans, cooled to < 20 K and measured for 1–2 h. The spectra are available at the INS database: http://wwwisis2.isis.rl.ac.uk/INSdatabase/. Infrared spectra were recorded at room temperature using a Bruker Vertex70 FTIR spectrometer, over the range 100–4000 cm^−1^ at 4 cm^−1^ resolution with a DLaTGS detector using 64 scans and the Bruker Diamond ATR. FT-Raman spectra were recorded with a Bruker MultiRam spectrometer using 1064 nm excitation, 4 cm^−1^ resolution, 500 mW laser power and 64 scans at room temperature.

### Transmission electron microscopy (TEM)

A Hitachi H7500 and a Jeol 2010F field emission transmission electron microscope were operated at 100 and 200 keV acceleration voltage, respectively. The sample was dispersed and transferred onto Holey Carbon Foil supported by a 200 mesh copper grid. The quality, stability and calibration of the TEM system were maintained by the use of the Magical No. 641 standard (Norrox Scientific Ltd., Beaver Pond, Ontario, Canada).

### Scanning electron microscopy (SEM)

A Jeol 7600F field emission instrument was used. A sample was dusted onto adhesive graphite tags.

### X-ray photoelectron spectroscopy (XPS)

A Thermo Fisher 250Xi instrument was operated using mono-chromatised Al Kα radiation, 20 eV pass energy A Met-Met sample was introduced into a differentially pumped pre-chamber as a loose powder. XPS spectra from an area of 1 mm^2^ size were recorded.

### Computational studies

The plane wave pseudopotential based program CASTEP was used for the calculation of the vibrational transition energies and their intensities in the harmonic approximation^60,61^. The starting structures were obtained from the literature (the ll form^22^) or this work (dd–ll and dl–ld racemates). The generalised gradient approximation (GGA) Perdew–Burke–Ernzerhof (PBE) functional was used in conjunction with optimised norm-conserving pseudopotentials. The plane wave cut-off was 1000 eV and Monkhorst–Pack grids of 4 × 4 × 12 (ll, 24 k-points), 10 × 2 × 4 (dd–ll racemate, 20 k-points), 4 × 8 × 3 (dl–ld racemate, 24 k-points). All of the calculations were converged to better than |0.01| eV Å^−1^. After geometry optimisation, the vibrational spectra were calculated in the harmonic approximation using density-functional perturbation-theory^62^. This procedure generates the vibrational eigenvalues and eigenvectors, which allows visualisation of the modes within Materials Studio^63^ and is also the information needed to calculate the INS spectrum using the program ACLIMAX^64^. We emphasise that the transition energies have *not* been scaled.

## Supplementary Information

Supplementary Information.

## Data Availability

The datasets supporting this article are available from the Science and Technology Facilities data repository eData at: https://edata.stfc.ac.uk/. The structures of dl–ld-methionyl-methionine and dd–ll-methionyl-methionine have also been deposited with the Cambridge Structural Database^39^. The deposit numbers are: CCDC 2004932 for dl–ld and CCDC 2004931 for dd–ll. The INS spectra of dl–ld and dd–ll are available from the INS database at: http://wwwisis2.isis.rl.ac.uk/INSdatabase/.
