# Supplementary material for: Structure and spectroscopy of methionyl-methionine for aquaculture
Source: Sci Rep. 2021 Jan 11;11:458. doi: 10.1038/s41598-020-80385-z (PMC7801548; doi:10.1038/s41598-020-80385-z)
Supplement: Supplementary file 1 — Supplementary Information. [file 41598_2020_80385_MOESM1_ESM.pdf]

**Supplementary information for:**

# Structure and spectroscopy of methionyl-methionine for aquaculture

Stewart F. Parker*[a], Nicholas P. Funnell,[a] Kenneth Shankland,[b] Elena A. Kabova,[b] Thomas Häußner,[c] Hans-Joachim Hasselbach,[c] Sascha Braune,[c] Christoph Kobler[c] and Peter W. Albers*[d]

[a]*ISIS Facility, STFC Rutherford Appleton Laboratory, Chilton, Didcot, OX11 0QX, UK.*
*E-mail: stewart.parker@stfc.ac.uk*

[b]*School of Pharmacy, University of Reading, Reading RG6 6AD, UK.*

[c] *Evonik Nutrition & Care GmbH, BL Animal Nutrition, Rodenbacher Chaussee 4, D-63457 Hanau-Wolfgang, Germany.*

[d]*Evonik Operations GmbH, Rodenbacher Chaussee 4, D-63457 Hanau-Wolfgang, Germany.*

Authors to whom correspondence should be addressed: stewart.parker@stfc.ac.uk and peterwalbers@web.de.

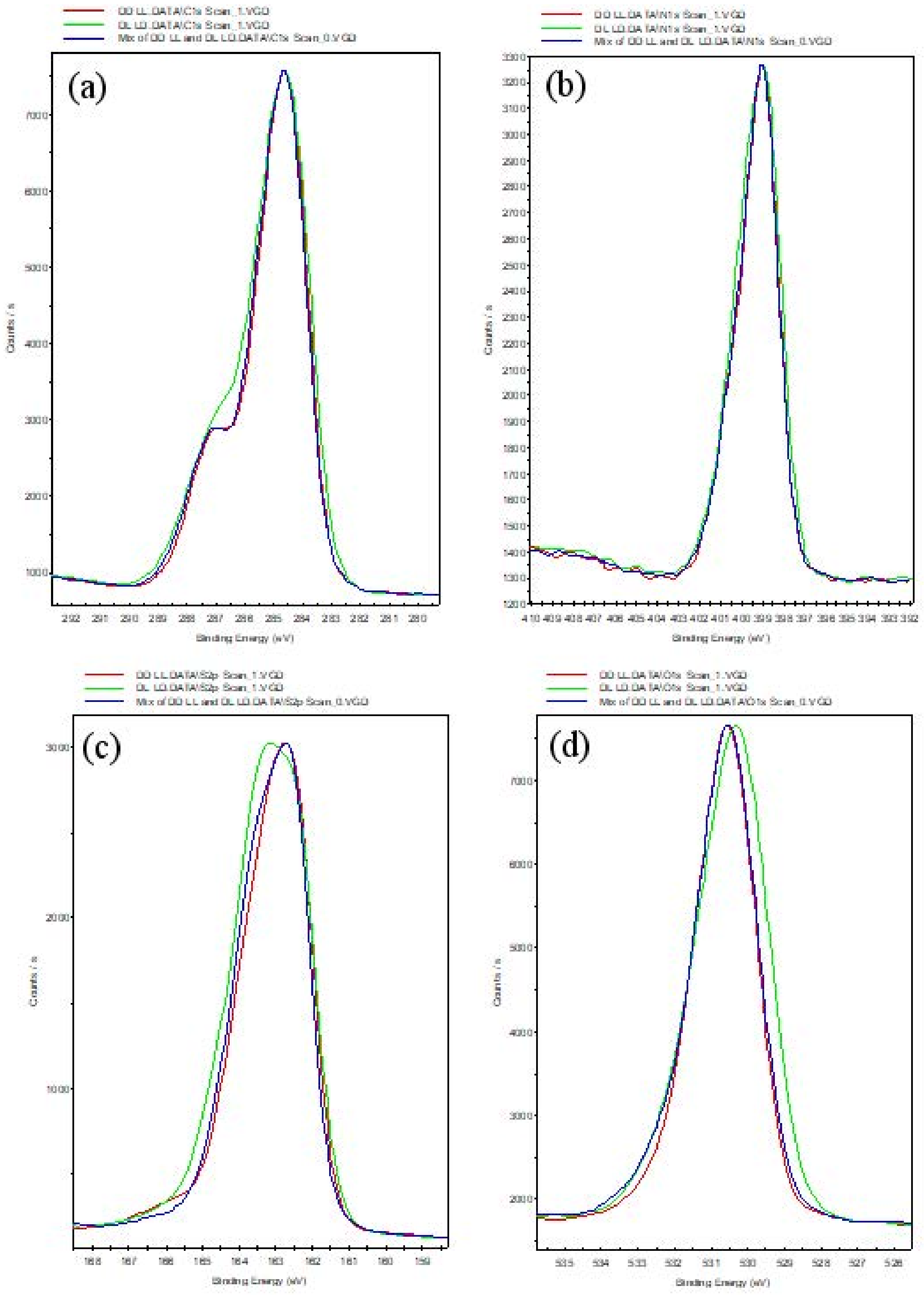


**Figure S1.** XPS of: left to right: C1s, N1s, S2p, O1s. Red: DD-LL, green: DL-LD, blue: mixture of DD-LL and DL-LD.

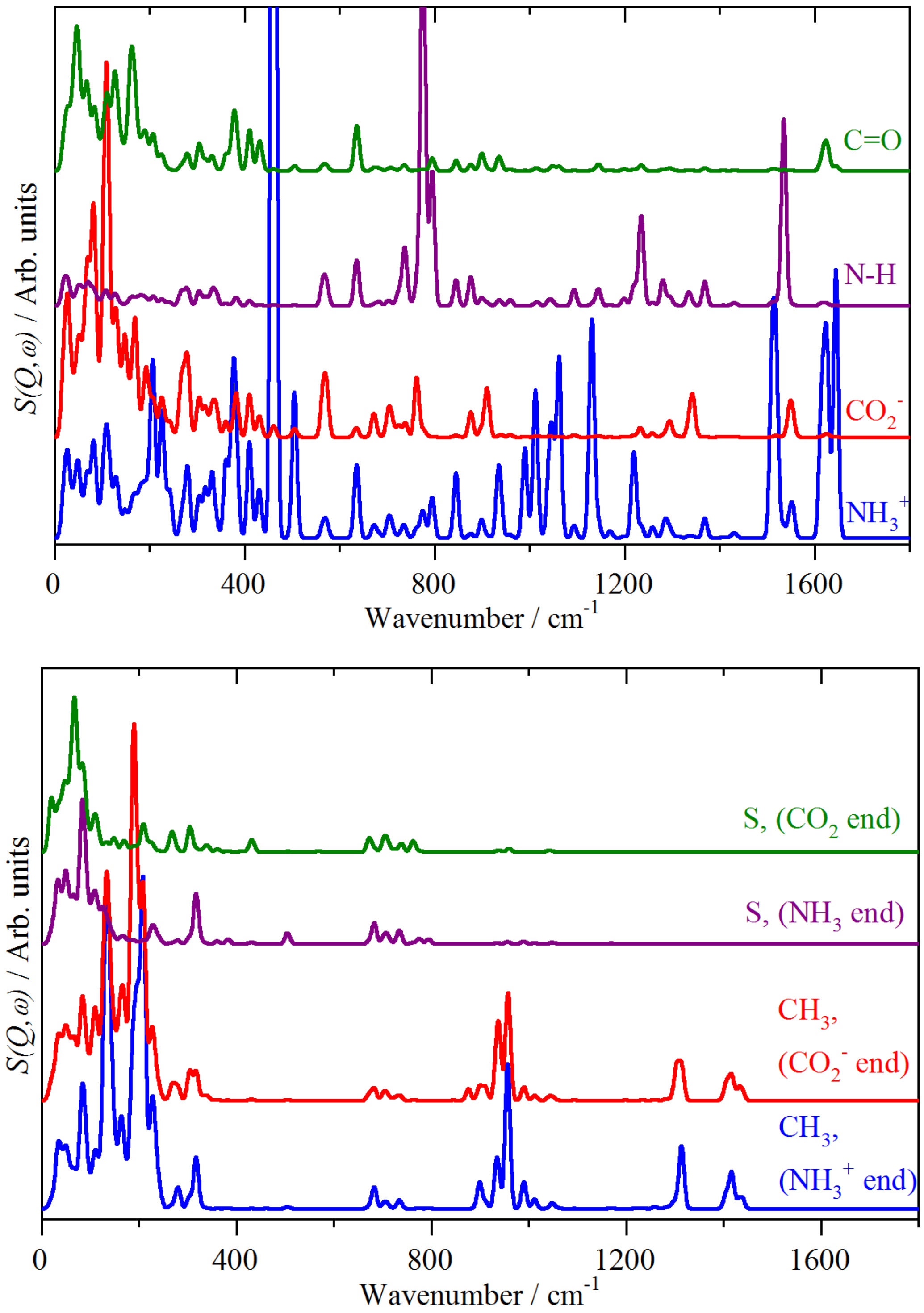


**Figure S2.** Pseudo-spectra of functional groups in the DD-LL racemate.

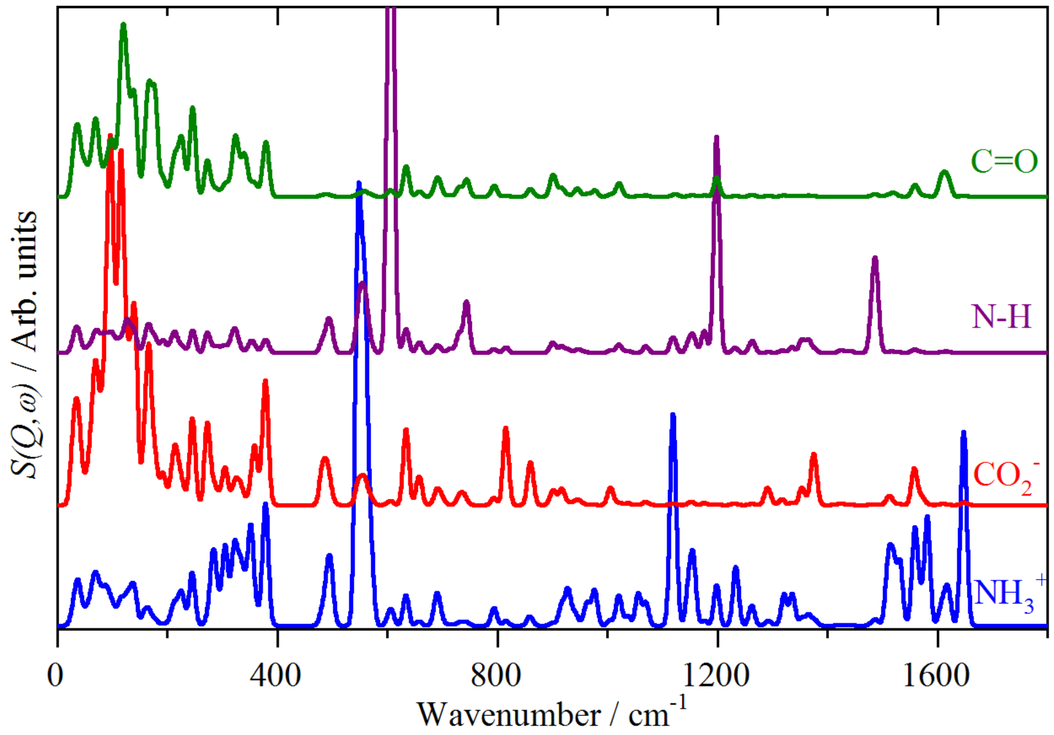


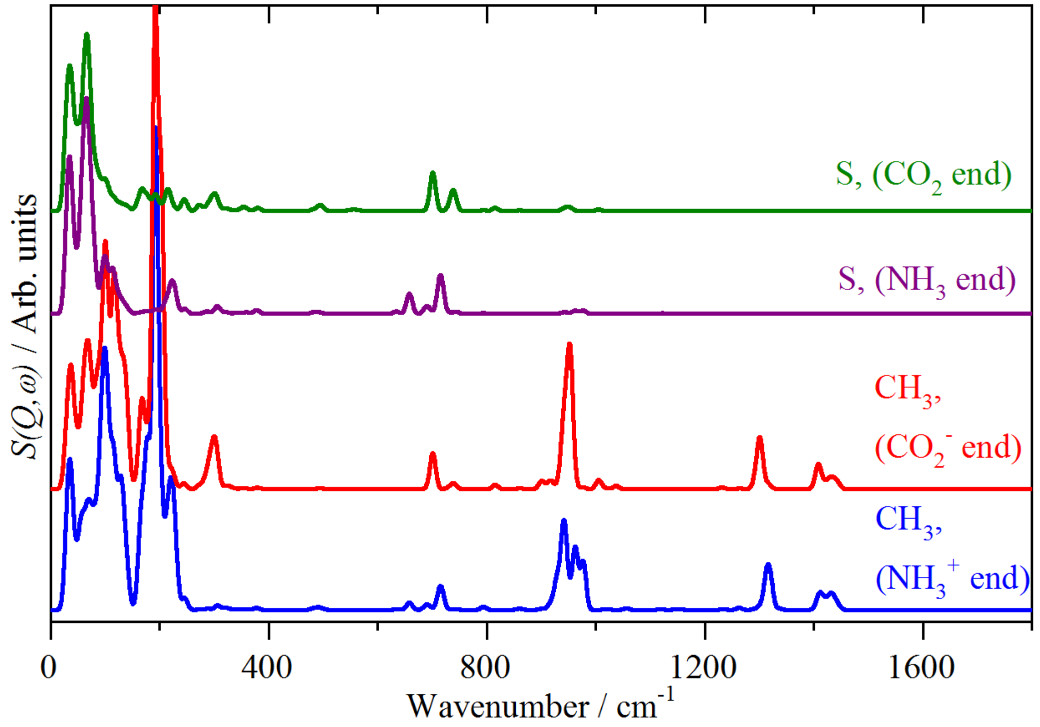


**Figure S3.** Pseudo-spectra of functional groups in the DL-LD racemate.

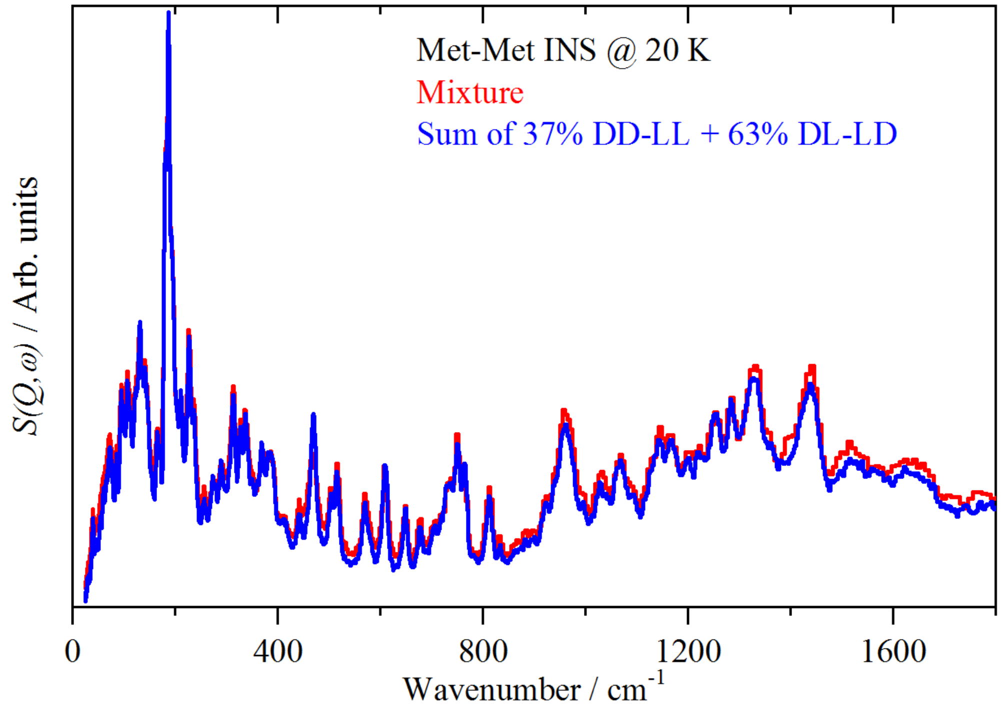


**Figure S4.** Comparison of the INS spectra of a physical mixture of the DD-LL and DL-LD racemates with a synthetic spectrum generated by scaled addition of the spectra of the individual components.

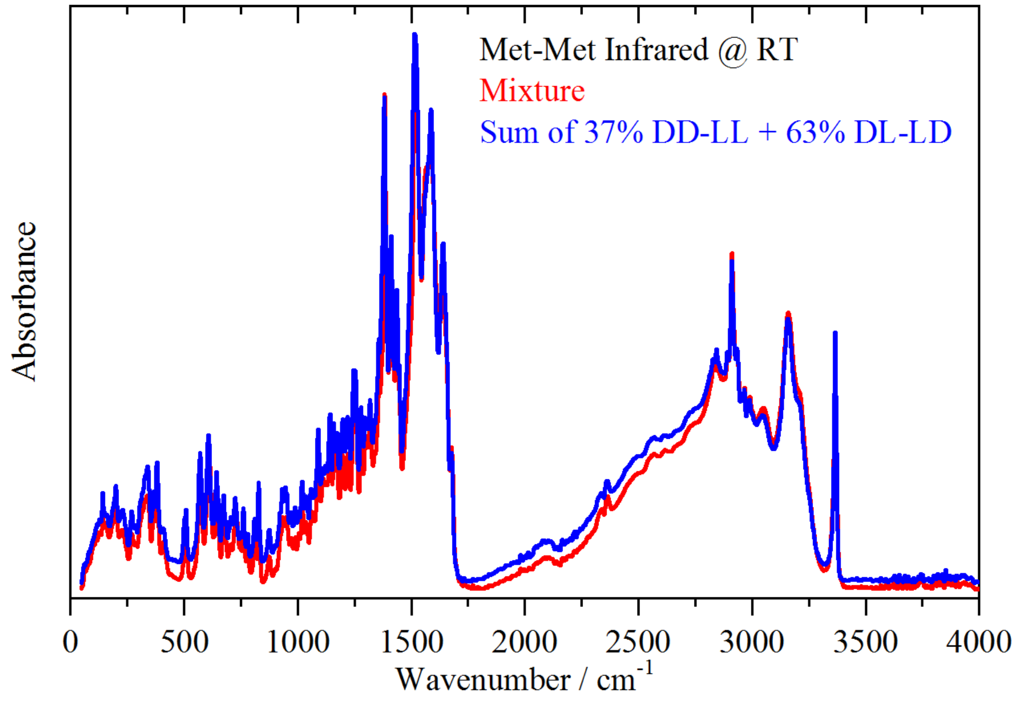


**Figure S5.** Comparison of the infrared spectra of a physical mixture of the DD-LL and DL-LD racemates with a synthetic spectrum generated by scaled addition of the spectra of the individual components.

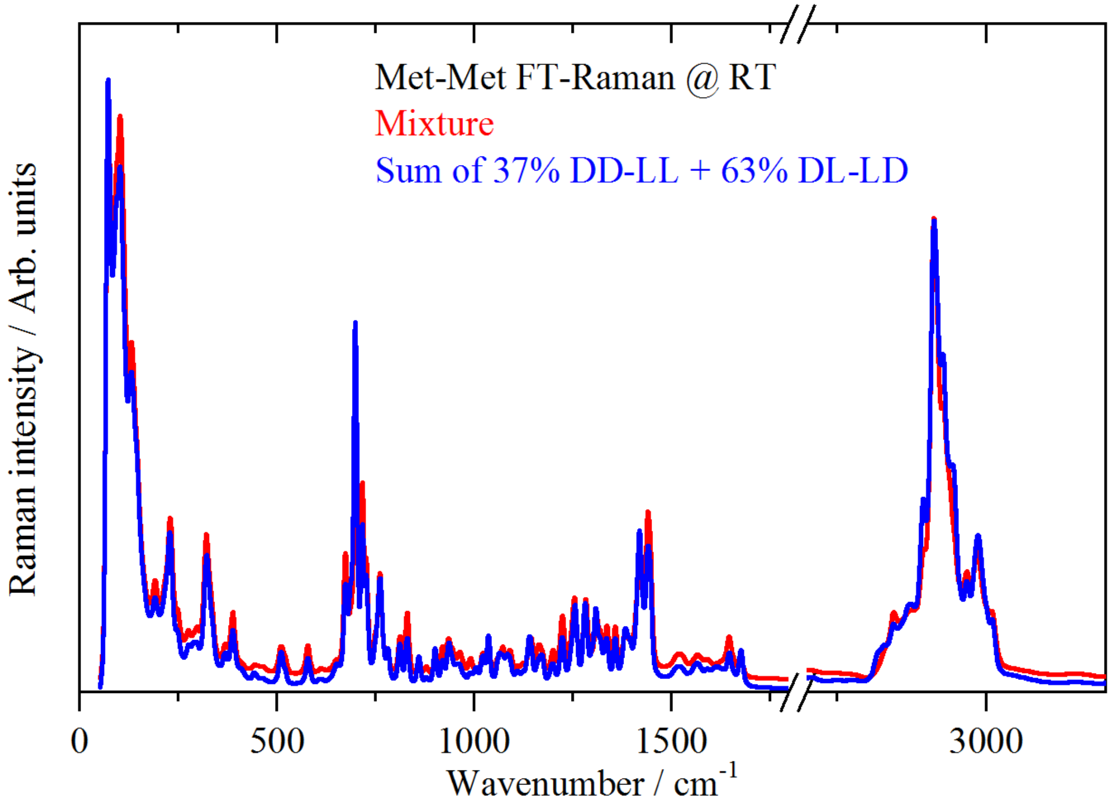


**Figure S6.** Comparison of the FT-Raman spectra of a physical mixture of the DD-LL and DL-LD racemates with a synthetic spectrum generated by scaled addition of the spectra of the individual components.

**Table S1.** Crystal data and structure refinement for methionyl-methionine

| | | |
|---|---|---|
| Identification code | racemic-D-methionyl-D-methionine | racemic-D-methionyl-L-methionine |
| Sample | Single crystal | Powder |
| Empirical formula | $C_{10}H_{20}N_2O_3S_2$ | $C_{10}H_{20}N_2O_3S_2$ |
| Formula weight | 280.40 | 280.40 |
| Temperature/K | 149.95(10) | 293 |
| Crystal system | monoclinic | monoclinic |
| Space group | $P2_1/c$ | $P2_1/c$ |
| a/Å | 5.33170(10) | 16.5974(3) |
| b/Å | 20.4441(2) | 5.3147(1) |
| c/Å | 12.34640(10) | 16.0288(3) |
| α/° | 90 | 90 |
| β/° | 95.5590(10) | 109.3409(13) |
| γ/° | 90 | 90 |
| Volume/Å$^3$ | 1339.45(3) | 1334.11(5) |
| Z, Z′ | 4, 1 | 4, 1 |
| $\rho_{calc}$ g/cm$^3$ | 1.390 | 1.396 |
| μ/mm$^{-1}$ | 3.616 | |
| F(000) | 600.0 | |
| Crystal size/mm$^3$ | 0.07 × 0.07 × 0.03 | |
| Radiation/Å | CuKα (λ = 1.54184) | CuK$\alpha_1$ (λ = 1.54056) |
| 2Θ range for data collection/° | 8.396 to 140.524 | 4 to 60 |
| Index ranges | $-6 \le h \le 6$, $-24 \le k \le 24$, $-15 \le l \le 15$ | |
| Reflections collected | 14958 | |
| Independent reflections | 2536 [$R_{int}$ = 0.0379, $R_{sigma}$ = 0.0209] | 396 |
| Data/restraints/parameters | 2536/0/172 | -/-/42 |
| Goodness-of-fit on F$^2$ | 1.046 | |
| Final *R* indexes [I>=2σ (I)] | $R_1$ = 0.0260, $wR_2$ = 0.0699 | |
| Final *R* indexes [all data] | $R_1$ = 0.0283, $wR_2$ = 0.0727 | |
| Largest diff. peak/hole / e Å$^{-3}$ | 0.32/-0.27 | |
| Profile *R* factors | | $R_{wp}$ (Pawley) = 1.83% $R_{wp}$ (Rietveld) = 3.74% |
| CCDC deposition number | 2004931 | 2004932 |

**Table S2.** Hydrogen bonding in the crystal structure of L-methionyl-L-methionine. The table shows calculated likelihoods, computed using CSD-derived models, for allowed coordination numbers for each donor and acceptor in the current structure. Bold highlighting indicates the likelihood for the observed coordination of the atom in that row. The mean of the highlighted coordination likelihoods is 0.524. The values of coordination likelihood identify N1 as a versatile donor with 3 and 4 coordination the most likely options. Overall, hydrogen bonding is consistent with expectations, with the notable exception of S2, which acts as an acceptor in a 'low likelihood' hydrogen bond.

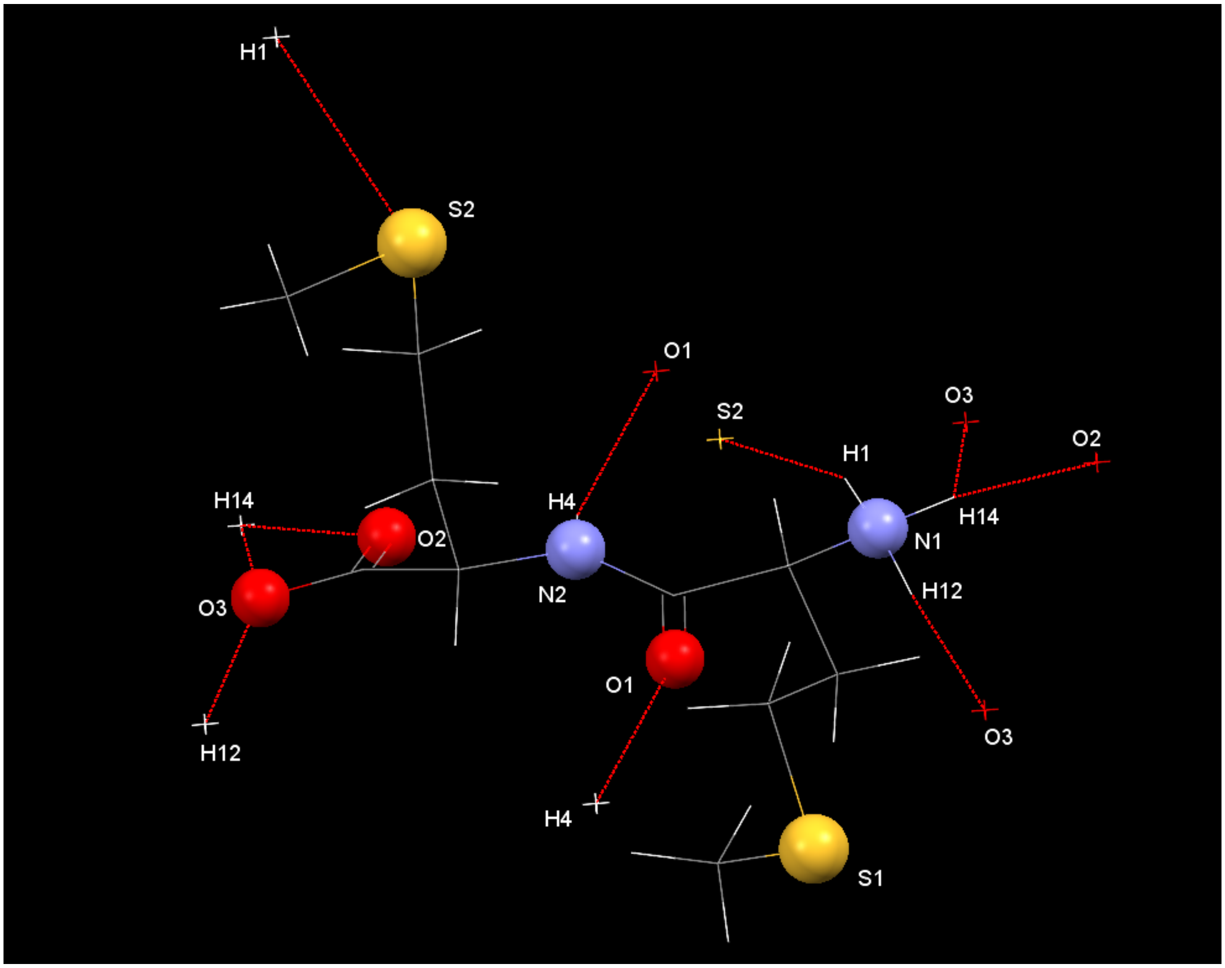


| Atom | Role | Observed coordination | Coordination likelihood = 0 | = 1 | = 2 | = 3 | = 4 | = 5 | >=6 |
|---|---|---|---|---|---|---|---|---|---|
| N1 | Donor | 4 | 0.000 | 0.000 | 0.014 | 0.603 | **0.304** | 0.050 | 0.029 |
| N2 | Donor | 1 | 0.418 | **0.573** | 0.009 | 0.000 | 0.000 | 0.000 | 0.000 |
| O1 | Acceptor | 1 | 0.161 | **0.800** | 0.038 | 0.001 | 0.000 | 0.000 | 0.000 |
| O2 | Acceptor | 1 | 0.021 | **0.392** | 0.532 | 0.053 | 0.003 | 0.000 | 0.000 |
| O3 | Acceptor | 2 | 0.017 | 0.312 | **0.596** | 0.070 | 0.004 | 0.000 | 0.000 |
| S1 | Acceptor | 0 | **0.975** | 0.025 | 0.000 | 0.000 | 0.000 | 0.000 | 0.000 |
| S2 | Acceptor | 1 | 0.975 | **0.025** | 0.000 | 0.000 | 0.000 | 0.000 | 0.000 |

**Table S3.** Hydrogen bonding in the crystal structure of *rac*-D-methionyl-D-methionine. The table shows calculated likelihoods, computed using CSD-derived models, for allowed coordination numbers for each donor and acceptor in the current structure. Bold highlighting indicates the likelihood for the observed coordination of the atom in that row. The mean of the highlighted coordination likelihoods is 0.444. In contrast to L-methionyl-L-methionine, O1 does *not* act as an acceptor and this contributes to the lower mean coordination likelihood; in common with L-methionyl-L-methionine, the values of observed coordination sum to 10 and a sulphur atom acts as an acceptor in a 'low likelihood' hydrogen bond.

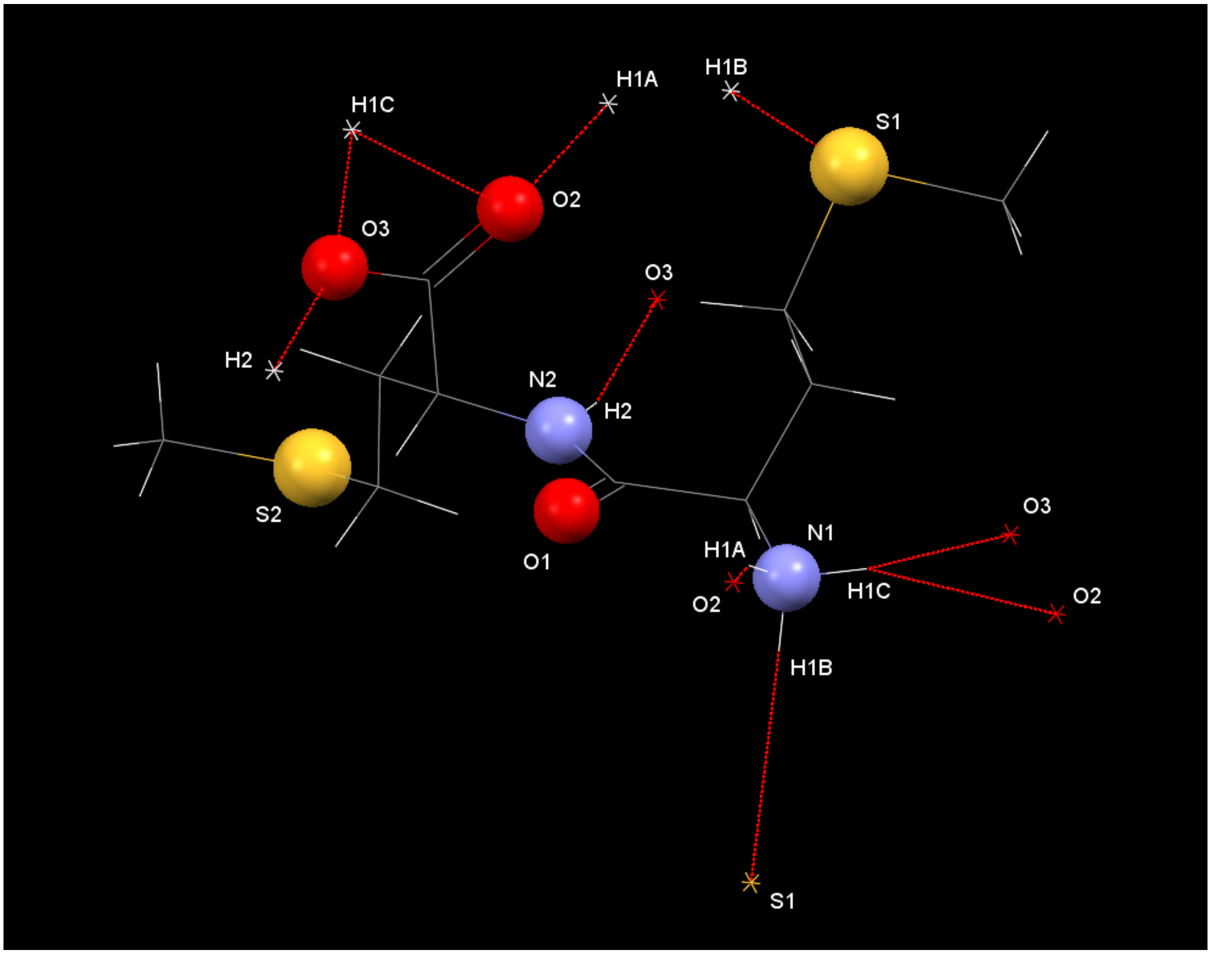


| Atom | Role | Observed coordination | Coordination likelihood = 0 | = 1 | = 2 | = 3 | = 4 | = 5 | >=6 |
|---|---|---|---|---|---|---|---|---|---|
| N1 | Donor | 4 | 0.001 | 0.001 | 0.024 | 0.630 | **0.286** | 0.038 | 0.021 |
| N2 | Donor | 1 | 0.329 | **0.662** | 0.009 | 0.000 | 0.000 | 0.000 | 0.000 |
| O1 | Acceptor | 0 | **0.176** | 0.790 | 0.033 | 0.001 | 0.000 | 0.000 | 0.000 |
| O2 | Acceptor | 2 | 0.031 | 0.600 | **0.342** | 0.025 | 0.001 | 0.000 | 0.000 |
| O3 | Acceptor | 2 | 0.015 | 0.252 | **0.639** | 0.089 | 0.005 | 0.000 | 0.000 |
| S1 | Acceptor | 1 | 0.975 | **0.025** | 0.000 | 0.000 | 0.000 | 0.000 | 0.000 |
| S2 | Acceptor | 0 | **0.975** | 0.025 | 0.000 | 0.000 | 0.000 | 0.000 | 0.000 |

**Table S4.** Hydrogen bonding in the crystal structure of *rac*-D-methionyl-L-methionine. The table shows calculated likelihoods, computed using CSD-derived models, for allowed coordination numbers for each donor and acceptor in the current structure. Bold highlighting indicates the likelihood for the observed coordination of the atom in that row. The mean of the highlighted coordination likelihoods is 0.616. In contrast to L-methionyl-L-methionine and racemic D-methionyl-D-methionine: the values of observed coordination sum to 8 (cf. 10 for both of the other structures); N2 does *not* act as a donor; neither S1 nor S2 acts as an acceptor.

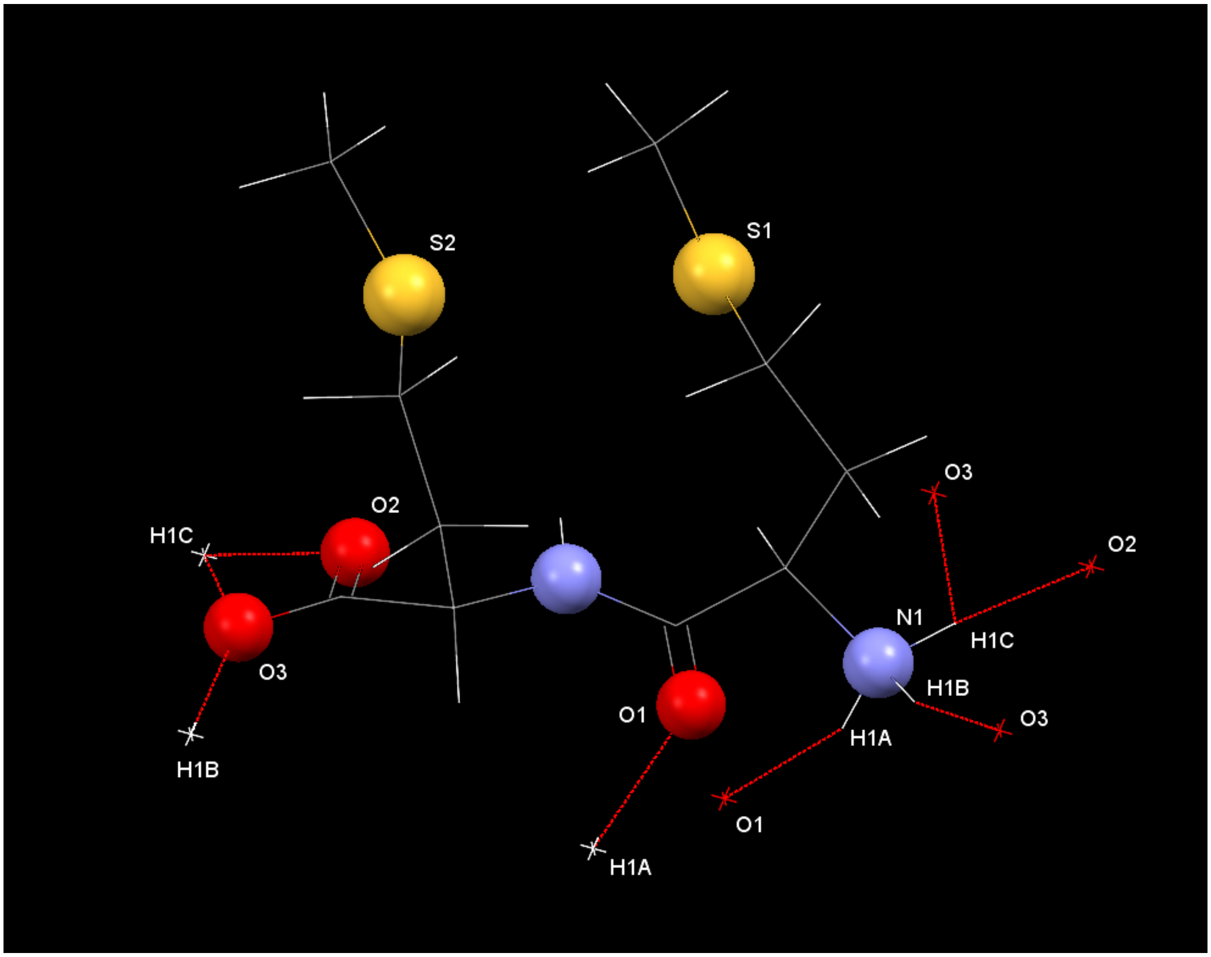


| Atom | Role | Observed coordination | Coordination likelihood = 0 | = 1 | = 2 | = 3 | = 4 | = 5 | >=6 |
|---|---|---|---|---|---|---|---|---|---|
| N1 | Donor | 4 | 0.000 | 0.000 | 0.018 | 0.615 | **0.297** | 0.045 | 0.025 |
| N2 | Donor | 0 | **0.322** | 0.669 | 0.009 | 0.000 | 0.000 | 0.000 | 0.000 |
| O1 | Acceptor | 1 | 0.158 | **0.802** | 0.039 | 0.001 | 0.000 | 0.000 | 0.000 |
| O2 | Acceptor | 1 | 0.020 | **0.375** | 0.546 | 0.056 | 0.003 | 0.000 | 0.000 |
| O3 | Acceptor | 2 | 0.019 | 0.348 | **0.568** | 0.062 | 0.004 | 0.000 | 0.000 |
| S1 | Acceptor | 0 | **0.975** | 0.025 | 0.000 | 0.000 | 0.000 | 0.000 | 0.000 |
| S2 | Acceptor | 0 | **0.975** | 0.025 | 0.000 | 0.000 | 0.000 | 0.000 | 0.000 |

**Figure S7.** Observed (open circles) and calculated (solid black line) PXRD data for the final Rietveld-refined crystal structure of racemic-D-methionyl-L-methionine. The difference-over-esd plot is shown at the bottom, reflection positions are indicated by vertical markers on the x-axis, and the inset shows the fit to the observed data at a high angle.

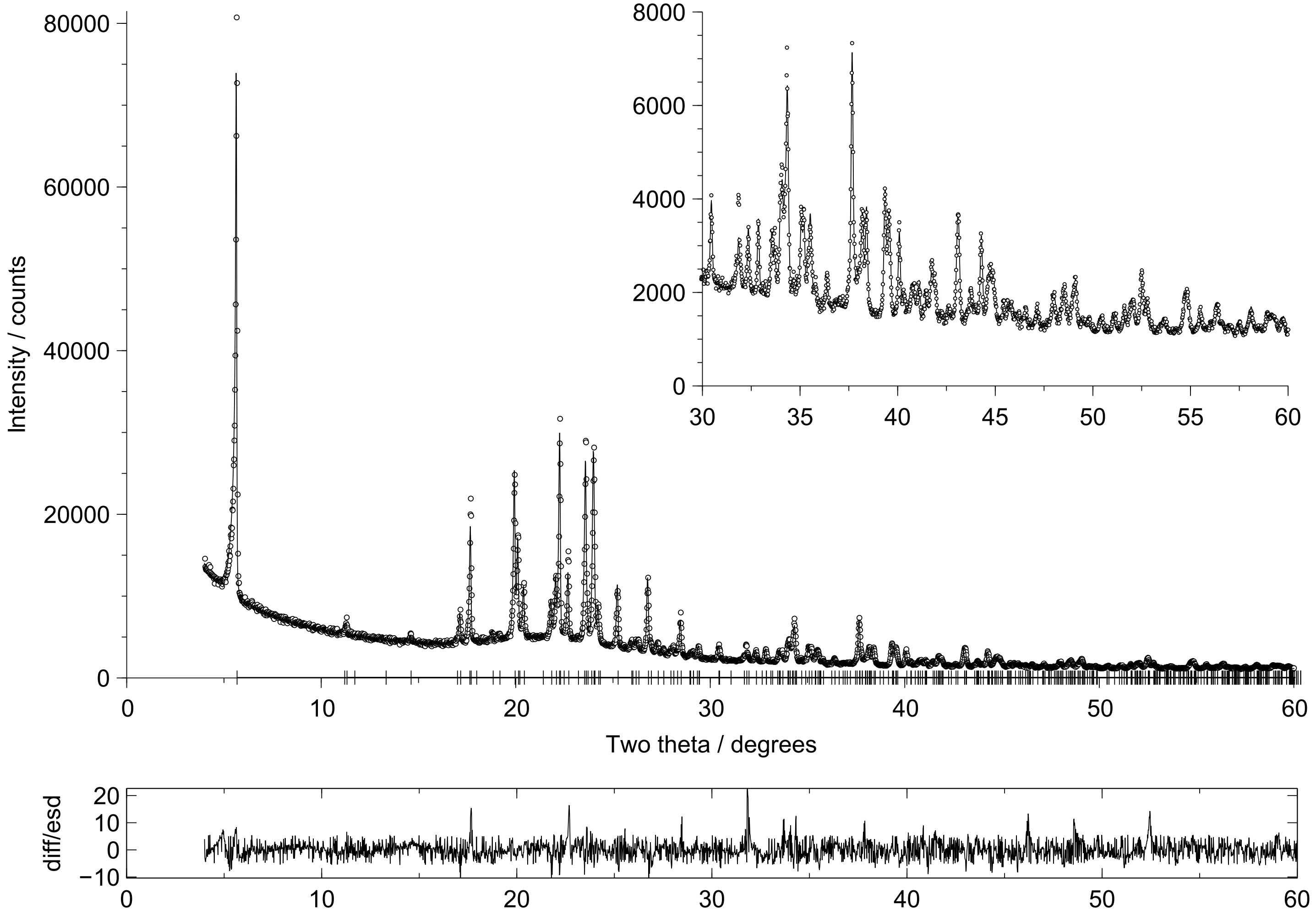

**Table S5.** Observed transition energies and approximate mode descriptions for DD-LL and DL-LD methionylmethionine.

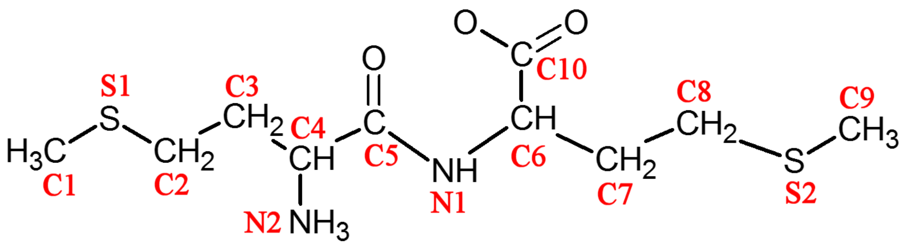


| DD-LL racemate | | | | DL-LD racemate | | | |
|---|---|---|---|---|---|---|---|
| INS[a] / cm$^{-1}$ | Raman / cm$^{-1}$ | Infrared / cm$^{-1}$ | Description | INS / cm$^{-1}$ | Raman / cm$^{-1}$ | Infrared / cm$^{-1}$ | Description |
| 183vs | | | C9 methyl torsion | 179vs | 190w | | C1 + C9 out-of-phase methyl torsion |
| 206vs | | | C1 methyl torsion | 187vs | | | C1 + C9 in-phase methyl torsion |
| 229s | 227m | 231w | C1-S1-C2 bend + C2-C3-C4 in-phase bend | 227s | 230s | | C1-S1-C2 bend |
| 276s | 277sh | 272w | C6-C7-C8 bend | 238m | | | C5-N1-C6 bend |
| 289s | 288w | 285w | S1-C2-C3 bend | 257w | 250w | | N1-C6-C7 bend |
| 311s | | | C9-S2-C8 bend | 315m | | 309w | S2-C8-C7 bend + C8-S2-C9 bend |
| 324s | 325m | 323w | C1-S1-C2 bend + C2-C3-C4 out-of-phase | 328m | 320s | 331m | |
| 342m | 338w | 342w | N2-C4-C5 bend | 338m | 333m | 342m | N2-C4-C5 bend |
| 368s | 368w | 365w | $NH_3$ torsion | 469s | | | $NH_3$ torsion |
| 384s | | | O=C5-C4 bend | 389m | 389m | 382s | Carboxylate rock |
| 410w | 409w | 410m | C3-C4-C5 bend | | | | |
| 442m | 444w | 444w | C5-N1-C6 bend | 504m | 506w | 507w | C2-C3-C4 bend |
| 514m | 510w | 510w | N2-C4-C3 bend | 519m | 520w | | N2-C4-C3 bend |
| 579m | 582w | 578m | N1-C6-C7 bend | 571m | 578w | 572s | C4-N2 stretch |

| | | | | | | | |
|---|---|---|---|---|---|---|---|
| 649m | 649m | 652w | C4-C5 stretch | 610s | 610vw | 609s | N1-H out-of-plane bend |
| | | | | 647m | | 643m | C3 methylene rock |
| 685w | 685m | 689m | C9-S2 + S2-C8 in phase stretch | 677m | 673m | 673s | C1-S1 + S1-C2 in phase stretch |
| 701w | 698w | 699vs | C1-S1 + S1-C2 in phase stretch | 706m | 703s | 704w | C4-C5 stretch |
| | | | | | 717s | | C9-S2 + S2-C8 in phase stretch |
| 732s | 728m | 727m | N1-H out-of-plane bend | 750m | 750w | | C7 + C8 out-of-phase methylene rock |
| 751m | 757m | 749w | $CO_2$- out-of-plane bend | 766m | 762m | 761m | C9-S2 + S2-C8 out-of-phase stretch |
| 765sh | 763m | 765w | Carboxylate O-C10-O in-plane bend | 813m | 812w | 811w | C3-C4 stretch |
| 813m | 812w | 808w | C2-C3-C4 bend | 835w | 830m | 831m | Carboxylate O-C10-O in-plane bend |
| 863m | 861m | | C3-C4 stretch | | | | |
| 899m | 902m | 901w | C7 + C8 in-phase methylene rock | 881w | 875m | 878w | C6-C7 + C7-C8 stretch |
| 924m | 922vw | 922w | C7 + C8 in-phase methylene twist | 918m | 920w | | C7 + C8 in-phase methylene rock |
| | 939w | 936w | C6-C10 stretch | | 946w | 943m | methyl rock |
| 961m,br | 957w | 954m | methyl rock | 961s,br | | | methyl rock |
| 1010m | 1006w | 1004m | C4-$NH_3$ stretch | 995m | 991w | 984w | C2 + C3 out-of-phase methylene rock |
| | | | | 1030m | 1022w | 1021w | C6-C10 stretch |
| | | | | 1045m | | 1040w | C4-$NH_3$ stretch |
| | | | | 1092m | 1091w | 1090m | C2-C3 stretch |

| | | | | | | | |
|---|---|---|---|---|---|---|---|
| | | | | 1128m | | | C2 methylene twist |
| 1020m | 1022w | 1022w | $NH_3$ rock | 1145m | 1144w | 1144m | $NH_3$ rock |
| 1037sh | 1038m | 1034w | C6-C7 stretch | | | | |
| 1069s | 1065w | 1061m | $NH_3$ rock | 1163m | 1164w | 1165w | $NH_3$ rock |
| 1141m | 1140m | 1139m | C2-C3 stretch | | | | |
| 1169m | 1169w | 1174w | C6-H bend | | | | |
| 1193m | 1193m | 1196w | C2 + C3 out-of-phase methylene twist | 1199w | 1201w | 1201m | C6-H bend along C6-C7 |
| 1258s | 1256s | 1258m | C8 methylene twist | 1221w | 1225m | 1222m | C4-C5 stretch |
| 1282w | 1278m | 1282m | C2 methylene wag | 1251m | 1252m | 1248m | C2 methylene wag |
| 1302m | 1298m | 1309m | methylene twist | 1283m | 1285m | 1283m | C2 + C3 in-phase methylene twist |
| 1330s | 1320m | 1331w | C7 methylene wag | 1320w | 1313w | 1315sh | C9-H sym methyl bend |
| | 1360w | 1357w | C3 methylene wag | 1334m | 1335w | 1338w | C3 methylene wag |
| | 1386m | 1380vs | Sym O-C-O stretch | | 1382w | 1382s | Sym O-C-O stretch |
| 1420m | 1420s | 1420m | C9-H asym methyl bend | | 1409w | 1409s | C3 methylene wag |
| 1439m | 1445w | 1445m | methylene scissors | 1437s | 1443s | 1436s | methylene scissors |
| | | 1511s | $NH_3$ sym bend | | 1523w | 1519vs | $NH_3$ sym bend |
| | 1564vw | 1557vs | Asym O-C-O stretch | | | 1586vs | Asym O-C-O stretch |
| | | 1606s | $NH_3$ asym bend and C5=O stretch | | 1648m | 1641vs | $NH_3$ asym bend and C5=O stretch |
| | 1678m | 1678s | C5=O stretch and $NH_3$ asym bend | | | | |
| | 2894s | 2890m | $CH_3$ sym stretch | | | | |
| | 2916s | 2917sh | $CH_2$ sym stretch | | 2910s | 2912s | $CH_2$ sym stretch |
| | 2927m | 2924m | $CH_2$ sym stretch | | 2929m | 2936m | $CH_2$ sym stretch |

| | | | | | | | |
|---|---|---|---|---|---|---|---|
| | 2946s | 2946sh | $CH_3$ asym stretch | | 2956sh | 2954w | $CH_3$ asym stretch |
| | 2967sh | 2966m | $CH_3$ asym stretch | | 2964m | | $CH_3$ asym stretch |
| | 2989s | 2990m | | | 2980m | | |
| | | 3051s,vbr | $NH_3$ asym stretch | | | 3149vs,br | $NH_3$ asym stretch |
| | 3218w | 3210vs | $NH_3$ asym stretch | | | | |
| | | | | | 3369vw | 3367vs | N1-H stretch |

[a]s = strong, m = medium, w = weak, br = broad, sh = shoulder, sym = symmetric, asym = asymmetric.

**Table S6: Graph set analysis of the crystal structure of L-methionyl-L-methionine as generated by Mercury 2020.2.0**

| Descriptor | Atoms | Symmetry operator | H-bond designation | Level | Period | No. of molecules |
|---|---|---|---|---|---|---|
| C1,1(9) | H1<br>S2-C9-C8-C7-N2-C1-C2-N1 | x,y,z<br>-1/2-x,-1-y,1/2+z | *a* | 1 | 1 | 2 |
| C1,1(8) | H12<br>O3-C6-C7-N2-C1-C2-N1 | x,y,z<br>-1/2+x,-1/2-y,-z | *b* | 1 | 1 | 2 |
| C1,1(8) | H14<br>O2-C6-C7-N2-C1-C2-N1 | x,y,z<br>-1/2+x,-1/2-y,-1-z | *c* | 1 | 1 | 2 |
| C1,1(4) | H4<br>O1-C1-N2-H4 | x,y,z<br>x,y,-1+z | *d* | 1 | 1 | 2 |
| C2,2(9) | H1<br>S2-C9-C8-C7-C6-O3<br>H12-N1 | x,y,z<br>-1/2-x,-1-y,1/2+z<br>-1-x,-1/2+y,1/2-z | *a*<br>*b* | 2 | 2 | 3 |
| C2,2(17) | H1<br>S2-C9-C8-C7-N2-C1-C2-N1-H12<br>O3-C6-C7-N2-C1-C2-N1 | x,y,z<br>-1/2-x,-1-y,1/2+z<br>-x,-1/2+y,1/2-z | *a*<br>*b* | 2 | 2 | 3 |
| C4,4(26) | H1<br>S2-C9-C8-C7-N2-C1-C2-N1-H12<br>O3-C6-C7-C8-C9-S2<br>H1-N1-C2-C1-N2-C7-C6-O3<br>H12-N1 | x,y,z<br>-1/2-x,-1-y,1/2+z<br>-x,-1/2+y,1/2-z<br>1/2+x,-3/2-y,1-z<br>1+x,-1+y,1+z | *a*<br>*b*<br>*a*<br>*b* | 2 | 4 | 5 |
| R6,6(42) | H1<br>S2-C9-C8-C7-N2-C1-C2-N1-H12<br>O3-C6-C7-N2-C1-C2-N1-H12<br>O3-C6-C7-C8-C9-S2<br>H1-N1-H12<br>O3-C6-C7-N2-C1-C2-N1-H12<br>O3-C6-C7-N2-C1-C2-N1 | x,y,z<br>-1/2-x,-1-y,1/2+z<br>-x,-1/2+y,1/2-z<br>1/2-x,-1-y,1/2+z<br>1+x,y,z<br>1/2+x,-1/2-y,-z<br>x,y,z | *a*<br>*b*<br>*b*<br>*a*<br>*b*<br>*b* | 2 | 6 | 6 |
| R6,6(44) | H1<br>S2-C9-C8-C7-N2-C1-C2-N1-H1<br>S2-C9-C8-C7-N2-C1-C2-N1-H12<br>O3-C6-C7-N2-C1-C2-N1-H1<br>S2-C9-C8-C7-N2-C1-C2-N1-H1<br>S2-C9-C8-C7-C6-O3<br>H12-N1 | x,y,z<br>-1/2-x,-1-y,1/2+z<br>x,y,1+z<br>-1/2+x,-1/2-y,1-z<br>-1-x,1/2+y,1/2-z<br>-1/2+x,-1/2-y,-z<br>x,y,z | *a*<br>*a*<br>*b*<br>*a*<br>*a*<br>*b* | 2 | 6 | 6 |

| | | | | | | |
|---|---|---|---|---|---|---|
| C2,2(9) | H1<br>S2-C9-C8-C7-C6-O2<br>H14-N1 | x,y,z<br>-1/2-x,-1-y,1/2+z<br>-1-x,-1/2+y,-1/2-z | *a*<br>*c* | 2 | 2 | 3 |
| C2,2(17) | H1<br>S2-C9-C8-C7-N2-C1-C2-N1-H14<br>O2-C6-C7-N2-C1-C2-N1 | x,y,z<br>-1/2-x,-1-y,1/2+z<br>-x,-1/2+y,-1/2-z | *a*<br>*c* | 2 | 2 | 3 |
| C4,4(26) | H1<br>S2-C9-C8-C7-N2-C1-C2-N1-H14<br>O2-C6-C7-C8-C9-S2<br>H1-N1-C2-C1-N2-C7-C6-O2<br>H14-N1 | x,y,z<br>-1/2-x,-1-y,1/2+z<br>-x,-1/2+y,-1/2-z<br>1/2+x,-3/2-y,-z<br>1+x,-1+y,1+z | *a*<br>*c*<br>*a*<br>*c* | 2 | 4 | 5 |
| R6,6(42) | H1<br>S2-C9-C8-C7-N2-C1-C2-N1-H14<br>O2-C6-C7-N2-C1-C2-N1-H14<br>O2-C6-C7-C8-C9-S2<br>H1-N1-H14<br>O2-C6-C7-N2-C1-C2-N1-H14<br>O2-C6-C7-N2-C1-C2-N1 | x,y,z<br>-1/2-x,-1-y,1/2+z<br>-x,-1/2+y,-1/2-z<br>1/2-x,-1-y,1/2+z<br>1+x,y,z<br>1/2+x,-1/2-y,-1-z<br>x,y,z | *a*<br>*c*<br>*c*<br>*a*<br>*c*<br>*c* | 2 | 6 | 6 |
| R6,6(44) | H1<br>S2-C9-C8-C7-N2-C1-C2-N1-H1<br>S2-C9-C8-C7-N2-C1-C2-N1-H14<br>O2-C6-C7-N2-C1-C2-N1-H1<br>S2-C9-C8-C7-N2-C1-C2-N1-H1<br>S2-C9-C8-C7-C6-O2<br>H14-N1 | x,y,z<br>-1/2-x,-1-y,1/2+z<br>x,y,1+z<br>-1/2+x,-1/2-y,-z<br>-1-x,1/2+y,-1/2-z<br>-1/2+x,-1/2-y,-1-z<br>x,y,z | *a*<br>*a*<br>*c*<br>*a*<br>*a*<br>*c* | 2 | 6 | 6 |
| C2,2(11) | H4<br>O1-C1-C2-N1-H1<br>S2-C9-C8-C7-N2 | x,y,z<br>x,y,-1+z<br>-1/2-x,-1-y,-1/2+z | *d*<br>*a* | 2 | 2 | 3 |
| C2,2(13) | H1<br>S2-C9-C8-C7-N2-C1-O1<br>H4-N2-C1-C2-N1 | x,y,z<br>-1/2-x,-1-y,1/2+z<br>-1/2-x,-1-y,3/2+z | *a*<br>*d* | 2 | 2 | 3 |
| R3,3(20) | H1<br>S2-C9-C8-C7-N2-C1-C2-N1-H1<br>S2-C9-C8-C7-N2-H4<br>O1-C1-C2-N1 | x,y,z<br>-1/2-x,-1-y,1/2+z<br>x,y,1+z<br>x,y,z | *a*<br>*a*<br>*d* | 2 | 3 | 3 |

| | | | | | | |
|---|---|---|---|---|---|---|
| R4,4(24) | H1<br>S2-C9-C8-C7-N2-H4<br>O1-C1-N2-C7-C8-C9-S2<br>H1-N1-C2-C1-O1<br>H4-N2-C1-C2-N1 | x,y,z<br>-1/2-x,-1-y,1/2+z<br>-1/2-x,-1-y,-1/2+z<br>x,y,-1+z<br>x,y,z | *a*<br>*d*<br>*a*<br>*d* | 2 | 4 | 4 |
| R5,5(28) | H1<br>S2-C9-C8-C7-N2-H4<br>O1-C1-N2-H4<br>O1-C1-C2-N1-H1<br>S2-C9-C8-C7-N2-C1-O1<br>H4-N2-C1-C2-N1 | x,y,z<br>-1/2-x,-1-y,1/2+z<br>-1/2-x,-1-y,-1/2+z<br>-1/2-x,-1-y,-3/2+z<br>x,y,-1+z<br>x,y,z | *a*<br>*d*<br>*d*<br>*a*<br>*d* | 2 | 5 | 5 |
| R6,6(32) | H1<br>S2-C9-C8-C7-N2-H4<br>O1-C1-N2-H4<br>O1-C1-N2-C7-C8-C9-S2<br>H1-N1-C2-C1-O1<br>H4-N2-C1-O1<br>H4-N2-C1-C2-N1 | x,y,z<br>-1/2-x,-1-y,1/2+z<br>-1/2-x,-1-y,-1/2+z<br>-1/2-x,-1-y,-3/2+z<br>x,y,-2+z<br>x,y,-1+z<br>x,y,z | *a*<br>*d*<br>*d*<br>*a*<br>*d*<br>*d* | 2 | 6 | 6 |
| C2,2(6) | H12<br>O3-C6-O2<br>H14-N1 | x,y,z<br>-1/2+x,-1/2-y,-z<br>x,y,1+z | *b*<br>*c* | 2 | 2 | 3 |
| C2,2(16) | H12<br>O3-C6-C7-N2-C1-C2-N1-H14<br>O3-C6-C7-N2-C1-C2-N1 | x,y,z<br>-1/2+x,-1/2-y,-z<br>-1+x,y,1+z | *b*<br>*c* | 2 | 2 | 3 |
| C4,4(22) | H12<br>O3-C6-C7-N2-C1-C2-N1-H14<br>O2-C6-O3<br>H12-N1-C2-C1-N2-C7-C6-O2<br>H14-N1 | x,y,z<br>-1/2+x,-1/2-y,-z<br>-1+x,y,1+z<br>-1/2+x,-1/2-y,1-z<br>x,y,2+z | *b*<br>*c*<br>*b*<br>*c* | 2 | 4 | 5 |
| R4,4(22) | H12<br>O3-C6-C7-N2-C1-C2-N1-H12<br>O3-C6-O2<br>H14-N1-C2-C1-N2-C7-C6-O2<br>H14-N1 | x,y,z<br>-1/2+x,-1/2-y,-z<br>-1+x,y,z<br>-1/2+x,-1/2-y,-1-z<br>x,y,z | *b*<br>*b*<br>*c*<br>*c* | 2 | 4 | 4 |

| | | | | | | |
|---|---|---|---|---|---|---|
| R6,6(38) | H12<br>O3-C6-C7-N2-C1-C2-N1-H12<br>O3-C6-C7-N2-C1-C2-N1-H14<br>O2-C6-O3<br>H12-N1-C2-C1-N2-C7-C6-O3<br>H12-N1-C2-C1-N2-C7-C6-O2<br>H14-N1 | x,y,z<br>-1/2+x,-1/2-y,-z<br>-1+x,y,z<br>-3/2+x,-1/2-y,-1-z<br>-1+x,y,-1+z<br>-1/2+x,-1/2-y,-1-z<br>x,y,z | *b*<br>*b*<br>*c*<br>*b*<br>*b*<br>*c* | 2 | 6 | 6 |
| R6,6(38) | H12<br>O3-C6-C7-N2-C1-C2-N1-H14<br>O2-C6-C7-N2-C1-C2-N1-H14<br>O2-C6-O3<br>H12-N1-C2-C1-N2-C7-C6-O2<br>H14-N1-C2-C1-N2-C7-C6-O2<br>H14-N1 | x,y,z<br>-1/2+x,-1/2-y,-z<br>-1+x,y,1+z<br>-3/2+x,-1/2-y,-z<br>-1+x,y,z<br>-1/2+x,-1/2-y,-1-z<br>x,y,z | *b*<br>*c*<br>*c*<br>*b*<br>*c*<br>*c* | 2 | 6 | 6 |
| C2,2(10) | H12<br>O3-C6-C7-N2-H4<br>O1-C1-C2-N1 | x,y,z<br>-1/2+x,-1/2-y,-z<br>-1/2+x,-1/2-y,1-z | *b*<br>*d* | 2 | 2 | 3 |
| C2,2(12) | H12<br>O3-C6-C7-N2-C1-O1<br>H4-N2-C1-C2-N1 | x,y,z<br>-1/2+x,-1/2-y,-z<br>-1/2+x,-1/2-y,-1-z | *b*<br>*d* | 2 | 2 | 3 |
| C4,4(22) | H12<br>O3-C6-C7-N2-H4<br>O1-C1-N2-C7-C6-O3<br>H12-N1-C2-C1-O1<br>H4-N2-C1-C2-N1 | x,y,z<br>-1/2+x,-1/2-y,-z<br>-1/2+x,-1/2-y,1-z<br>x,y,1+z<br>x,y,2+z | *b*<br>*d*<br>*b*<br>*d* | 2 | 4 | 5 |
| R4,4(22) | H12<br>O3-C6-C7-N2-H4<br>O1-C1-N2-C7-C6-O3<br>H12-N1-C2-C1-N2-H4<br>O1-C1-C2-N1 | x,y,z<br>-1/2+x,-1/2-y,-z<br>-1/2+x,-1/2-y,1-z<br>x,y,1+z<br>x,y,z | *b*<br>*d*<br>*b*<br>*d* | 2 | 4 | 4 |

| | | | | | | |
|---|---|---|---|---|---|---|
| R6,6(30) | H12<br>O3-C6-C7-N2-H4<br>O1-C1-N2-H4<br>O1-C1-N2-C7-C6-O3<br>H12-N1-C2-C1-N2-H4<br>O1-C1-N2-H4<br>O1-C1-C2-N1 | x,y,z<br>-1/2+x,-1/2-y,-z<br>-1/2+x,-1/2-y,1-z<br>-1/2+x,-1/2-y,2-z<br>x,y,2+z<br>x,y,1+z<br>x,y,z | *b*<br>*d*<br>*d*<br>*b*<br>*d*<br>*d* | 2 | 6 | 6 |
| R6,6(38) | H12<br>O3-C6-C7-N2-C1-C2-N1-H12<br>O3-C6-C7-N2-H4<br>O1-C1-N2-C7-C6-O3<br>H12-N1-C2-C1-N2-C7-C6-O3<br>H12-N1-C2-C1-O1<br>H4-N2-C1-C2-N1 | x,y,z<br>-1/2+x,-1/2-y,-z<br>-1+x,y,z<br>-1+x,y,-1+z<br>-1/2+x,-1/2-y,-1-z<br>x,y,-1+z<br>x,y,z | *b*<br>*b*<br>*d*<br>*b*<br>*b*<br>*d* | 2 | 6 | 6 |
| C2,2(10) | H14<br>O2-C6-C7-N2-H4<br>O1-C1-C2-N1 | x,y,z<br>-1/2+x,-1/2-y,-1-z<br>-1/2+x,-1/2-y,-z | *c*<br>*d* | 2 | 2 | 3 |
| C2,2(12) | H14<br>O2-C6-C7-N2-C1-O1<br>H4-N2-C1-C2-N1 | x,y,z<br>-1/2+x,-1/2-y,-1-z<br>-1/2+x,-1/2-y,-2-z | *c*<br>*d* | 2 | 2 | 3 |
| C4,4(22) | H14<br>O2-C6-C7-N2-H4<br>O1-C1-N2-C7-C6-O2<br>H14-N1-C2-C1-O1<br>H4-N2-C1-C2-N1 | x,y,z<br>-1/2+x,-1/2-y,-1-z<br>-1/2+x,-1/2-y,-z<br>x,y,1+z<br>x,y,2+z | *c*<br>*d*<br>*c*<br>*d* | 2 | 4 | 5 |
| R4,4(22) | H14<br>O2-C6-C7-N2-H4<br>O1-C1-N2-C7-C6-O2<br>H14-N1-C2-C1-N2-H4<br>O1-C1-C2-N1 | x,y,z<br>-1/2+x,-1/2-y,-1-z<br>-1/2+x,-1/2-y,-z<br>x,y,1+z<br>x,y,z | *c*<br>*d*<br>*c*<br>*d* | 2 | 4 | 4 |

| | | | | | | |
|---|---|---|---|---|---|---|
| R6,6(30) | H14<br>O2-C6-C7-N2-H4<br>O1-C1-N2-H4<br>O1-C1-N2-C7-C6-O2<br>H14-N1-C2-C1-N2-H4<br>O1-C1-N2-H4<br>O1-C1-C2-N1 | x,y,z<br>-1/2+x,-1/2-y,-1-z<br>-1/2+x,-1/2-y,-z<br>-1/2+x,-1/2-y,1-z<br>x,y,2+z<br>x,y,1+z<br>x,y,z | *c*<br>*d*<br>*d*<br>*c*<br>*d*<br>*d* | 2 | 6 | 6 |
| R6,6(38) | H14<br>O2-C6-C7-N2-C1-C2-N1-H14<br>O2-C6-C7-N2-H4<br>O1-C1-N2-C7-C6-O2<br>H14-N1-C2-C1-N2-C7-C6-O2<br>H14-N1-C2-C1-O1<br>H4-N2-C1-C2-N1 | x,y,z<br>-1/2+x,-1/2-y,-1-z<br>-1+x,y,z<br>-1+x,y,-1+z<br>-1/2+x,-1/2-y,-2-z<br>x,y,-1+z<br>x,y,z | *c*<br>*c*<br>*d*<br>*c*<br>*c*<br>*d* | 2 | 6 | 6 |

***Descriptor*** Standard notation for graph set type. The capitalised letter identifies the type of pattern: C (= infinite chain), R (= ring); the next two numbers give the number of acceptors and the number of donors in the pattern; the final number identifies the total number of atoms in a pattern.

***Atoms*** The CIF labels for the atoms in the pattern. Each line of atoms for a given set lists the participating atoms of **one** individual molecule in that set; the corresponding ***symmetry operator*** identifies the symmetry relationship of the participating atoms relative to the first atom in the set.

***H-bond designation*** The letters indicate the symmetry equivalence of the H-bonds within a pattern – thus, all H-bonds labelled *a* are symmetry equivalent, but are not symmetry equivalent to H-bonds labelled *b*.

***Level*** Indicates the number of symmetry-independent H-bonds bonds that compose a given pattern. The graph set analysis was conducted with the *Mercury* default that finds graph sets up to level 2.

***Period*** The size of the repeat unit (number of hydrogen bonds) for ring and chain patterns.

***No. of molecules*** The total number of molecules participating in a pattern.

Taking R4,4(22) as an example, the ring contains 22 atoms in the following sequence:

H14(x,y,z)··*c*··O2-C6-C7-N2-H4··*d*··O1-C1-N2-C7-C6-O2··*c*··H14-N1-C2-C1-N2-H··*c*··O1-C1-C2-N1(x,y,z)

The sets are most easily visualised by loading the structure CIF into Mercury and selecting “Calculate, Graph Sets…”.

**Table S7: Graph set analysis of the crystal structure of *rac*-D-methionyl-D-methionine as generated by Mercury 2020.2.0**

| Descriptor | Atoms | Symmetry operator | H-bond designation | Level | Period | No. of molecules |
|---|---|---|---|---|---|---|
| C1,1(5) | H2<br>O3-C7-C6-N2 | x,y,z<br>1+x,y,z | *a* | 1 | 1 | 2 |
| C1,1(8) | H1A<br>O2-C7-C6-N2-C5-C4-N1 | x,y,z<br>x,1.5-y,1/2+z | *b* | 1 | 1 | 2 |
| C1,1(6) | H1B<br>S1-C2-C3-C4-N1 | x,y,z<br>x,1.5-y,1/2+z | *c* | 1 | 1 | 2 |
| C1,1(8) | H1C<br>O3-C7-C6-N2-C5-C4-N1 | x,y,z<br>1+x,1.5-y,1/2+z | *d* | 1 | 1 | 2 |
| C1,1(8) | H1C<br>O2-C7-C6-N2-C5-C4-N1 | x,y,z<br>1+x,1.5-y,1/2+z | *e* | 1 | 1 | 2 |
| C2,2(9) | H2<br>O3-C7-O2<br>H1A-N1-C4-C5-N2 | x,y,z<br>1+x,y,z<br>1+x,1.5-y,-1/2+z | *a*<br>*b* | 2 | 2 | 3 |
| C2,2(13) | H2<br>O3-C7-C6-N2-C5-C4-N1-H1A<br>O2-C7-C6-N2 | x,y,z<br>1+x,y,z<br>1+x,1.5-y,1/2+z | *a*<br>*b* | 2 | 2 | 3 |
| R4,4(22) | H2<br>O3-C7-C6-N2-C5-C4-N1-H1A<br>O2-C7-O3<br>H2-N2-C6-C7-O2<br>H1A-N1-C4-C5-N2 | x,y,z<br>1+x,y,z<br>1+x,1.5-y,1/2+z<br>x,1.5-y,1/2+z<br>x,y,z | *a*<br>*b*<br>*a*<br>*b* | 2 | 4 | 4 |
| R6,6(32) | H2<br>O3-C7-C6-N2-H2<br>O3-C7-C6-N2-C5-C4-N1-H1A<br>O2-C7-O3<br>H2-N2-C6-C7-O3<br>H2-N2-C6-C7-O2<br>H1A-N1-C4-C5-N2 | x,y,z<br>1+x,y,z<br>2+x,y,z<br>2+x,1.5-y,1/2+z<br>1+x,1.5-y,1/2+z<br>x,1.5-y,1/2+z<br>x,y,z | *a*<br>*a*<br>*b*<br>*a*<br>*a*<br>*b* | 2 | 6 | 6 |
| R6,6(38) | H2<br>O3-C7-C6-N2-C5-C4-N1-H1A<br>O2-C7-C6-N2-C5-C4-N1-H1A<br>O2-C7-O3<br>H2-N2-C6-C7-O2<br>H1A-N1-C4-C5-N2-C6-C7-O2<br>H1A-N1-C4-C5-N2 | x,y,z<br>1+x,y,z<br>1+x,1.5-y,1/2+z<br>1+x,y,1+z<br>x,y,1+z<br>x,1.5-y,1/2+z<br>x,y,z | *a*<br>*b*<br>*b*<br>*a*<br>*b*<br>*b* | 2 | 6 | 6 |

| | | | | | | |
|---|---|---|---|---|---|---|
| C2,2(15) | H2<br>O3-C7-C6-N2-C5-C4-C3-C2-S1<br>H1B-N1-C4-C5-N2 | x,y,z<br>1+x,y,z<br>1+x,1.5-y,-1/2+z | *a*<br>*c* | 2 | 2 | 3 |
| C2,2(15) | H2<br>O3-C7-C6-N2-C5-C4-N1-H1B<br>S1-C2-C3-C4-C5-N2 | x,y,z<br>1+x,y,z<br>1+x,1.5-y,1/2+z | *a*<br>*c* | 2 | 2 | 3 |
| R4,4(30) | H2<br>O3-C7-C6-N2-C5-C4-N1-H1B<br>S1-C2-C3-C4-C5-N2-C6-C7-O3<br>H2-N2-C5-C4-C3-C2-S1<br>H1B-N1-C4-C5-N2 | x,y,z<br>1+x,y,z<br>1+x,1.5-y,1/2+z<br>x,1.5-y,1/2+z<br>x,y,z | *a*<br>*c*<br>*a*<br>*c* | 2 | 4 | 4 |
| R6,6(40) | H2<br>O3-C7-C6-N2-H2<br>O3-C7-C6-N2-C5-C4-N1-H1B<br>S1-C2-C3-C4-C5-N2-C6-C7-O3<br>H2-N2-C6-C7-O3<br>H2-N2-C5-C4-C3-C2-S1<br>H1B-N1-C4-C5-N2 | x,y,z<br>1+x,y,z<br>2+x,y,z<br>2+x,1.5-y,1/2+z<br>1+x,1.5-y,1/2+z<br>x,1.5-y,1/2+z<br>x,y,z | *a*<br>*a*<br>*c*<br>*a*<br>*a*<br>*c* | 2 | 6 | 6 |
| R6,6(42) | H2<br>O3-C7-C6-N2-C5-C4-N1-H1B<br>S1-C2-C3-C4-N1-H1B<br>S1-C2-C3-C4-C5-N2-C6-C7-O3<br>H2-N2-C5-C4-C3-C2-S1<br>H1B-N1-C4-C3-C2-S1<br>H1B-N1-C4-C5-N2 | x,y,z<br>1+x,y,z<br>1+x,1.5-y,1/2+z<br>1+x,y,1+z<br>x,y,1+z<br>x,1.5-y,1/2+z<br>x,y,z | *a*<br>*c*<br>*c*<br>*a*<br>*c*<br>*c* | 2 | 6 | 6 |
| C1,2(7) | H2<br>O3<br>H1C-N1-C4-C5-N2 | x,y,z<br>1+x,y,z<br>x,1.5-y,-1/2+z | *a*<br>*d* | 2 | 2 | 3 |
| C2,2(13) | H2<br>O3-C7-C6-N2-C5-C4-N1-H1C<br>O3-C7-C6-N2 | x,y,z<br>1+x,y,z<br>2+x,1.5-y,1/2+z | *a*<br>*d* | 2 | 2 | 3 |
| R3,4(20) | H2<br>O3-C7-C6-N2-C5-C4-N1-H1C<br>O3<br>H2-N2-C6-C7-O3<br>H1C-N1-C4-C5-N2 | x,y,z<br>1+x,y,z<br>2+x,1.5-y,1/2+z<br>1+x,1.5-y,1/2+z<br>x,y,z | *a*<br>*d*<br>*a*<br>*d* | 2 | 4 | 4 |

| | | | | | | |
|---|---|---|---|---|---|---|
| R5,6(30) | H2<br>O3-C7-C6-N2-H2<br>O3-C7-C6-N2-C5-C4-N1-H1C<br>O3<br>H2-N2-C6-C7-O3<br>H2-N2-C6-C7-O3<br>H1C-N1-C4-C5-N2 | x,y,z<br>1+x,y,z<br>2+x,y,z<br>3+x,1.5-y,1/2+z<br>2+x,1.5-y,1/2+z<br>1+x,1.5-y,1/2+z<br>x,y,z | *a*<br>*a*<br>*d*<br>*a*<br>*a*<br>*d* | 2 | 6 | 6 |
| R5,6(36) | H2<br>O3-C7-C6-N2-C5-C4-N1-H1C<br>O3-C7-C6-N2-C5-C4-N1-H1C<br>O3<br>H2-N2-C6-C7-O3<br>H1C-N1-C4-C5-N2-C6-C7-O3<br>H1C-N1-C4-C5-N2 | x,y,z<br>1+x,y,z<br>2+x,1.5-y,1/2+z<br>3+x,y,1+z<br>2+x,y,1+z<br>1+x,1.5-y,1/2+z<br>x,y,z | *a*<br>*d*<br>*d*<br>*a*<br>*d*<br>*d* | 2 | 6 | 6 |
| C2,2(9) | H2<br>O3-C7-O2<br>H1C-N1-C4-C5-N2 | x,y,z<br>1+x,y,z<br>x,1.5-y,-1/2+z | *a*<br>*e* | 2 | 2 | 3 |
| C2,2(13) | H2<br>O3-C7-C6-N2-C5-C4-N1-H1C<br>O2-C7-C6-N2 | x,y,z<br>1+x,y,z<br>2+x,1.5-y,1/2+z | *a*<br>*e* | 2 | 2 | 3 |
| R4,4(22) | H2<br>O3-C7-C6-N2-C5-C4-N1-H1C<br>O2-C7-O3<br>H2-N2-C6-C7-O2<br>H1C-N1-C4-C5-N2 | x,y,z<br>1+x,y,z<br>2+x,1.5-y,1/2+z<br>1+x,1.5-y,1/2+z<br>x,y,z | *a*<br>*e*<br>*a*<br>*e* | 2 | 4 | 4 |
| R6,6(32) | H2<br>O3-C7-C6-N2-H2<br>O3-C7-C6-N2-C5-C4-N1-H1C<br>O2-C7-O3<br>H2-N2-C6-C7-O3<br>H2-N2-C6-C7-O2<br>H1C-N1-C4-C5-N2 | x,y,z<br>1+x,y,z<br>2+x,y,z<br>3+x,1.5-y,1/2+z<br>2+x,1.5-y,1/2+z<br>1+x,1.5-y,1/2+z<br>x,y,z | *a*<br>*a*<br>*e*<br>*a*<br>*a*<br>*e* | 2 | 6 | 6 |

| | | | | | | |
|---|---|---|---|---|---|---|
| R6,6(38) | H2<br>O3-C7-C6-N2-C5-C4-N1-H1C<br>O2-C7-C6-N2-C5-C4-N1-H1C<br>O2-C7-O3<br>H2-N2-C6-C7-O2<br>H1C-N1-C4-C5-N2-C6-C7-O2<br>H1C-N1-C4-C5-N2 | x,y,z<br>1+x,y,z<br>2+x,1.5-y,1/2+z<br>3+x,y,1+z<br>2+x,y,1+z<br>1+x,1.5-y,1/2+z<br>x,y,z | *a*<br>*e*<br>*e*<br>*a*<br>*e*<br>*e* | 2 | 6 | 6 |
| R2,2(12) | H1A<br>O2-C7-C6-N2-C5-C4-C3-C2-S1<br>H1B-N1 | x,y,z<br>x,1.5-y,1/2+z<br>x,y,z | *b*<br>*c* | 2 | 2 | 2 |
| C2,2(14) | H1A<br>O2-C7-C6-N2-C5-C4-N1-H1B<br>S1-C2-C3-C4-N1 | x,y,z<br>x,1.5-y,1/2+z<br>x,y,1+z | *b*<br>*c* | 2 | 2 | 3 |
| C2,2(6) | H1A<br>O2-C7-O3<br>H1C-N1 | x,y,z<br>x,1.5-y,1/2+z<br>-1+x,y,z | *b*<br>*d* | 2 | 2 | 3 |
| C2,2(16) | H1A<br>O2-C7-C6-N2-C5-C4-N1-H1C<br>O3-C7-C6-N2-C5-C4-N1 | x,y,z<br>x,1.5-y,1/2+z<br>1+x,y,1+z | *b*<br>*d* | 2 | 2 | 3 |
| R4,4(22) | H1A<br>O2-C7-C6-N2-C5-C4-N1-H1C<br>O3-C7-O2<br>H1A-N1-C4-C5-N2-C6-C7-O3<br>H1C-N1 | x,y,z<br>x,1.5-y,1/2+z<br>1+x,y,1+z<br>1+x,1.5-y,1/2+z<br>x,y,z | *b*<br>*d*<br>*b*<br>*d* | 2 | 4 | 4 |
| R6,6(38) | H1A<br>O2-C7-C6-N2-C5-C4-N1-H1A<br>O2-C7-C6-N2-C5-C4-N1-H1C<br>O3-C7-O2<br>H1A-N1-C4-C5-N2-C6-C7-O2<br>H1A-N1-C4-C5-N2-C6-C7-O3<br>H1C-N1 | x,y,z<br>x,1.5-y,1/2+z<br>x,y,1+z<br>1+x,1.5-y,1.5+z<br>1+x,y,1+z<br>1+x,1.5-y,1/2+z<br>x,y,z | *b*<br>*b*<br>*d*<br>*b*<br>*b*<br>*d* | 2 | 6 | 6 |

| | | | | | | |
|---|---|---|---|---|---|---|
| R6,6(38) | H1A<br>O2-C7-C6-N2-C5-C4-N1-H1C<br>O3-C7-C6-N2-C5-C4-N1-H1C<br>O3-C7-O2<br>H1A-N1-C4-C5-N2-C6-C7-O3<br>H1C-N1-C4-C5-N2-C6-C7-O3<br>H1C-N1 | x,y,z<br>x,1.5-y,1/2+z<br>1+x,y,1+z<br>2+x,1.5-y,1.5+z<br>2+x,y,1+z<br>1+x,1.5-y,1/2+z<br>x,y,z | *b*<br>*d*<br>*d*<br>*b*<br>*d*<br>*d* | 2 | 6 | 6 |
| C1,2(4) | H1A<br>O2<br>H1C-N1 | x,y,z<br>x,1.5-y,1/2+z<br>-1+x,y,z | *b*<br>*e* | 2 | 2 | 3 |
| C2,2(16) | H1A<br>O2-C7-C6-N2-C5-C4-N1-H1C<br>O2-C7-C6-N2-C5-C4-N1 | x,y,z<br>x,1.5-y,1/2+z<br>1+x,y,1+z | *b*<br>*e* | 2 | 2 | 3 |
| R3,4(20) | H1A<br>O2-C7-C6-N2-C5-C4-N1-H1C<br>O2<br>H1A-N1-C4-C5-N2-C6-C7-O2<br>H1C-N1 | x,y,z<br>x,1.5-y,1/2+z<br>1+x,y,1+z<br>1+x,1.5-y,1/2+z<br>x,y,z | *b*<br>*e*<br>*b*<br>*e* | 2 | 4 | 4 |
| R5,6(36) | H1A<br>O2-C7-C6-N2-C5-C4-N1-H1A<br>O2-C7-C6-N2-C5-C4-N1-H1C<br>O2<br>H1A-N1-C4-C5-N2-C6-C7-O2<br>H1A-N1-C4-C5-N2-C6-C7-O2<br>H1C-N1 | x,y,z<br>x,1.5-y,1/2+z<br>x,y,1+z<br>1+x,1.5-y,1.5+z<br>1+x,y,1+z<br>1+x,1.5-y,1/2+z<br>x,y,z | *b*<br>*b*<br>*e*<br>*b*<br>*b*<br>*e* | 2 | 6 | 6 |
| R5,6(36) | H1A<br>O2-C7-C6-N2-C5-C4-N1-H1C<br>O2-C7-C6-N2-C5-C4-N1-H1C<br>O2<br>H1A-N1-C4-C5-N2-C6-C7-O2<br>H1C-N1-C4-C5-N2-C6-C7-O2<br>H1C-N1 | x,y,z<br>x,1.5-y,1/2+z<br>1+x,y,1+z<br>2+x,1.5-y,1.5+z<br>2+x,y,1+z<br>1+x,1.5-y,1/2+z<br>x,y,z | *b*<br>*e*<br>*e*<br>*b*<br>*e*<br>*e* | 2 | 6 | 6 |
| C2,2(12) | H1B<br>S1-C2-C3-C4-C5-N2-C6-C7-O3<br>H1C-N1 | x,y,z<br>x,1.5-y,1/2+z<br>-1+x,y,z | *c*<br>*d* | 2 | 2 | 3 |

| | | | | | | |
|---|---|---|---|---|---|---|
| C2,2(14) | H1B<br>S1-C2-C3-C4-N1-H1C<br>O3-C7-C6-N2-C5-C4-N1 | x,y,z<br>x,1.5-y,1/2+z<br>1+x,y,1+z | *c*<br>*d* | 2 | 2 | 3 |
| R4,4(26) | H1B<br>S1-C2-C3-C4-N1-H1C<br>O3-C7-C6-N2-C5-C4-C3-C2-S1<br>H1B-N1-C4-C5-N2-C6-C7-O3<br>H1C-N1 | x,y,z<br>x,1.5-y,1/2+z<br>1+x,y,1+z<br>1+x,1.5-y,1/2+z<br>x,y,z | *c*<br>*d*<br>*c*<br>*d* | 2 | 4 | 4 |
| R6,6(38) | H1B<br>S1-C2-C3-C4-N1-H1B<br>S1-C2-C3-C4-N1-H1C<br>O3-C7-C6-N2-C5-C4-C3-C2-S1<br>H1B-N1-C4-C3-C2-S1<br>H1B-N1-C4-C5-N2-C6-C7-O3<br>H1C-N1 | x,y,z<br>x,1.5-y,1/2+z<br>x,y,1+z<br>1+x,1.5-y,1.5+z<br>1+x,y,1+z<br>1+x,1.5-y,1/2+z<br>x,y,z | *c*<br>*c*<br>*d*<br>*c*<br>*c*<br>*d* | 2 | 6 | 6 |
| R6,6(42) | H1B<br>S1-C2-C3-C4-N1-H1C<br>O3-C7-C6-N2-C5-C4-N1-H1C<br>O3-C7-C6-N2-C5-C4-C3-C2-S1<br>H1B-N1-C4-C5-N2-C6-C7-O3<br>H1C-N1-C4-C5-N2-C6-C7-O3<br>H1C-N1 | x,y,z<br>x,1.5-y,1/2+z<br>1+x,y,1+z<br>2+x,1.5-y,1.5+z<br>2+x,y,1+z<br>1+x,1.5-y,1/2+z<br>x,y,z | *c*<br>*d*<br>*d*<br>*c*<br>*d*<br>*d* | 2 | 6 | 6 |
| C2,2(12) | H1B<br>S1-C2-C3-C4-C5-N2-C6-C7-O2<br>H1C-N1 | x,y,z<br>x,1.5-y,1/2+z<br>-1+x,y,z | *c*<br>*e* | 2 | 2 | 3 |
| C2,2(14) | H1B<br>S1-C2-C3-C4-N1-H1C<br>O2-C7-C6-N2-C5-C4-N1 | x,y,z<br>x,1.5-y,1/2+z<br>1+x,y,1+z | *c*<br>*e* | 2 | 2 | 3 |
| R4,4(26) | H1B<br>S1-C2-C3-C4-N1-H1C<br>O2-C7-C6-N2-C5-C4-C3-C2-S1<br>H1B-N1-C4-C5-N2-C6-C7-O2<br>H1C-N1 | x,y,z<br>x,1.5-y,1/2+z<br>1+x,y,1+z<br>1+x,1.5-y,1/2+z<br>x,y,z | *c*<br>*e*<br>*c*<br>*e* | 2 | 4 | 4 |

| | | | | | | |
|---|---|---|---|---|---|---|
| R6,6(38) | H1B<br>S1-C2-C3-C4-N1-H1B<br>S1-C2-C3-C4-N1-H1C<br>O2-C7-C6-N2-C5-C4-C3-C2-S1<br>H1B-N1-C4-C3-C2-S1<br>H1B-N1-C4-C5-N2-C6-C7-O2<br>H1C-N1 | x,y,z<br>x,1.5-y,1/2+z<br>x,y,1+z<br>1+x,1.5-y,1.5+z<br>1+x,y,1+z<br>1+x,1.5-y,1/2+z<br>x,y,z | *c*<br>*c*<br>*e*<br>*c*<br>*c*<br>*e* | 2 | 6 | 6 |
| R6,6(42) | H1B<br>S1-C2-C3-C4-N1-H1C<br>O2-C7-C6-N2-C5-C4-N1-H1C<br>O2-C7-C6-N2-C5-C4-C3-C2-S1<br>H1B-N1-C4-C5-N2-C6-C7-O2<br>H1C-N1-C4-C5-N2-C6-C7-O2<br>H1C-N1 | x,y,z<br>x,1.5-y,1/2+z<br>1+x,y,1+z<br>2+x,1.5-y,1.5+z<br>2+x,y,1+z<br>1+x,1.5-y,1/2+z<br>x,y,z | *c*<br>*e*<br>*e*<br>*c*<br>*e*<br>*e* | 2 | 6 | 6 |
| R2,1(4) | H1C<br>O3-C7-O2 | x,y,z<br>1+x,1.5-y,1/2-z | *d*<br>*e* | 2 | 2 | 2 |
| C2,2(16) | H1C<br>O3-C7-C6-N2-C5-C4-N1-H1C<br>O2-C7-C6-N2-C5-C4-N1 | x,y,z<br>1+x,1.5-y,1/2+z<br>2+x,y,1+z | *d*<br>*e* | 2 | 2 | 3 |

***Descriptor*** Standard notation for graph set type. The capitalised letter identifies the type of pattern: C (= infinite chain), R (= ring); the next two numbers give the number of acceptors and the number of donors in the pattern; the final number identifies the total number of atoms in a pattern.

***Atoms*** The CIF labels for the atoms in the pattern. Each line of atoms for a given set lists the participating atoms of **one** individual molecule in that set; the corresponding ***symmetry operator*** identifies the symmetry relationship of the participating atoms relative to the first atom in the set.

***H-bond designation*** The letters indicate the symmetry equivalence of the H-bonds within a pattern – thus, all H-bonds labelled *a* are symmetry equivalent, but are not symmetry equivalent to H-bonds labelled *b*.

***Level*** Indicates the number of symmetry-independent H-bonds bonds that compose a given pattern. The graph set analysis was conducted with the *Mercury* default that finds graph sets up to level 2.

***Period*** The size of the repeat unit (number of hydrogen bonds) for ring and chain patterns.

***No. of molecules*** The total number of molecules participating in a pattern
The sets are most easily visualised by loading the structure CIF into Mercury and selecting “Calculate, Graph Sets…”.

**Table S8: Graph set analysis of the crystal structure of *rac*-D-methionyl-L-methionine as generated by Mercury 2020.2.0**

| Descriptor | Atoms | Symmetry operator | H-bond designation | Level | Period | No. of molecules |
|---|---|---|---|---|---|---|
| C1,1(5) | H1A<br>O1-C5-C4-N1 | x,y,z<br>1-x,1/2+y,1/2-z | *a* | 1 | 1 | 2 |
| C1,1(8) | H1B<br>O3-C7-C6-N2-C5-C4-N1 | x,y,z<br>x,-1/2-y,1/2+z | *b* | 1 | 1 | 2 |
| C1,1(8) | H1C<br>O2-C7-C6-N2-C5-C4-N1 | x,y,z<br>x,1/2-y,1/2+z | *c* | 1 | 1 | 2 |
| R4,4(18) | H1A<br>O1-C5-N2-C6-C7-O3<br>H1B-N1-H1A<br>O1-C5-N2-C6-C7-O3<br>H1B-N1 | x,y,z<br>1-x,1/2+y,1/2-z<br>1-x,-y,1-z<br>x,-1/2-y,1/2+z<br>x,y,z | *a*<br>*b*<br>*a*<br>*b* | 2 | 2 | 4 |
| C4,4(22) | H1A<br>O1-C5-C4-N1-H1B<br>O3-C7-C6-N2-C5-O1<br>H1A-N1-C4-C5-N2-C6-C7-O3<br>H1B-N1 | x,y,z<br>1-x,1/2+y,1/2-z<br>1-x,-y,-z<br>x,1/2-y,-1/2+z<br>x,1+y,-1+z | *a*<br>*b*<br>*a*<br>*b* | 2 | 4 | 5 |
| R4,4(26) | H1A<br>O1-C5-C4-N1-H1B<br>O3-C7-C6-N2-C5-C4-N1-H1A<br>O1-C5-C4-N1-H1B<br>O3-C7-C6-N2-C5-C4-N1 | x,y,z<br>1-x,1/2+y,1/2-z<br>1-x,-y,-z<br>x,-1/2-y,-1/2+z<br>x,y,z | *a*<br>*b*<br>*a*<br>*b* | 2 | 2 | 4 |
| R6,6(32) | H1A<br>O1-C5-C4-N1-H1A<br>O1-C5-C4-N1-H1B<br>O3-C7-C6-N2-C5-C4-N1-H1A<br>O1-C5-C4-N1-H1A<br>O1-C5-N2-C6-C7-O3<br>H1B-N1 | x,y,z<br>1-x,1/2+y,1/2-z<br>x,1+y,z<br>x,1/2-y,1/2+z<br>1-x,-y,1-z<br>x,-1/2-y,1/2+z<br>x,y,z | *a*<br>*a*<br>*b*<br>*a*<br>*a*<br>*b* | 2 | 6 | 6 |
| R6,6(38) | H1A<br>O1-C5-C4-N1-H1B<br>O3-C7-C6-N2-C5-C4-N1-H1B<br>O3-C7-C6-N2-C5-O1<br>H1A-N1-H1B<br>O3-C7-C6-N2-C5-C4-N1-H1B<br>O3-C7-C6-N2-C5-C4-N1 | x,y,z<br>1-x,1/2+y,1/2-z<br>1-x,-y,-z<br>1-x,1/2+y,-1/2-z<br>x,y,-1+z<br>x,-1/2-y,-1/2+z<br>x,y,z | *a*<br>*b*<br>*b*<br>*a*<br>*b*<br>*b* | 2 | 6 | 6 |

| | | | | | | |
|---|---|---|---|---|---|---|
| R4,4(18) | H1A<br>O1-C5-N2-C6-C7-O2<br>H1C-N1-H1A<br>O1-C5-N2-C6-C7-O2<br>H1C-N1 | x,y,z<br>1-x,1/2+y,1/2-z<br>1-x,1-y,1-z<br>x,1/2-y,1/2+z<br>x,y,z | *a*<br>*c*<br>*a*<br>*c* | 2 | 2 | 4 |
| C4,4(22) | H1A<br>O1-C5-C4-N1-H1C<br>O2-C7-C6-N2-C5-O1<br>H1A-N1-C4-C5-N2-C6-C7-O2<br>H1C-N1 | x,y,z<br>1-x,1/2+y,1/2-z<br>1-x,1-y,-z<br>x,1.5-y,-1/2+z<br>x,1+y,-1+z | *a*<br>*c*<br>*a*<br>*c* | 2 | 4 | 5 |
| R4,4(26) | H1A<br>O1-C5-C4-N1-H1C<br>O2-C7-C6-N2-C5-C4-N1-H1A<br>O1-C5-C4-N1-H1C<br>O2-C7-C6-N2-C5-C4-N1 | x,y,z<br>1-x,1/2+y,1/2-z<br>1-x,1-y,-z<br>x,1/2-y,-1/2+z<br>x,y,z | *a*<br>*c*<br>*a*<br>*c* | 2 | 2 | 4 |
| R6,6(32) | H1A<br>O1-C5-C4-N1-H1A<br>O1-C5-C4-N1-H1C<br>O2-C7-C6-N2-C5-C4-N1-H1A<br>O1-C5-C4-N1-H1A<br>O1-C5-N2-C6-C7-O2<br>H1C-N1 | x,y,z<br>1-x,1/2+y,1/2-z<br>x,1+y,z<br>x,1.5-y,1/2+z<br>1-x,1-y,1-z<br>x,1/2-y,1/2+z<br>x,y,z | *a*<br>*a*<br>*c*<br>*a*<br>*a*<br>*c* | 2 | 6 | 6 |
| R6,6(38) | H1A<br>O1-C5-C4-N1-H1C<br>O2-C7-C6-N2-C5-C4-N1-H1C<br>O2-C7-C6-N2-C5-O1<br>H1A-N1-H1C<br>O2-C7-C6-N2-C5-C4-N1-H1C<br>O2-C7-C6-N2-C5-C4-N1 | x,y,z<br>1-x,1/2+y,1/2-z<br>1-x,1-y,-z<br>1-x,1/2+y,-1/2-z<br>x,y,-1+z<br>x,1/2-y,-1/2+z<br>x,y,z | *a*<br>*c*<br>*c*<br>*a*<br>*c*<br>*c* | 2 | 6 | 6 |
| C2,2(6) | H1B<br>O3-C7-O2<br>H1C-N1 | x,y,z<br>x,-1/2-y,1/2+z<br>x,-1+x,z | *b*<br>*c* | 2 | 2 | 3 |
| C2,2(16) | H1B<br>O3-C7-C6-N2-C5-C4-N1-H1C<br>O2-C7-C6-N2-C5-C4-N1 | x,y,z<br>x,-1/2-y,1/2+z<br>x,-1+y,1+z | *b*<br>*c* | 2 | 2 | 3 |

| | | | | | | |
|---|---|---|---|---|---|---|
| C4,4(22) | H1B<br>O3-C7-C6-N2-C5-C4-N1-H1C<br>O2-C7-O3<br>H1B-N1-C4-C5-N2-C6-C7-O2<br>H1C-N1 | x,y,z<br>x,-1/2-y,1/2+z<br>x,-1+y,1+z<br>x,-1.5-y,1/2+z<br>x,-2+y,z | *b*<br>*c*<br>*b*<br>*c* | 2 | 4 | 5 |
| R4,4(22) | H1B<br>O3-C7-C6-N2-C5-C4-N1-H1B<br>O3-C7-O2<br>H1C-N1-C4-C5-N2-C6-C7-O2<br>H1C-N1 | x,y,z<br>x,-1/2-y,1/2+z<br>x,y,1+z<br>x,1/2-y,1/2+z<br>x,y,z | *b*<br>*b*<br>*c*<br>*c* | 2 | 4 | 4 |
| R6,6(38) | H1B<br>O3-C7-C6-N2-C5-C4-N1-H1B<br>O3-C7-C6-N2-C5-C4-N1-H1C<br>O2-C7-O3<br>H1B-N1-C4-C5-N2-C6-C7-O3<br>H1B-N1-C4-C5-N2-C6-C7-O2<br>H1C-N1 | x,y,z<br>x,-1/2-y,1/2+z<br>x,y,1+z<br>x,1/2-y,1.5+z<br>x,1+y,1+z<br>x,1/2-y,1/2+z<br>x,y,z | *b*<br>*b*<br>*c*<br>*b*<br>*b*<br>*c* | 2 | 6 | 6 |
| R6,6(38) | H1B<br>O3-C7-C6-N2-C5-C4-N1-H1C<br>O2-C7-C6-N2-C5-C4-N1-H1C<br>O2-C7-O3<br>H1B-N1-C4-C5-N2-C6-C7-O2<br>H1C-N1-C4-C5-N2-C6-C7-O2<br>H1C-N1 | x,y,z<br>x,-1/2-y,1/2+z<br>x,-1+y,1+z<br>x,-1/2-y,1.5+z<br>x,y,1+z<br>x,1/2-y,1/2+z<br>x,y,z | *b*<br>*c*<br>*c*<br>*b*<br>*c*<br>*c* | 2 | 6 | 6 |

***Descriptor*** Standard notation for graph set type. The capitalised letter identifies the type of pattern: C (= infinite chain), R (= ring); the next two numbers give the number of acceptors and the number of donors in the pattern; the final number identifies the total number of atoms in a pattern.

***Atoms*** The CIF labels for the atoms in the pattern. Each line of atoms for a given set lists the participating atoms of **one** individual molecule in that set; the corresponding ***symmetry operator*** identifies the symmetry relationship of the participating atoms relative to the first atom in the set.

***H-bond designation*** The letters indicate the symmetry equivalence of the H-bonds within a pattern – thus, all H-bonds labelled *a* are symmetry equivalent, but are not symmetry equivalent to H-bonds labelled *b*.

***Level*** Indicates the number of symmetry-independent H-bonds bonds that compose a given pattern. The graph set analysis was conducted with the *Mercury* default that finds graph sets up to level 2.

***Period*** The size of the repeat unit (number of hydrogen bonds) for ring and chain patterns.

***No. of molecules*** The total number of molecules participating in a pattern.

The sets are most easily visualised by loading the structure CIF into Mercury and selecting “Calculate, Graph Sets…”

**Figure S8: ORTEP plot (50% probability) of *rac*-D-methionyl-D-methionine**

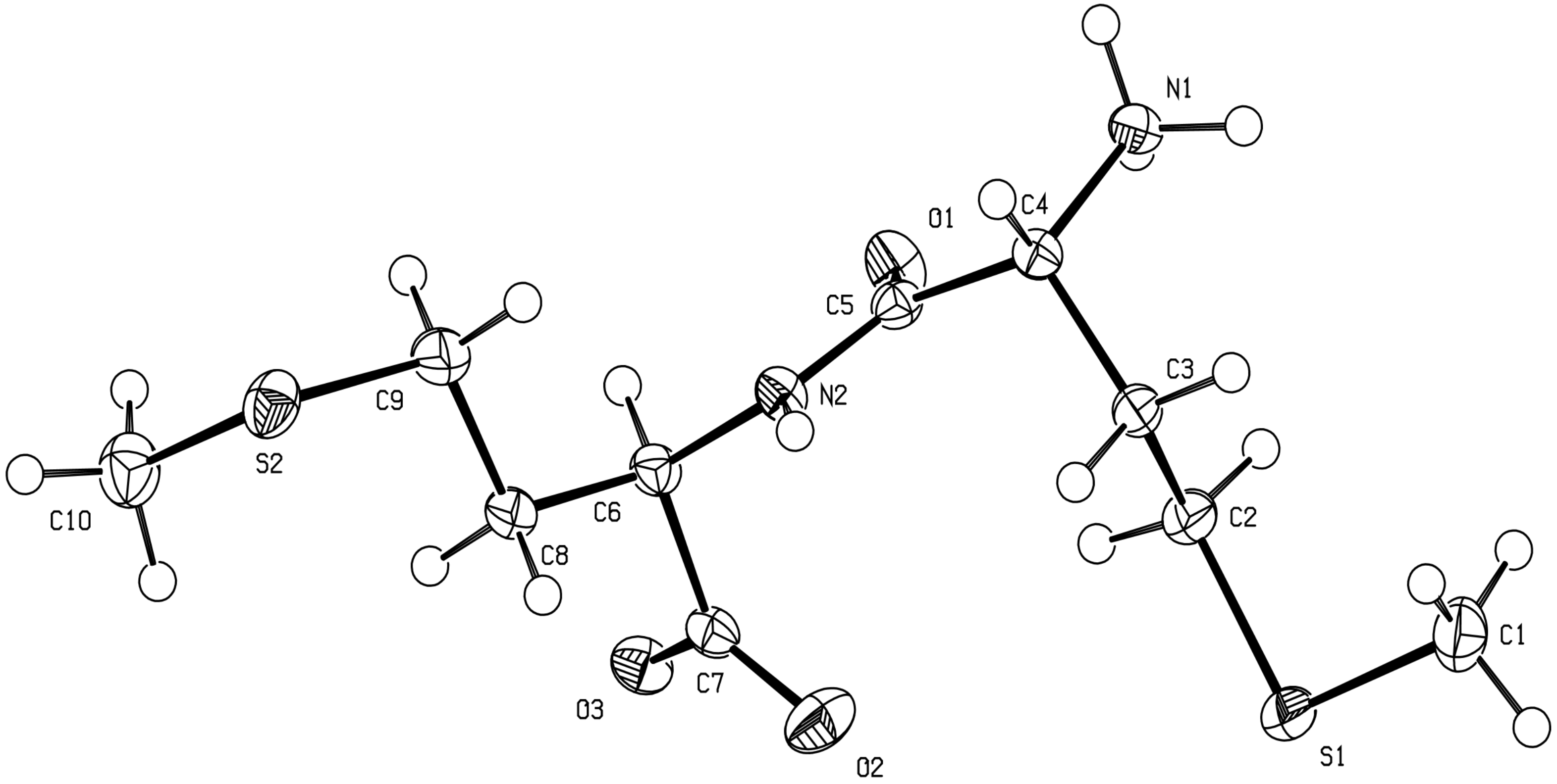

**Figure S9: ORTEP plot (50% probability) of *rac*-D-methionyl-L-methionine**

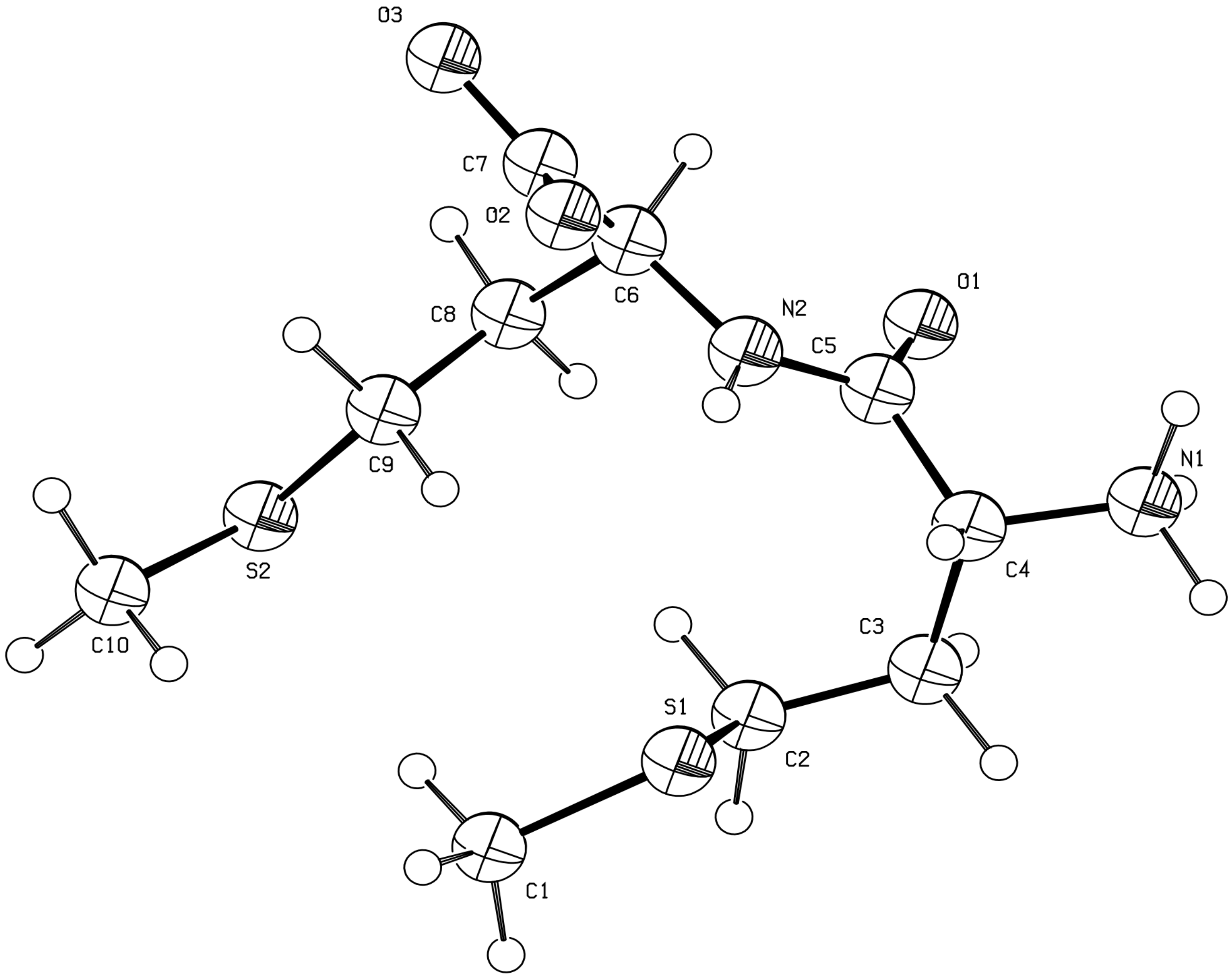

**Table S9.** Calculated transition energies, infrared and Raman intensities with mode descriptions for LL-DD methionylmethionine.
(Average = the average of the factor group components, Range = the difference between the largest and smallest of the factor group components).

| Mode no. | Frequenc / cm-1 | Average / cm-1 | Range / cm-1 | Symmetry | Infrared intensity / ((D/A)**2) amu-1 | Raman intensity / A**4 amu**(-1) | Infrared active? | Raman active? | Description |
|---|---|---|---|---|---|---|---|---|---|
| 1 | 0 | | | Bu | 0.00 | 0.00 | N | N | |
| 2 | 0 | | | Bu | 0.00 | 0.00 | N | N | |
| 3 | 0 | | | Au | 0.00 | 0.00 | N | N | |
| 4 | 19 | | | Ag | 0.00 | 0.00 | N | Y | |
| 5 | 26 | | | Bg | 0.00 | 0.00 | N | Y | |
| 6 | 31 | | | Bg | 0.00 | 0.00 | N | Y | |
| 7 | 34 | | | Au | 0.00 | 0.01 | Y | N | |
| 8 | 39 | | | Bu | 0.07 | 3.17 | Y | N | |
| 9 | 45 | | | Au | 0.03 | 1.22 | Y | N | |
| 10 | 46 | | | Bg | 0.00 | 0.00 | N | Y | |
| 11 | 50 | | | Ag | 0.00 | 0.00 | N | Y | |
| 12 | 52 | | | Ag | 0.00 | 0.00 | N | Y | |
| 13 | 52 | | | Bu | 0.05 | 2.08 | Y | N | |
| 14 | 58 | | | Au | 0.20 | 8.48 | Y | N | |
| 15 | 59 | | | Bg | 0.00 | 0.00 | N | Y | |
| 16 | 65 | | | Ag | 0.00 | 0.00 | N | Y | |
| 17 | 66 | | | Bu | 0.08 | 3.43 | Y | N | |
| 18 | 68 | | | Bg | 0.00 | 0.00 | N | Y | |
| 19 | 69 | | | Bu | 0.13 | 5.71 | Y | N | |
| 20 | 70 | 68 | 4 | Au | 0.03 | 1.18 | Y | N | |
| 21 | 71 | | | Ag | 0.00 | 0.00 | N | Y | |
| 22 | 74 | | | Bg | 0.00 | 0.00 | N | Y | |
| 23 | 80 | | | Bu | 0.25 | 10.88 | Y | N | |
| 24 | 81 | 76 | 10 | Au | 0.02 | 1.06 | Y | N | |
| 25 | 82 | | | Ag | 0.00 | 0.00 | N | Y | |
| 26 | 83 | | | Bg | 0.00 | 0.00 | N | Y | |
| 27 | 84 | | | Au | 0.25 | 10.56 | Y | N | |
| 28 | 84 | 83 | 3 | Bg | 0.00 | 0.00 | N | Y | |
| 29 | 86 | | | Au | 0.17 | 7.29 | Y | N | |
| 30 | 87 | | | Ag | 0.00 | 0.00 | N | Y | |
| 31 | 90 | | | Ag | 0.00 | 0.00 | N | Y | |
| 32 | 92 | 88 | 7 | Bu | 0.00 | 0.19 | Y | N | |
| 33 | 100 | | | Bu | 0.10 | 4.16 | Y | N | |
| 34 | 102 | | | Ag | 0.00 | 0.00 | N | Y | |
| 35 | 106 | | | Au | 0.00 | 0.00 | Y | N | |
| 36 | 108 | 104 | 8 | Bu | 0.42 | 17.87 | Y | N | |
| 37 | 108 | | | Bg | 0.00 | 0.00 | N | Y | |
| 38 | 108 | | | Au | 0.19 | 8.06 | Y | N | |
| 39 | 109 | | | Ag | 0.00 | 0.00 | N | Y | |
| 40 | 110 | 109 | 2 | Bg | 0.00 | 0.00 | N | Y | |
| 41 | 113 | | | Bu | 1.00 | 42.99 | Y | N | |
| 42 | 113 | | | Au | 0.06 | 2.39 | Y | N | |
| 43 | 114 | | | Ag | 0.00 | 0.00 | N | Y | |
| 44 | 116 | 114 | 3 | Bg | 0.00 | 0.00 | N | Y | |
| 45 | 124 | | | Bg | 0.00 | 0.00 | N | Y | |
| 46 | 125 | | | Au | 0.16 | 7.01 | Y | N | |
| 47 | 126 | | | Ag | 0.00 | 0.00 | N | Y | |
| 48 | 128 | 126 | 4 | Bu | 0.59 | 25.30 | Y | N | |
| 49 | 129 | | | Au | 0.05 | 2.34 | Y | N | |
| 50 | 130 | | | Bu | 0.38 | 16.34 | Y | N | |
| 51 | 132 | | | Ag | 0.00 | 0.00 | N | Y | |
| 52 | 134 | 131 | 5 | Bg | 0.00 | 0.00 | N | Y | |
| 53 | 134 | | | Au | 0.63 | 27.28 | Y | N | |
| 54 | 134 | | | Bu | 0.12 | 4.95 | Y | N | |
| 55 | 137 | | | Ag | 0.00 | 0.00 | N | Y | |
| 56 | 139 | 136 | 4 | Bg | 0.00 | 0.00 | N | Y | |
| 57 | 144 | | | Bg | 0.00 | 0.00 | N | Y | |
| 58 | 148 | | | Bu | 0.12 | 5.08 | Y | N | |
| 59 | 149 | | | Ag | 0.00 | 0.00 | N | Y | |
| 60 | 150 | 148 | 6 | Au | 1.19 | 51.37 | Y | N | |
| 61 | 158 | | | Bg | 0.00 | 0.00 | N | Y | |
| 62 | 160 | | | Ag | 0.00 | 0.00 | N | Y | |
| 63 | 162 | | | Bu | 0.44 | 18.86 | Y | N | |
| 64 | 163 | 161 | 5 | Au | 0.09 | 3.80 | Y | N | |
| 65 | 167 | | | Au | 1.62 | 69.65 | Y | N | |
| 66 | 170 | | | Bu | 9.13 | 392.85 | Y | N | |
| 67 | 171 | | | Ag | 0.00 | 0.00 | N | Y | |
| 68 | 171 | 170 | 4 | Bg | 0.00 | 0.00 | N | Y | C9 methyl torsion |
| 69 | 178 | | | Bu | 0.92 | 39.49 | Y | N | |
| 70 | 182 | | | Bg | 0.00 | 0.00 | N | Y | |
| 71 | 184 | | | Au | 0.00 | 0.18 | Y | N | |
| 72 | 187 | 183 | 9 | Au | 0.14 | 5.86 | Y | N | |
| 73 | 188 | | | Ag | 0.00 | 0.00 | N | Y | |
| 74 | 190 | | | Bu | 0.23 | 10.03 | Y | N | |
| 75 | 192 | | | Bg | 0.00 | 0.00 | N | Y | |
| 76 | 192 | 191 | 4 | Ag | 0.00 | 0.00 | N | Y | C1 methyl torsion |
| 77 | 197 | | | Au | 0.00 | 0.09 | Y | N | |
| 78 | 197 | | | Bu | 1.86 | 79.99 | Y | N | |
| 79 | 208 | | | Bg | 0.00 | 0.00 | N | Y | |
| 80 | 208 | 203 | 11 | Ag | 0.00 | 0.00 | N | Y | |
| 81 | 206 | | | Bu | 0.11 | 4.92 | Y | N | |
| 82 | 209 | | | Au | 0.14 | 6.00 | Y | N | |
| 83 | 210 | | | Bg | 0.00 | 0.00 | N | Y | |
| 84 | 211 | 209 | 3 | Ag | 0.00 | 0.00 | N | Y | |
| 85 | 221 | | | Bg | 0.00 | 0.00 | N | Y | |
| 86 | 226 | | | Au | 0.83 | 35.86 | Y | N | |
| 87 | 226 | | | Ag | 0.00 | 0.00 | N | Y | |
| 88 | 227 | 225 | 5 | Bu | 0.39 | 16.61 | Y | N | |
| 89 | 231 | | | Bu | 0.65 | 28.12 | Y | N | |
| 90 | 232 | | | Bg | 0.00 | 0.00 | N | Y | |
| 91 | 240 | | | Au | 2.07 | 88.88 | Y | N | |
| 92 | 244 | 237 | 13 | Ag | 0.00 | 0.00 | N | Y | C1-S1-C2 bend + C2-C3-C4 in-phase bend |
| 93 | 264 | | | Bu | 0.54 | 23.23 | Y | N | |
| 94 | 267 | | | Au | 2.37 | 101.95 | Y | N | |
| 95 | 269 | | | Bg | 0.00 | 0.00 | N | Y | |
| 96 | 271 | 268 | 7 | Ag | 0.00 | 0.00 | N | Y | C6-C7-C8 bend |

| | | | | | | | | | |
|---|---|---|---|---|---|---|---|---|---|
| 97 | 277 | | | Au | 0.16 | 7.00 | Y | N | |
| 98 | 278 | | | Ag | 0.00 | 0.00 | N | Y | |
| 99 | 281 | | | Bg | 0.00 | 0.00 | N | Y | |
| 100 | 282 | 279 | 4 | Bu | 1.12 | 48.33 | Y | N | S1-C2-C3 bend |
| 101 | 303 | | | Au | 0.55 | 23.65 | Y | N | |
| 102 | 304 | | | Ag | 0.00 | 0.00 | N | Y | |
| 103 | 305 | | | Bu | 0.39 | 16.64 | Y | N | |
| 104 | 306 | 304 | 3 | Bg | 0.00 | 0.00 | N | Y | C9-S2-C8 bend |
| 105 | 316 | | | Au | 0.19 | 8.22 | Y | N | |
| 106 | 318 | | | Bg | 0.00 | 0.00 | N | Y | |
| 107 | 318 | | | Ag | 0.00 | 0.00 | N | Y | |
| 108 | 319 | 318 | 2 | Bu | 1.25 | 53.60 | Y | N | C1-S1-C2 bend + C2-C3-C4 out-of-phase bend |
| 109 | 331 | | | Bu | 0.50 | 21.40 | Y | N | |
| 110 | 332 | | | Bg | 0.00 | 0.00 | N | Y | |
| 111 | 340 | | | Au | 0.59 | 25.21 | Y | N | |
| 112 | 341 | 336 | 10 | Ag | 0.00 | 0.00 | N | Y | N2-C4-C5 bend |
| 113 | 361 | | | Bu | 0.67 | 28.85 | Y | N | |
| 114 | 361 | | | Bg | 0.00 | 0.00 | N | Y | |
| 115 | 375 | | | Au | 6.68 | 287.48 | Y | N | |
| 116 | 380 | 369 | 19 | Ag | 0.00 | 0.00 | N | Y | O=C5-C4 bend |
| 117 | 382 | | | Au | 0.55 | 23.47 | Y | N | |
| 118 | 384 | | | Ag | 0.00 | 0.00 | N | Y | |
| 119 | 410 | | | Bg | 0.00 | 0.00 | N | Y | |
| 120 | 410 | 397 | 28 | Bu | 15.34 | 660.11 | Y | N | C3-C4-C5 bend |
| 121 | 429 | | | Bu | 0.19 | 8.28 | Y | N | |
| 122 | 430 | | | Bg | 0.00 | 0.00 | N | Y | |
| 123 | 433 | | | Au | 0.27 | 11.76 | Y | N | |
| 124 | 433 | 431 | 4 | Ag | 0.00 | 0.00 | N | Y | C5-N1-C6 bend |
| 125 | 460 | | | Bg | 0.00 | 0.00 | N | Y | |
| 126 | 460 | | | Bu | 2.30 | 98.92 | Y | N | |
| 127 | 462 | | | Au | 0.96 | 41.15 | Y | N | |
| 128 | 463 | 461 | 3 | Ag | 0.00 | 0.00 | N | Y | NH3 torsion |
| 129 | 504 | | | Bu | 0.16 | 6.81 | Y | N | |
| 130 | 504 | | | Bg | 0.00 | 0.00 | N | Y | |
| 131 | 506 | | | Au | 0.02 | 0.67 | Y | N | |
| 132 | 506 | 505 | 3 | Ag | 0.00 | 0.00 | N | Y | N2-C4-C3 bend |
| 133 | 565 | | | Bu | 3.85 | 165.86 | Y | N | |
| 134 | 565 | | | Bg | 0.00 | 0.00 | N | Y | |
| 135 | 572 | | | Au | 0.98 | 42.31 | Y | N | |
| 136 | 573 | 569 | 9 | Ag | 0.00 | 0.00 | N | Y | N1-C6-C7 bend |
| 137 | 635 | | | Bg | 0.00 | 0.00 | N | Y | |
| 138 | 635 | | | Bu | 0.43 | 18.34 | Y | N | |
| 139 | 638 | | | Au | 2.08 | 89.72 | Y | N | |
| 140 | 639 | 636 | 4 | Ag | 0.00 | 0.00 | N | Y | C4-C5 stretch |
| 141 | 671 | | | Au | 1.91 | 82.34 | Y | N | |
| 142 | 673 | | | Ag | 0.00 | 0.00 | N | Y | |
| 143 | 674 | | | Bg | 0.00 | 0.00 | N | Y | |
| 144 | 674 | 673 | 3 | Bu | 1.30 | 55.90 | Y | N | C9-S2 + S2-C8 in phase stretch |
| 145 | 682 | | | Ag | 0.00 | 0.00 | N | Y | |
| 146 | 683 | | | Bu | 0.49 | 20.89 | Y | N | |
| 147 | 683 | | | Au | 0.12 | 5.04 | Y | N | |
| 148 | 684 | 683 | 2 | Bg | 0.00 | 0.00 | N | Y | C1-S1 + S1-C2 in phase stretch |
| 149 | 701 | | | Au | 0.99 | 42.69 | Y | N | |
| 150 | 703 | | | Ag | 0.00 | 0.00 | N | Y | |
| 151 | 704 | | | Bg | 0.00 | 0.00 | N | Y | |
| 152 | 705 | 703 | 3 | Bu | 0.18 | 7.93 | Y | N | C1-S1 + S1-C2 out-of-phase stretch and C2 + C3 in-phase rock |
| 153 | 710 | | | Bu | 2.12 | 91.38 | Y | N | |
| 154 | 711 | | | Ag | 0.00 | 0.00 | N | Y | |
| 155 | 711 | | | Bg | 0.00 | 0.00 | N | Y | |
| 156 | 711 | 711 | 1 | Au | 0.04 | 1.93 | Y | N | C9-S2 + S2-C8 out-of-phase stretch |
| 157 | 724 | | | Bg | 0.00 | 0.00 | N | Y | |
| 158 | 725 | | | Bu | 0.79 | 33.89 | Y | N | |
| 159 | 725 | | | Au | 2.66 | 114.62 | Y | N | |
| 160 | 726 | 725 | 2 | Ag | 0.00 | 0.00 | N | Y | C7 + C8 out-of-phase methylene rock |
| 161 | 734 | | | Bu | 0.66 | 28.39 | Y | N | |
| 162 | 734 | | | Bg | 0.00 | 0.00 | N | Y | |
| 163 | 735 | | | Au | 2.16 | 92.93 | Y | N | |
| 164 | 736 | 735 | 1 | Ag | 0.00 | 0.00 | N | Y | C2 + C3 out-of-phase methylene rock and C1-S1 + S1-C2 out-of-phase stretch |
| 165 | 738 | | | Ag | 0.00 | 0.00 | N | Y | |
| 166 | 739 | | | Au | 0.53 | 22.79 | Y | N | |
| 167 | 739 | | | Bu | 0.20 | 8.65 | Y | N | |
| 168 | 739 | 739 | 1 | Bg | 0.00 | 0.00 | N | Y | CO2- out-of-plane bend |
| 169 | 762 | | | Ag | 0.00 | 0.00 | N | Y | |
| 170 | 762 | | | Au | 0.00 | 0.02 | Y | N | |
| 171 | 764 | | | Bg | 0.00 | 0.00 | N | Y | |
| 172 | 764 | 763 | 2 | Bu | 0.96 | 41.33 | Y | N | Carboxylate O-C10-O in-plane bend |
| 173 | 774 | | | Bu | 1.13 | 48.83 | Y | N | |
| 174 | 775 | | | Bg | 0.00 | 0.00 | N | Y | |
| 175 | 776 | | | Au | 8.57 | 368.98 | Y | N | |
| 176 | 780 | 776 | 5 | Ag | 0.00 | 0.00 | N | Y | N1-H out-of-plane bend |
| 177 | 794 | | | Au | 0.15 | 6.26 | Y | N | |
| 178 | 794 | | | Ag | 0.00 | 0.00 | N | Y | |
| 179 | 796 | | | Bu | 0.03 | 1.34 | Y | N | |
| 180 | 796 | 795 | 2 | Bg | 0.00 | 0.00 | N | Y | C2-C3-C4 bend |
| 181 | 845 | | | Ag | 0.00 | 0.00 | N | Y | |
| 182 | 845 | | | Au | 0.01 | 0.42 | Y | N | |
| 183 | 845 | | | Bg | 0.00 | 0.00 | N | Y | |
| 184 | 846 | 845 | 1 | Bu | 0.06 | 2.60 | Y | N | C3-C4 stretch |
| 185 | 876 | | | Bu | 0.58 | 24.91 | Y | N | |
| 186 | 876 | | | Bg | 0.00 | 0.00 | N | Y | |
| 187 | 878 | | | Au | 0.05 | 2.34 | Y | N | |
| 188 | 878 | 877 | 2 | Ag | 0.00 | 0.00 | N | Y | C7 + C8 in-phase methylene rock |
| 189 | 898 | | | Au | 0.71 | 30.41 | Y | N | |
| 190 | 899 | | | Ag | 0.00 | 0.00 | N | Y | |
| 191 | 901 | | | Bg | 0.00 | 0.00 | N | Y | |
| 192 | 901 | 900 | 3 | Bu | 2.47 | 106.15 | Y | N | C7 + C8 in-phase methylene twist |
| 193 | 909 | | | Bu | 2.13 | 91.81 | Y | N | |
| 194 | 910 | | | Bg | 0.00 | 0.00 | N | Y | |
| 195 | 911 | | | Au | 0.25 | 10.71 | Y | N | |
| 196 | 912 | 911 | 3 | Ag | 0.00 | 0.00 | N | Y | C6-C10 stretch |
| 197 | 933 | | | Bu | 1.78 | 76.46 | Y | N | |

| | | | | | | | | | |
|---|---|---|---|---|---|---|---|---|---|
| 198 | 933 | | | Bg | 0.00 | 0.00 | N | Y | |
| 199 | 936 | | | Ag | 0.00 | 0.00 | N | Y | |
| 200 | 936 | 935 | 3 | Au | 1.65 | 70.86 | Y | N | C9 methyl rock |
| 201 | 939 | | | Au | 0.11 | 4.77 | Y | N | |
| 202 | 940 | | | Bg | 0.00 | 0.00 | N | Y | |
| 203 | 940 | | | Bu | 0.38 | 16.14 | Y | N | |
| 204 | 940 | 940 | 1 | Ag | 0.00 | 0.00 | N | Y | C9 methyl rock |
| 205 | 957 | | | Bu | 0.53 | 22.69 | Y | N | |
| 206 | 957 | | | Bg | 0.00 | 0.00 | N | Y | |
| 207 | 957 | | | Au | 0.34 | 14.48 | Y | N | |
| 208 | 958 | 957 | 1 | Ag | 0.00 | 0.00 | N | Y | C1 methyl rock |
| 209 | 959 | | | Bu | 0.25 | 10.88 | Y | N | |
| 210 | 959 | | | Ag | 0.00 | 0.00 | N | Y | |
| 211 | 959 | | | Au | 0.00 | 0.06 | Y | N | |
| 212 | 960 | 959 | 0 | Bg | 0.00 | 0.00 | N | Y | C1 methyl rock |
| 213 | 989 | | | Ag | 0.00 | 0.00 | N | Y | |
| 214 | 990 | | | Au | 0.12 | 5.14 | Y | N | |
| 215 | 991 | | | Bg | 0.00 | 0.00 | N | Y | |
| 216 | 992 | 990 | 3 | Bu | 3.82 | 164.61 | Y | N | C4-NH3 stretch |
| 217 | 1011 | | | Au | 0.86 | 37.18 | Y | N | |
| 218 | 1011 | | | Ag | 0.00 | 0.00 | N | Y | |
| 219 | 1013 | | | Bu | 0.89 | 38.48 | Y | N | |
| 220 | 1014 | 1012 | 3 | Bg | 0.00 | 0.00 | N | Y | NH3 rock |
| 221 | 1015 | | | Au | 0.76 | 32.88 | Y | N | |
| 222 | 1016 | | | Bu | 1.73 | 74.46 | Y | N | |
| 223 | 1018 | | | Bg | 0.00 | 0.00 | N | Y | |
| 224 | 1019 | 1017 | 4 | Ag | 0.00 | 0.00 | N | Y | C7-C8 stretch |
| 225 | 1039 | | | Au | 1.61 | 69.40 | Y | N | |
| 226 | 1041 | | | Bu | 3.30 | 142.07 | Y | N | |
| 227 | 1042 | | | Bg | 0.00 | 0.00 | N | Y | |
| 228 | 1042 | 1041 | 3 | Ag | 0.00 | 0.00 | N | Y | C6-C7 stretch |
| 229 | 1048 | | | Bu | 0.07 | 3.17 | Y | N | |
| 230 | 1048 | | | Bg | 0.00 | 0.00 | N | Y | |
| 231 | 1049 | | | Ag | 0.00 | 0.00 | N | Y | |
| 232 | 1050 | 1049 | 2 | Au | 0.56 | 23.99 | Y | N | NH3 rock |
| 233 | 1061 | | | Ag | 0.00 | 0.00 | N | Y | |
| 234 | 1061 | | | Au | 0.92 | 39.69 | Y | N | |
| 235 | 1063 | | | Bg | 0.00 | 0.00 | N | Y | |
| 236 | 1063 | 1062 | 2 | Bu | 0.14 | 5.88 | Y | N | C3-C4 stretch |
| 237 | 1093 | | | Bg | 0.00 | 0.00 | N | Y | |
| 238 | 1093 | | | Bu | 3.47 | 149.48 | Y | N | |
| 239 | 1094 | | | Au | 0.07 | 2.91 | Y | N | |
| 240 | 1094 | 1094 | 2 | Ag | 0.00 | 0.00 | N | Y | C7 + C8 out-of-phase methylene twist |
| 241 | 1129 | | | Bg | 0.00 | 0.00 | N | Y | |
| 242 | 1129 | | | Bu | 0.45 | 19.19 | Y | N | |
| 243 | 1133 | | | Ag | 0.00 | 0.00 | N | Y | |
| 244 | 1134 | 1131 | 5 | Au | 1.38 | 59.20 | Y | N | C2-C3 stretch |
| 245 | 1143 | | | Bu | 0.17 | 7.34 | Y | N | |
| 246 | 1144 | | | Au | 0.16 | 7.07 | Y | N | |
| 247 | 1145 | | | Bg | 0.00 | 0.00 | N | Y | |
| 248 | 1146 | 1145 | 3 | Ag | 0.00 | 0.00 | N | Y | C6-H bend |
| 249 | 1169 | | | Bg | 0.00 | 0.00 | N | Y | |
| 250 | 1170 | | | Bu | 3.91 | 168.21 | Y | N | |
| 251 | 1170 | | | Au | 0.08 | 3.51 | Y | N | |
| 252 | 1170 | 1170 | 1 | Ag | 0.00 | 0.00 | N | Y | C2 + C3 out-of-phase methylene twist |
| 253 | 1198 | | | Ag | 0.00 | 0.00 | N | Y | |
| 254 | 1198 | | | Bg | 0.00 | 0.00 | N | Y | |
| 255 | 1200 | | | Au | 0.06 | 2.74 | Y | N | |
| 256 | 1200 | 1199 | 2 | Bu | 1.39 | 59.93 | Y | N | C8 methylene wag |
| 257 | 1218 | | | Ag | 0.00 | 0.00 | N | Y | |
| 258 | 1219 | | | Au | 0.17 | 7.43 | Y | N | |
| 259 | 1221 | | | Bu | 6.09 | 261.89 | Y | N | |
| 260 | 1221 | 1220 | 2 | Bg | 0.00 | 0.00 | N | Y | C2 methylene wag |
| 261 | 1232 | | | Bg | 0.00 | 0.00 | N | Y | |
| 262 | 1233 | | | Bu | 11.73 | 504.68 | Y | N | |
| 263 | 1233 | | | Ag | 0.00 | 0.00 | N | Y | |
| 264 | 1233 | 1233 | 2 | Au | 1.24 | 53.24 | Y | N | C2-C3 stretch |
| 265 | 1236 | | | Bu | 6.60 | 284.17 | Y | N | |
| 266 | 1236 | | | Au | 0.12 | 5.28 | Y | N | |
| 267 | 1237 | | | Ag | 0.00 | 0.00 | N | Y | |
| 268 | 1238 | 1237 | 2 | Bg | 0.00 | 0.00 | N | Y | C8 methylene twist |
| 269 | 1258 | | | Bg | 0.00 | 0.00 | N | Y | |
| 270 | 1258 | | | Bu | 7.93 | 341.13 | Y | N | |
| 271 | 1260 | | | Au | 0.98 | 41.99 | Y | N | |
| 272 | 1260 | 1259 | 3 | Ag | 0.00 | 0.00 | N | Y | C2 methylene wag |
| 273 | 1280 | | | Bg | 0.00 | 0.00 | N | Y | |
| 274 | 1280 | | | Ag | 0.00 | 0.00 | N | Y | |
| 275 | 1280 | | | Bu | 7.08 | 304.72 | Y | N | |
| 276 | 1282 | 1280 | 2 | Au | 0.16 | 7.04 | Y | N | C7 + C8 in-phase methylene twist |
| 277 | 1286 | | | Au | 1.17 | 50.46 | Y | N | |
| 278 | 1286 | | | Ag | 0.00 | 0.00 | N | Y | |
| 279 | 1287 | | | Bu | 2.38 | 102.37 | Y | N | |
| 280 | 1287 | 1286 | 2 | Bg | 0.00 | 0.00 | N | Y | C2 + C3 in-phase methylene twist |
| 281 | 1293 | | | Au | 4.99 | 214.54 | Y | N | |
| 282 | 1295 | | | Ag | 0.00 | 0.00 | N | Y | |
| 283 | 1296 | | | Bu | 4.67 | 201.06 | Y | N | |
| 284 | 1296 | 1295 | 3 | Bg | 0.00 | 0.00 | N | Y | N1-C5 stretch |
| 285 | 1303 | | | Au | 1.03 | 44.31 | Y | N | |
| 286 | 1303 | | | Bu | 1.04 | 44.58 | Y | N | |
| 287 | 1305 | | | Bg | 0.00 | 0.00 | N | Y | |
| 288 | 1305 | 1304 | 2 | Ag | 0.00 | 0.00 | N | Y | C9-H sym methyl bend |
| 289 | 1305 | | | Bu | 0.16 | 6.92 | Y | N | |
| 290 | 1305 | | | Au | 0.00 | 0.03 | Y | N | |
| 291 | 1305 | | | Bg | 0.00 | 0.00 | N | Y | |
| 292 | 1306 | 1305 | 1 | Ag | 0.00 | 0.00 | N | Y | C7 methylene wag |
| 293 | 1312 | | | Au | 0.03 | 1.16 | Y | N | |
| 294 | 1313 | | | Bu | 0.37 | 15.99 | Y | N | |
| 295 | 1315 | | | Ag | 0.00 | 0.00 | N | Y | |
| 296 | 1316 | 1314 | 3 | Bg | 0.00 | 0.00 | N | Y | C1-H sym methyl bend |
| 297 | 1333 | | | Au | 6.43 | 276.71 | Y | N | |
| 298 | 1334 | | | Ag | 0.00 | 0.00 | N | Y | |

| | | | | | | | | | |
|---|---|---|---|---|---|---|---|---|---|
| 299 | 1335 | | | Bu | 0.52 | 22.52 | Y | N | |
| 300 | 1335 | 1335 | 2 | Bg | 0.00 | 0.00 | N | Y | C3 methylene wag |
| 301 | 1340 | | | Bg | 0.00 | 0.00 | N | Y | |
| 302 | 1341 | | | Bu | 24.21 | 1042.06 | Y | N | |
| 303 | 1345 | | | Au | 6.88 | 296.08 | Y | N | |
| 304 | 1346 | 1343 | 6 | Ag | 0.00 | 0.00 | N | Y | Sym O-C-O stretch |
| 305 | 1368 | | | Ag | 0.00 | 0.00 | N | Y | |
| 306 | 1368 | | | Au | 0.10 | 4.31 | Y | N | |
| 307 | 1369 | | | Bu | 3.06 | 131.77 | Y | N | |
| 308 | 1369 | 1369 | 1 | Bg | 0.00 | 0.00 | N | Y | C4-H bend |
| 309 | 1395 | | | Bu | 0.25 | 10.60 | Y | N | |
| 310 | 1396 | | | Au | 0.31 | 13.22 | Y | N | |
| 311 | 1404 | | | Bg | 0.00 | 0.00 | N | Y | |
| 312 | 1404 | 1400 | 9 | Ag | 0.00 | 0.00 | N | Y | C8 methylene scissors |
| 313 | 1405 | | | Au | 1.91 | 82.11 | Y | N | |
| 314 | 1406 | | | Bu | 4.34 | 186.56 | Y | N | |
| 315 | 1406 | | | Bg | 0.00 | 0.00 | N | Y | |
| 316 | 1406 | 1406 | 1 | Ag | 0.00 | 0.00 | N | Y | C9-H asym methyl bend |
| 317 | 1409 | | | Ag | 0.00 | 0.00 | N | Y | |
| 318 | 1409 | | | Bg | 0.00 | 0.00 | N | Y | |
| 319 | 1410 | | | Au | 0.81 | 35.05 | Y | N | |
| 320 | 1411 | 1409 | 2 | Bu | 0.75 | 32.14 | Y | N | C9-H asym methyl bend and C8 methylene scissors |
| 321 | 1414 | | | Ag | 0.00 | 0.00 | N | Y | |
| 322 | 1415 | | | Bg | 0.00 | 0.00 | N | Y | |
| 323 | 1416 | | | Au | 5.84 | 251.19 | Y | N | |
| 324 | 1418 | 1416 | 4 | Bu | 2.32 | 99.97 | Y | N | C1-H asym methyl bend and C2 methylene scissors |
| 325 | 1418 | | | Bu | 0.22 | 9.52 | Y | N | |
| 326 | 1418 | | | Au | 0.15 | 6.36 | Y | N | |
| 327 | 1418 | | | Ag | 0.00 | 0.00 | N | Y | |
| 328 | 1419 | 1418 | 1 | Bg | 0.00 | 0.00 | N | Y | C1-H asym methyl bend |
| 329 | 1430 | | | Au | 0.11 | 4.93 | Y | N | |
| 330 | 1430 | | | Ag | 0.00 | 0.00 | N | Y | |
| 331 | 1430 | | | Bg | 0.00 | 0.00 | N | Y | |
| 332 | 1431 | 1430 | 1 | Bu | 1.87 | 80.48 | Y | N | C3 + C6 in-phase methylene scissors |
| 333 | 1431 | | | Ag | 0.00 | 0.00 | N | Y | |
| 334 | 1432 | | | Bu | 1.68 | 72.35 | Y | N | |
| 335 | 1432 | | | Au | 0.00 | 0.04 | Y | N | |
| 336 | 1433 | 1432 | 1 | Bg | 0.00 | 0.00 | N | Y | C3 + C6 out-of-phase methylene scissors |
| 337 | 1433 | | | Au | 0.28 | 11.94 | Y | N | |
| 338 | 1434 | | | Bu | 1.23 | 53.13 | Y | N | |
| 339 | 1439 | | | Bg | 0.00 | 0.00 | N | Y | |
| 340 | 1439 | 1436 | 7 | Ag | 0.00 | 0.00 | N | Y | C1 + C9-H asym methyl bend |
| 341 | 1510 | | | Bu | 57.04 | 2454.65 | Y | N | |
| 342 | 1510 | | | Bg | 0.00 | 0.00 | N | Y | |
| 343 | 1519 | | | Au | 0.72 | 31.14 | Y | N | |
| 344 | 1519 | 1515 | 9 | Ag | 0.00 | 0.00 | N | Y | NH3 sym bend |
| 345 | 1533 | | | Bg | 0.00 | 0.00 | N | Y | |
| 346 | 1534 | | | Bu | 9.85 | 423.92 | Y | N | |
| 347 | 1536 | | | Ag | 0.00 | 0.00 | N | Y | |
| 348 | 1537 | 1535 | 3 | Au | 2.62 | 112.74 | Y | N | N1-H in-plane bend |
| 349 | 1547 | | | Bg | 0.00 | 0.00 | N | Y | |
| 350 | 1548 | | | Bu | 85.88 | 3695.95 | Y | N | |
| 351 | 1551 | | | Au | 19.74 | 849.37 | Y | N | |
| 352 | 1555 | 1550 | 8 | Ag | 0.00 | 0.00 | N | Y | Asym O-C-O stretch |
| 353 | 1614 | | | Bg | 0.00 | 0.00 | N | Y | |
| 354 | 1614 | | | Bu | 11.16 | 480.11 | Y | N | |
| 355 | 1620 | | | Ag | 0.00 | 0.00 | N | Y | |
| 356 | 1621 | 1617 | 7 | Au | 9.53 | 410.11 | Y | N | NH3 asym bend |
| 357 | 1624 | | | Au | 1.43 | 61.63 | Y | N | |
| 358 | 1625 | | | Ag | 0.00 | 0.00 | N | Y | |
| 359 | 1629 | | | Bg | 0.00 | 0.00 | N | Y | |
| 360 | 1629 | 1627 | 5 | Bu | 12.24 | 526.87 | Y | N | NH3 asym bend and C5=O stretch |
| 361 | 1645 | | | Au | 2.94 | 126.36 | Y | N | |
| 362 | 1645 | | | Bg | 0.00 | 0.00 | N | Y | |
| 363 | 1645 | | | Bu | 9.65 | 415.39 | Y | N | |
| 364 | 1645 | 1645 | 1 | Ag | 0.00 | 0.00 | N | Y | C5=O stretch and NH3 asym bend |
| 365 | 2533 | | | Bg | 0.00 | 0.00 | N | Y | |
| 366 | 2534 | | | Bu | 132.27 | 5692.49 | Y | N | |
| 367 | 2549 | | | Au | 119.60 | 5146.96 | Y | N | |
| 368 | 2564 | 2545 | 31 | Ag | 0.00 | 0.00 | N | Y | H2N-H H-bonded to carboxylate stretch |
| 369 | 2933 | | | Au | 0.10 | 4.15 | Y | N | |
| 370 | 2933 | | | Bu | 3.84 | 165.42 | Y | N | |
| 371 | 2934 | | | Bg | 0.00 | 0.00 | N | Y | |
| 372 | 2934 | 2933 | 1 | Ag | 0.00 | 0.00 | N | Y | C8 sym methylene stretch |
| 373 | 2946 | | | Bu | 1.64 | 70.65 | Y | N | |
| 374 | 2946 | | | Bg | 0.00 | 0.00 | N | Y | |
| 375 | 2947 | | | Au | 0.04 | 1.70 | Y | N | |
| 376 | 2947 | 2947 | 0 | Ag | 0.00 | 0.00 | N | Y | In-phase C1 sym methyl stretch + C2 sym methylene stretch |
| 377 | 2950 | | | Au | 0.38 | 16.19 | Y | N | |
| 378 | 2950 | | | Bu | 2.49 | 107.22 | Y | N | |
| 379 | 2950 | | | Bg | 0.00 | 0.00 | N | Y | |
| 380 | 2950 | 2950 | 0 | Ag | 0.00 | 0.00 | N | Y | Out-of-phase C1 sym methyl stretch + C2 sym methylene stretch |
| 381 | 2968 | | | Bu | 1.72 | 74.16 | Y | N | |
| 382 | 2968 | | | Au | 0.09 | 3.85 | Y | N | |
| 383 | 2968 | | | Bg | 0.00 | 0.00 | N | Y | |
| 384 | 2968 | 2968 | 0 | Ag | 0.00 | 0.00 | N | Y | In-phase C3 sym methylene stretch + C4-H stretch |
| 385 | 2971 | | | Bu | 1.77 | 76.34 | Y | N | |
| 386 | 2971 | | | Bg | 0.00 | 0.00 | N | Y | |
| 387 | 2971 | | | Au | 0.02 | 0.79 | Y | N | |
| 388 | 2971 | 2971 | 0 | Ag | 0.00 | 0.00 | N | Y | C9 sym methyl stretch |
| 389 | 2978 | | | Au | 1.26 | 54.19 | Y | N | |
| 390 | 2978 | | | Bu | 3.73 | 160.47 | Y | N | |
| 391 | 2978 | | | Bg | 0.00 | 0.00 | N | Y | |
| 392 | 2978 | 2978 | 0 | Ag | 0.00 | 0.00 | N | Y | Out-of-phase C7 sym methylene stretch + C6-H stretch |
| 393 | 2990 | | | Bu | 1.33 | 57.09 | Y | N | |
| 394 | 2990 | | | Au | 0.11 | 4.76 | Y | N | |
| 395 | 2990 | | | Bg | 0.00 | 0.00 | N | Y | |
| 396 | 2990 | 2990 | 0 | Ag | 0.00 | 0.00 | N | Y | Out-of-phase C3 sym methylene stretch + C4-H stretch |
| 397 | 3001 | | | Au | 0.55 | 23.55 | Y | N | |
| 398 | 3001 | | | Ag | 0.00 | 0.00 | N | Y | |
| 399 | 3001 | | | Bu | 0.71 | 30.43 | Y | N | |

| | | | | | | | | | | |
|---|---|---|---|---|---|---|---|---|---|---|
| 400 | 3001 | 3001 | 0 | Bg | 0.00 | 0.00 | N | Y | C2 asym methylene stretch | |
| 401 | 3012 | | | Bu | 0.04 | 1.66 | Y | N | | |
| 402 | 3012 | | | Au | 0.06 | 2.67 | Y | N | | |
| 403 | 3014 | | | Bg | 0.00 | 0.00 | N | Y | | |
| 404 | 3014 | 3013 | 2 | Ag | 0.00 | 0.00 | N | Y | Out-of-phase C7 + C8 asym methylene stretch | |
| 405 | 3031 | | | Au | 0.46 | 19.69 | Y | N | | |
| 406 | 3031 | | | Bu | 2.40 | 103.26 | Y | N | | |
| 407 | 3032 | | | Ag | 0.00 | 0.00 | N | Y | | |
| 408 | 3032 | 3031 | 1 | Bg | 0.00 | 0.00 | N | Y | In-phase C7 + C8 asym methylene stretch | |
| 409 | 3035 | | | Au | 0.14 | 6.24 | Y | N | | |
| 410 | 3035 | | | Bg | 0.00 | 0.00 | N | Y | | |
| 411 | 3035 | | | Ag | 0.00 | 0.00 | N | Y | | |
| 412 | 3035 | 3035 | 0 | Bu | 3.68 | 158.21 | Y | N | C3 asym methylene stretch | ???? |
| 413 | 3037 | | | Ag | 0.00 | 0.00 | N | Y | | |
| 414 | 3037 | | | Bu | 0.88 | 37.91 | Y | N | | |
| 415 | 3037 | | | Au | 0.02 | 0.65 | Y | N | | |
| 416 | 3037 | 3037 | 0 | Bg | 0.00 | 0.00 | N | Y | C3 asym methylene stretch | |
| 417 | 3044 | | | Bu | 0.04 | 1.71 | Y | N | | |
| 418 | 3044 | | | Au | 0.02 | 0.79 | Y | N | | |
| 419 | 3044 | | | Bg | 0.00 | 0.00 | N | Y | | |
| 420 | 3044 | 3044 | 0 | Ag | 0.00 | 0.00 | N | Y | C1 asym methyl stretch | |
| 421 | 3054 | | | Bg | 0.00 | 0.00 | N | Y | | |
| 422 | 3054 | | | Ag | 0.00 | 0.00 | N | Y | | |
| 423 | 3054 | | | Bu | 0.39 | 16.91 | Y | N | | |
| 424 | 3054 | 3054 | 0 | Au | 0.01 | 0.34 | Y | N | C1 asym methyl stretch | |
| 425 | 3060 | | | Bg | 0.00 | 0.00 | N | Y | | |
| 426 | 3060 | | | Ag | 0.00 | 0.00 | N | Y | | |
| 427 | 3060 | | | Au | 0.72 | 30.84 | Y | N | | |
| 428 | 3061 | 3060 | 0 | Bu | 0.08 | 3.53 | Y | N | C9 asym methyl stretch | |
| 429 | 3074 | | | Bg | 0.00 | 0.00 | N | Y | | |
| 430 | 3074 | | | Ag | 0.00 | 0.00 | N | Y | | |
| 431 | 3074 | | | Au | 0.14 | 6.08 | Y | N | | |
| 432 | 3074 | 3074 | 0 | Bu | 0.44 | 19.07 | Y | N | C9 asym methyl stretch | |
| 433 | 3100 | | | Bg | 0.00 | 0.00 | N | Y | | |
| 434 | 3100 | | | Bu | 59.64 | 2566.62 | Y | N | | |
| 435 | 3100 | | | Au | 2.93 | 125.88 | Y | N | | |
| 436 | 3101 | 3100 | 1 | Ag | 0.00 | 0.00 | N | Y | NH3 asym stretch | |
| 437 | 3150 | | | Bg | 0.00 | 0.00 | N | Y | | |
| 438 | 3151 | | | Bu | 110.46 | 4753.65 | Y | N | | |
| 439 | 3152 | | | Ag | 0.00 | 0.00 | N | Y | | |
| 440 | 3152 | 3151 | 1 | Au | 2.84 | 122.29 | Y | N | N1-H stretch | |
| 441 | 3185 | | | Bu | 38.01 | 1635.64 | Y | N | | |
| 442 | 3185 | | | Bg | 0.00 | 0.00 | N | Y | | |
| 443 | 3187 | | | Au | 28.21 | 1214.07 | Y | N | | |
| 444 | 3190 | 3187 | 5 | Ag | 0.00 | 0.00 | N | Y | NH3 asym stretch | |

**Table S10.** Calculated transition energies, infrared and Raman intensities with mode descriptions for DL-LD methionylmethionine.
(Average = the average of the factor group components, Range = the difference between the largest and smallest of the factor group components).

| Mode no. | Frequency / cm-1 | Average / cm-1 | Range / cm-1 | Symmetry | Infrared intensity / / ((D/A)**2) amu-1 | Infrared active? | Raman active? | Description |
|---|---|---|---|---|---|---|---|---|
| 1 | 0 | | | Bu | 0.00 | N | N | |
| 2 | 0 | | | Au | 0.00 | N | N | |
| 3 | 0 | | | Bu | 0.00 | N | N | |
| 4 | 12 | | | Ag | 0.00 | N | Y | |
| 5 | 28 | | | Au | 0.00 | Y | N | |
| 6 | 31 | | | Bg | 0.00 | N | Y | |
| 7 | 32 | | | Ag | 0.00 | N | Y | |
| 8 | 36 | | | Ag | 0.00 | N | Y | |
| 9 | 38 | | | Bu | 0.00 | Y | N | |
| 10 | 39 | | | Bg | 0.00 | N | Y | |
| 11 | 40 | | | Au | 0.01 | Y | N | |
| 12 | 48 | | | Bu | 0.01 | Y | N | |
| 13 | 50 | | | Bg | 0.00 | N | Y | |
| 14 | 55 | | | Au | 0.02 | Y | N | |
| 15 | 60 | | | Bu | 0.00 | Y | N | |
| 16 | 61 | | | Bg | 0.00 | N | Y | |
| 17 | 61 | | | Ag | 0.00 | N | Y | |
| 18 | 63 | | | Au | 0.01 | Y | N | |
| 19 | 67 | | | Bu | 0.23 | Y | N | |
| 20 | 68 | 65 | 6 | Au | 0.15 | Y | N | |
| 21 | 68 | | | Ag | 0.00 | N | Y | |
| 22 | 69 | | | Bg | 0.00 | N | Y | |
| 23 | 71 | | | Bg | 0.00 | N | Y | |
| 24 | 71 | 70 | 3 | Bg | 0.00 | N | Y | |
| 25 | 74 | | | Bu | 0.03 | Y | N | |
| 26 | 78 | | | Ag | 0.00 | N | Y | |
| 27 | 79 | | | Au | 0.00 | Y | N | |
| 28 | 79 | 77 | 6 | Bu | 1.21 | Y | N | |
| 29 | 87 | | | Ag | 0.00 | N | Y | |
| 30 | 88 | | | Bu | 0.31 | Y | N | |
| 31 | 88 | | | Ag | 0.00 | N | Y | |
| 32 | 95 | 90 | 8 | Au | 0.11 | Y | N | |
| 33 | 95 | | | Bg | 0.00 | N | Y | |
| 34 | 96 | | | Ag | 0.00 | N | Y | |
| 35 | 96 | | | Bu | 0.37 | Y | N | |
| 36 | 97 | 96 | 2 | Ag | 0.00 | N | Y | |
| 37 | 101 | | | Bg | 0.00 | N | Y | |
| 38 | 102 | | | Au | 0.00 | Y | N | |
| 39 | 102 | | | Ag | 0.00 | N | Y | |
| 40 | 104 | 102 | 3 | Bu | 1.12 | Y | N | |
| 41 | 105 | | | Au | 0.00 | Y | N | |
| 42 | 106 | | | Bg | 0.00 | N | Y | |
| 43 | 114 | | | Bg | 0.00 | N | Y | |
| 44 | 114 | 110 | 9 | Bg | 0.00 | N | Y | |
| 45 | 115 | | | Ag | 0.00 | N | Y | |
| 46 | 115 | | | Au | 0.04 | Y | N | |
| 47 | 115 | | | Bu | 0.34 | Y | N | |
| 48 | 118 | 116 | 3 | Bu | 0.06 | Y | N | |
| 49 | 119 | | | Ag | 0.00 | N | Y | |
| 50 | 120 | | | Au | 0.03 | Y | N | |
| 51 | 124 | | | Ag | 0.00 | N | Y | |
| 52 | 125 | 122 | 6 | Au | 0.01 | Y | N | |
| 53 | 127 | | | Bu | 1.08 | Y | N | |
| 54 | 127 | | | Bg | 0.00 | N | Y | |
| 55 | 129 | | | Au | 0.00 | Y | N | |
| 56 | 132 | 129 | 5 | Ag | 0.00 | N | Y | |
| 57 | 133 | | | Bu | 0.31 | Y | N | |
| 58 | 136 | | | Bg | 0.00 | N | Y | |
| 59 | 137 | | | Au | 0.18 | Y | N | |
| 60 | 139 | 136 | 5 | Bu | 1.41 | Y | N | |
| 61 | 140 | | | Bg | 0.00 | N | Y | |
| 62 | 142 | | | Ag | 0.00 | N | Y | |
| 63 | 143 | | | Bg | 0.00 | N | Y | |
| 64 | 159 | 146 | 18 | Au | 0.27 | Y | N | |
| 65 | 163 | | | Ag | 0.00 | N | Y | |
| 66 | 166 | | | Au | 0.08 | Y | N | |
| 67 | 167 | | | Bu | 1.04 | Y | N | |
| 68 | 168 | 166 | 5 | Bg | 0.00 | N | Y | |
| 69 | 169 | | | Ag | 0.00 | N | Y | |
| 70 | 176 | | | Bu | 2.02 | Y | N | |
| 71 | 178 | | | Au | 0.16 | Y | N | |
| 72 | 179 | 175 | 10 | Bg | 0.00 | N | Y | |
| 73 | 191 | | | Bu | 0.29 | Y | N | |
| 74 | 191 | | | Bg | 0.00 | N | Y | |
| 75 | 192 | | | Au | 0.15 | Y | N | |
| 76 | 193 | 192 | 2 | Ag | 0.00 | N | Y | C1 + C9 out-of-phase methyl torsion |
| 77 | 193 | | | Au | 0.04 | Y | N | |
| 78 | 196 | | | Ag | 0.00 | N | Y | |
| 79 | 203 | | | Bg | 0.00 | N | Y | |
| 80 | 204 | 199 | 10 | Bu | 0.15 | Y | N | C1 + C9 in-phase methyl torsion |
| 81 | 209 | | | Au | 4.29 | Y | N | |
| 82 | 214 | | | Bg | 0.00 | N | Y | |
| 83 | 215 | | | Au | 0.03 | Y | N | |
| 84 | 215 | 214 | 12 | Bu | 1.00 | Y | N | |
| 85 | 217 | | | Ag | 0.00 | N | Y | |
| 86 | 221 | | | Ag | 0.00 | N | Y | |
| 87 | 223 | | | Bg | 0.00 | N | Y | |
| 88 | 225 | 222 | 9 | Bu | 3.46 | Y | N | C1-S1-C2 bend |

| | | | | | | | | |
|---|---|---|---|---|---|---|---|---|
| 89 | 227 | | | Bg | 0.00 | N | Y | |
| 90 | 231 | | | Au | 0.84 | Y | N | |
| 91 | 244 | | | Au | 0.21 | Y | N | |
| 92 | 244 | 236 | 18 | Bg | 0.00 | N | Y | C5-N1-C6 bend |
| 93 | 247 | | | Bu | 2.79 | Y | N | |
| 94 | 247 | | | Ag | 0.00 | N | Y | |
| 95 | 273 | | | Ag | 0.00 | N | Y | |
| 96 | 273 | 260 | 26 | Bu | 4.73 | Y | N | N1-C6-C7 bend |
| 97 | 282 | | | Bu | 6.28 | Y | N | |
| 98 | 289 | | | Ag | 0.00 | N | Y | |
| 99 | 294 | | | Bg | 0.00 | N | Y | |
| 100 | 300 | 291 | 18 | Au | 1.04 | Y | N | S2-C8-C7 bend + C8-S2-C9 bend |
| 101 | 301 | | | Bu | 1.76 | Y | N | |
| 102 | 305 | | | Bg | 0.00 | N | Y | |
| 103 | 306 | | | Ag | 0.00 | N | Y | |
| 104 | 308 | 305 | 6 | Au | 0.35 | Y | N | S2-C8-C7 bend + C8-S2-C9 bend |
| 105 | 316 | | | Bu | 1.75 | Y | N | |
| 106 | 321 | | | Ag | 0.00 | N | Y | |
| 107 | 325 | | | Bg | 0.00 | N | Y | |
| 108 | 326 | 322 | 10 | Ag | 0.00 | N | Y | N2-C4-C5 bend |
| 109 | 333 | | | Au | 8.64 | Y | N | |
| 110 | 341 | | | Bu | 4.37 | Y | N | |
| 111 | 352 | | | Au | 0.08 | Y | N | |
| 112 | 353 | 345 | 20 | Bg | 0.00 | N | Y | C3-C4-C5 bend |
| 113 | 358 | | | Au | 2.63 | Y | N | |
| 114 | 362 | | | Bg | 0.00 | N | Y | |
| 115 | 375 | | | Au | 1.19 | Y | N | |
| 116 | 378 | 368 | 20 | Ag | 0.00 | N | Y | O=C5-C4 bend |
| 117 | 378 | | | Bu | 5.55 | Y | N | |
| 118 | 380 | | | Ag | 0.00 | N | Y | |
| 119 | 381 | | | Bu | 2.96 | Y | N | |
| 120 | 382 | 380 | 4 | Bg | 0.00 | N | Y | Carboxylate rock |
| 121 | 481 | | | Bg | 0.00 | N | Y | |
| 122 | 482 | | | Au | 1.29 | Y | N | |
| 123 | 489 | | | Ag | 0.00 | N | Y | |
| 124 | 489 | 485 | 9 | Bu | 2.37 | Y | N | C2-C3-C4 bend |
| 125 | 493 | | | Ag | 0.00 | N | Y | |
| 126 | 494 | | | Bu | 0.35 | Y | N | |
| 127 | 496 | | | Au | 0.24 | Y | N | |
| 128 | 501 | 496 | 8 | Bg | 0.00 | N | Y | N2-C4-C3 bend |
| 129 | 545 | | | Ag | 0.00 | N | Y | |
| 130 | 547 | | | Bu | 4.15 | Y | N | |
| 131 | 550 | | | Bg | 0.00 | N | Y | |
| 132 | 554 | 549 | 9 | Au | 0.71 | Y | N | NH3 torsion |
| 133 | 559 | | | Bg | 0.00 | N | Y | |
| 134 | 559 | | | Au | 2.20 | Y | N | |
| 135 | 562 | | | Bu | 4.39 | Y | N | |
| 136 | 571 | 563 | 12 | Ag | 0.00 | N | Y | C4-N2 stretch |
| 137 | 603 | | | Au | 1.64 | Y | N | |
| 138 | 606 | | | Bu | 15.81 | Y | N | |
| 139 | 608 | | | Ag | 0.00 | N | Y | |
| 140 | 609 | 607 | 6 | Bg | 0.00 | N | Y | N1-H out-of-plane bend |
| 141 | 632 | | | Bg | 0.00 | N | Y | |
| 142 | 634 | | | Au | 2.26 | Y | N | |
| 143 | 635 | | | Bu | 0.56 | Y | N | |
| 144 | 635 | 634 | 3 | Ag | 0.00 | N | Y | C3 methylene rock |
| 145 | 656 | | | Bu | 1.98 | Y | N | |
| 146 | 657 | | | Ag | 0.00 | N | Y | |
| 147 | 660 | | | Au | 2.16 | Y | N | |
| 148 | 661 | 658 | 5 | Bg | 0.00 | N | Y | C1-S1 + S1-C2 in phase stretch |
| 149 | 688 | | | Bu | 0.40 | Y | N | |
| 150 | 689 | | | Ag | 0.00 | N | Y | |
| 151 | 694 | | | Bg | 0.00 | N | Y | |
| 152 | 694 | 691 | 6 | Au | 1.23 | Y | N | C4-C5 stretch |
| 153 | 701 | | | Au | 0.00 | Y | N | |
| 154 | 701 | | | Ag | 0.00 | N | Y | |
| 155 | 702 | | | Bu | 0.03 | Y | N | |
| 156 | 703 | 702 | 2 | Bg | 0.00 | N | Y | C9-S2 + S2-C8 in phase stretch |
| 157 | 713 | | | Au | 0.08 | Y | N | |
| 158 | 713 | | | Bu | 0.06 | Y | N | |
| 159 | 718 | | | Ag | 0.00 | N | Y | |
| 160 | 718 | 716 | 5 | Bg | 0.00 | N | Y | C1-S1 + S1-C2 out-of-phase stretch and C2 + C3 in-phase rock |
| 161 | 730 | | | Ag | 0.00 | N | Y | |
| 162 | 730 | | | Au | 0.19 | Y | N | |
| 163 | 731 | | | Bg | 0.00 | N | Y | |
| 164 | 732 | 731 | 1 | Bu | 0.29 | Y | N | C7 + C8 out-of-phase methylene rock |
| 165 | 738 | | | Bu | 1.06 | Y | N | |
| 166 | 739 | | | Ag | 0.00 | N | Y | |
| 167 | 739 | | | Au | 0.05 | Y | N | |
| 168 | 739 | 739 | 1 | Bg | 0.00 | N | Y | C9-S2 + S2-C8 out-of-phase stretch |
| 169 | 743 | | | Au | 0.00 | Y | N | |
| 170 | 745 | | | Ag | 0.00 | N | Y | |
| 171 | 745 | | | Bg | 0.00 | N | Y | |
| 172 | 745 | 744 | 2 | Bu | 0.83 | Y | N | N1-H out-of-plane bend |
| 173 | 793 | | | Bu | 0.34 | Y | N | |
| 174 | 793 | | | Ag | 0.00 | N | Y | |
| 175 | 795 | | | Bg | 0.00 | N | Y | |
| 176 | 795 | 794 | 2 | Au | 0.80 | Y | N | C3-C4 stretch |
| 177 | 815 | | | Bu | 2.39 | Y | N | |
| 178 | 815 | | | Ag | 0.00 | N | Y | |
| 179 | 816 | | | Au | 1.78 | Y | N | |
| 180 | 817 | 816 | 2 | Bg | 0.00 | N | Y | Carboxylate O-C10-O in-plane bend |
| 181 | 858 | | | Au | 1.75 | Y | N | |

| | | | | | | | | |
|---|---|---|---|---|---|---|---|---|
| 182 | 859 | | | Bg | 0.00 | N | Y | |
| 183 | 863 | | | Bu | 0.40 | Y | N | |
| 184 | 863 | 861 | 5 | Ag | 0.00 | N | Y | C6-C7 + C7-C8 stretch |
| 185 | 899 | | | Bg | 0.00 | N | Y | |
| 186 | 900 | | | Au | 0.01 | Y | N | |
| 187 | 903 | | | Ag | 0.00 | N | Y | |
| 188 | 904 | 901 | 5 | Bu | 0.12 | Y | N | C6-C7 + C7-C8 stretch |
| 189 | 916 | | | Bu | 1.66 | Y | N | |
| 190 | 917 | | | Ag | 0.00 | N | Y | |
| 191 | 918 | | | Bg | 0.00 | N | Y | |
| 192 | 919 | 917 | 4 | Au | 1.02 | Y | N | C7 + C8 in-phase methylene rock |
| 193 | 927 | | | Ag | 0.00 | N | Y | |
| 194 | 928 | | | Bu | 1.54 | Y | N | |
| 195 | 929 | | | Au | 0.76 | Y | N | |
| 196 | 930 | 929 | 3 | Bg | 0.00 | N | Y | C2-C3 stretch |
| 197 | 937 | | | Bu | 0.80 | Y | N | |
| 198 | 940 | | | Au | 0.46 | Y | N | |
| 199 | 941 | | | Ag | 0.00 | N | Y | |
| 200 | 941 | 940 | 4 | Bg | 0.00 | N | Y | C1 methyl rock |
| 201 | 944 | | | Bg | 0.00 | N | Y | |
| 202 | 946 | | | Bu | 0.06 | Y | N | |
| 203 | 946 | | | Au | 0.00 | Y | N | |
| 204 | 946 | 946 | 3 | Ag | 0.00 | N | Y | C9 methyl rock |
| 205 | 953 | | | Ag | 0.00 | N | Y | |
| 206 | 954 | | | Au | 0.03 | Y | N | |
| 207 | 954 | | | Bu | 0.36 | Y | N | |
| 208 | 954 | 954 | 1 | Bg | 0.00 | N | Y | C9 methyl rock |
| 209 | 960 | | | Ag | 0.00 | N | Y | |
| 210 | 962 | | | Bg | 0.00 | N | Y | |
| 211 | 965 | | | Bu | 1.01 | Y | N | |
| 212 | 965 | 963 | 5 | Au | 0.33 | Y | N | C1 methyl rock |
| 213 | 976 | | | Ag | 0.00 | N | Y | |
| 214 | 977 | | | Bg | 0.00 | N | Y | |
| 215 | 978 | | | Au | 0.00 | Y | N | |
| 216 | 978 | 977 | 3 | Bu | 1.27 | Y | N | C2 + C3 out-of-phase methylene rock |
| 217 | 1004 | | | Bu | 1.97 | Y | N | |
| 218 | 1006 | | | Ag | 0.00 | N | Y | |
| 219 | 1006 | | | Bg | 0.00 | N | Y | |
| 220 | 1007 | 1006 | 3 | Au | 0.01 | Y | N | C6-C10 stretch |
| 221 | 1019 | | | Au | 0.79 | Y | N | |
| 222 | 1020 | | | Ag | 0.00 | N | Y | |
| 223 | 1023 | | | Bg | 0.00 | N | Y | |
| 224 | 1023 | 1021 | 4 | Bu | 0.84 | Y | N | C4-NH3 stretch |
| 225 | 1037 | | | Bg | 0.00 | N | Y | |
| 226 | 1038 | | | Au | 0.02 | Y | N | |
| 227 | 1038 | | | Bu | 0.64 | Y | N | |
| 228 | 1038 | 1038 | 1 | Ag | 0.00 | N | Y | C7-C8 stretch |
| 229 | 1056 | | | Bg | 0.00 | N | Y | |
| 230 | 1056 | | | Au | 0.23 | Y | N | |
| 231 | 1056 | | | Ag | 0.00 | N | Y | |
| 232 | 1056 | 1056 | 0 | Bu | 0.81 | Y | N | C2-C3 stretch |
| 233 | 1068 | | | Bu | 3.39 | Y | N | |
| 234 | 1070 | | | Bg | 0.00 | N | Y | |
| 235 | 1070 | | | Ag | 0.00 | N | Y | |
| 236 | 1072 | 1070 | 4 | Au | 1.43 | Y | N | C7 + C8 out-of-phase methylene twist |
| 237 | 1117 | | | Au | 0.03 | Y | N | |
| 238 | 1119 | | | Ag | 0.00 | N | Y | |
| 239 | 1120 | | | Bg | 0.00 | N | Y | |
| 240 | 1121 | 1119 | 4 | Bu | 2.45 | Y | N | NH3 rock |
| 241 | 1125 | | | Au | 1.93 | Y | N | |
| 242 | 1125 | | | Ag | 0.00 | N | Y | |
| 243 | 1126 | | | Bg | 0.00 | N | Y | |
| 244 | 1126 | 1125 | 1 | Bu | 0.62 | Y | N | C2 methylene twist |
| 245 | 1147 | | | Bu | 4.52 | Y | N | |
| 246 | 1148 | | | Bg | 0.00 | N | Y | |
| 247 | 1150 | | | Au | 0.39 | Y | N | |
| 248 | 1151 | 1149 | 4 | Ag | 0.00 | N | Y | C2 + C3 out-of-phase methylene twist |
| 249 | 1153 | | | Bu | 1.79 | Y | N | |
| 250 | 1154 | | | Bg | 0.00 | N | Y | |
| 251 | 1157 | | | Au | 0.01 | Y | N | |
| 252 | 1158 | 1156 | 4 | Ag | 0.00 | N | Y | NH3 rock |
| 253 | 1176 | | | Ag | 0.00 | N | Y | |
| 254 | 1177 | | | Bu | 1.11 | Y | N | |
| 255 | 1177 | | | Au | 4.11 | Y | N | |
| 256 | 1178 | 1177 | 2 | Bg | 0.00 | N | Y | C6-H bend along C6-C7 |
| 257 | 1198 | | | Ag | 0.00 | N | Y | |
| 258 | 1198 | | | Au | 0.44 | Y | N | |
| 259 | 1198 | | | Bu | 6.13 | Y | N | |
| 260 | 1199 | 1199 | 1 | Bg | 0.00 | N | Y | C4-C5 stretch |
| 261 | 1230 | | | Bg | 0.00 | N | Y | |
| 262 | 1231 | | | Au | 0.06 | Y | N | |
| 263 | 1231 | | | Bu | 1.77 | Y | N | |
| 264 | 1232 | 1231 | 2 | Ag | 0.00 | N | Y | C8 methylene wag |
| 265 | 1234 | | | Ag | 0.00 | N | Y | |
| 266 | 1234 | | | Au | 4.26 | Y | N | |
| 267 | 1235 | | | Bu | 0.54 | Y | N | |
| 268 | 1235 | 1234 | 1 | Bg | 0.00 | N | Y | C2 methylene wag |
| 269 | 1262 | | | Bu | 0.46 | Y | N | |
| 270 | 1262 | | | Bg | 0.00 | N | Y | |
| 271 | 1262 | | | Au | 0.13 | Y | N | |
| 272 | 1263 | 1262 | 1 | Ag | 0.00 | N | Y | C2 + C3 in-phase methylene twist |
| 273 | 1265 | | | Ag | 0.00 | N | Y | |
| 274 | 1266 | | | Bu | 1.31 | Y | N | |

| | | | | | | | | |
|---|---|---|---|---|---|---|---|---|
| 275 | 1266 | | | Bg | 0.00 | N | Y | |
| 276 | 1267 | 1266 | 2 | Au | 0.08 | Y | N | C7 + C8 in-phase methylene twist |
| 277 | 1289 | | | Bg | 0.00 | N | Y | |
| 278 | 1291 | | | Au | 0.46 | Y | N | |
| 279 | 1292 | | | Ag | 0.00 | N | Y | |
| 280 | 1294 | 1291 | 5 | Bu | 0.45 | Y | N | C6-H bend along C6-N1 |
| 281 | 1299 | | | Au | 0.02 | Y | N | |
| 282 | 1300 | | | Ag | 0.00 | N | Y | |
| 283 | 1302 | | | Bu | 0.47 | Y | N | |
| 284 | 1303 | 1301 | 5 | Bg | 0.00 | N | Y | C9-H sym methyl bend |
| 285 | 1312 | | | Au | 0.27 | Y | N | |
| 286 | 1314 | | | Bu | 0.09 | Y | N | |
| 287 | 1316 | | | Bg | 0.00 | N | Y | |
| 288 | 1317 | 1315 | 5 | Ag | 0.00 | N | Y | C7 methylene wag |
| 289 | 1319 | | | Bu | 1.03 | Y | N | |
| 290 | 1319 | | | Au | 0.23 | Y | N | |
| 291 | 1320 | | | Bg | 0.00 | N | Y | |
| 292 | 1320 | 1319 | 2 | Ag | 0.00 | N | Y | C1-H sym methyl bend |
| 293 | 1321 | | | Bu | 1.46 | Y | N | |
| 294 | 1322 | | | Au | 0.77 | Y | N | |
| 295 | 1322 | | | Ag | 0.00 | N | Y | |
| 296 | 1322 | 1322 | 1 | Bg | 0.00 | N | Y | C3 methylene wag |
| 297 | 1335 | | | Au | 2.48 | Y | N | |
| 298 | 1335 | | | Ag | 0.00 | N | Y | |
| 299 | 1337 | | | Bg | 0.00 | N | Y | |
| 300 | 1338 | 1336 | 3 | Bu | 0.64 | Y | N | C4-H bend along C4-C3 |
| 301 | 1353 | | | Bu | 24.63 | Y | N | |
| 302 | 1353 | | | Ag | 0.00 | N | Y | |
| 303 | 1365 | | | Au | 3.38 | Y | N | |
| 304 | 1366 | 1359 | 13 | Bg | 0.00 | N | Y | C4-H bend along C4-N2 |
| 305 | 1374 | | | Bg | 0.00 | N | Y | |
| 306 | 1375 | | | Au | 12.77 | Y | N | |
| 307 | 1377 | | | Ag | 0.00 | N | Y | |
| 308 | 1379 | 1376 | 5 | Bu | 8.03 | Y | N | C3 methylene wag |
| 309 | 1405 | | | Ag | 0.00 | N | Y | |
| 310 | 1408 | | | Bg | 0.00 | N | Y | |
| 311 | 1408 | | | Au | 0.62 | Y | N | |
| 312 | 1409 | 1407 | 4 | Bu | 0.83 | Y | N | C9-H asym methyl bend |
| 313 | 1411 | | | Bu | 0.33 | Y | N | |
| 314 | 1412 | | | Bg | 0.00 | N | Y | |
| 315 | 1412 | | | Ag | 0.00 | N | Y | |
| 316 | 1413 | 1412 | 2 | Au | 0.43 | Y | N | C9-H asym methyl bend |
| 317 | 1417 | | | Ag | 0.00 | N | Y | |
| 318 | 1419 | | | Au | 1.16 | Y | N | |
| 319 | 1423 | | | Bg | 0.00 | N | Y | |
| 320 | 1424 | 1421 | 7 | Bu | 0.19 | Y | N | C1-H asym methyl bend |
| 321 | 1425 | | | Au | 1.00 | Y | N | |
| 322 | 1425 | | | Bg | 0.00 | N | Y | |
| 323 | 1426 | | | Ag | 0.00 | N | Y | |
| 324 | 1427 | 1426 | 2 | Bu | 0.35 | Y | N | C3 + C7 in-phase methylene scissors |
| 325 | 1429 | | | Bg | 0.00 | N | Y | |
| 326 | 1430 | | | Au | 0.73 | Y | N | |
| 327 | 1430 | | | Bu | 1.20 | Y | N | |
| 328 | 1431 | 1430 | 2 | Bg | 0.00 | N | Y | C8 methylene scissors |
| 329 | 1431 | | | Ag | 0.00 | N | Y | |
| 330 | 1432 | | | Bu | 2.79 | Y | N | |
| 331 | 1433 | | | Ag | 0.00 | N | Y | |
| 332 | 1435 | 1433 | 4 | Au | 0.20 | Y | N | C2 + C8 in-phase methylene scissors |
| 333 | 1437 | | | Bu | 0.48 | Y | N | |
| 334 | 1439 | | | Au | 0.94 | Y | N | |
| 335 | 1440 | | | Bg | 0.00 | N | Y | |
| 336 | 1442 | 1439 | 5 | Bu | 0.88 | Y | N | C1-H asym methyl bend |
| 337 | 1442 | | | Ag | 0.00 | N | Y | |
| 338 | 1442 | | | Bg | 0.00 | N | Y | |
| 339 | 1443 | | | Au | 2.25 | Y | N | |
| 340 | 1445 | 1443 | 4 | Ag | 0.00 | N | Y | C3 + C7 out-of-phase methylene scissors |
| 341 | 1481 | | | Bu | 36.31 | Y | N | |
| 342 | 1485 | | | Ag | 0.00 | N | Y | |
| 343 | 1489 | | | Bg | 0.00 | N | Y | |
| 344 | 1490 | 1486 | 9 | Au | 0.10 | Y | N | N1-H in-plane bend |
| 345 | 1510 | | | Ag | 0.00 | N | Y | |
| 346 | 1514 | | | Bg | 0.00 | N | Y | |
| 347 | 1522 | | | Au | 38.48 | Y | N | |
| 348 | 1533 | 1520 | 23 | Bu | 23.85 | Y | N | NH3 sym bend |
| 349 | 1557 | | | Bu | 27.65 | Y | N | |
| 350 | 1557 | | | Au | 62.99 | Y | N | |
| 351 | 1558 | | | Bu | 2.14 | Y | N | |
| 352 | 1561 | 1558 | 4 | Ag | 0.00 | N | Y | Asym O-C-O stretch |
| 353 | 1562 | | | Bg | 0.00 | N | Y | |
| 354 | 1572 | | | Bg | 0.00 | N | Y | |
| 355 | 1582 | | | Ag | 0.00 | N | Y | |
| 356 | 1582 | 1574 | 21 | Au | 2.38 | Y | N | NH3 asym bend |
| 357 | 1605 | | | Ag | 0.00 | N | Y | |
| 358 | 1612 | | | Au | 17.40 | Y | N | |
| 359 | 1617 | | | Bg | 0.00 | N | Y | |
| 360 | 1622 | 1614 | 17 | Bu | 12.41 | Y | N | C5=O stretch |
| 361 | 1646 | | | Ag | 0.00 | N | Y | |
| 362 | 1646 | | | Bg | 0.00 | N | Y | |
| 363 | 1650 | | | Bu | 5.49 | Y | N | |
| 364 | 1651 | 1648 | 5 | Au | 11.78 | Y | N | NH3 asym bend |
| 365 | 2721 | | | Ag | 0.00 | N | Y | |
| 366 | 2724 | | | Bg | 0.00 | N | Y | |
| 367 | 2725 | | | Bu | 100.86 | Y | N | |

| | | | | | | | | |
|---|---|---|---|---|---|---|---|---|
| 368 | 2738 | 2727 | 17 | Au | 113.32 | Y | N | NH3 asymmetric stretch |
| 369 | 2883 | | | Ag | 0.00 | N | Y | |
| 370 | 2883 | | | Bu | 76.45 | Y | N | |
| 371 | 2886 | | | Bg | 0.00 | N | Y | |
| 372 | 2894 | 2886 | 11 | Au | 60.97 | Y | N | NH3 asymmetric stretch |
| 373 | 2931 | | | Au | 0.03 | Y | N | |
| 374 | 2932 | | | Bu | 3.60 | Y | N | |
| 375 | 2933 | | | Bg | 0.00 | N | Y | |
| 376 | 2933 | 2932 | 2 | Ag | 0.00 | N | Y | C1 sym methyl stretch |
| 377 | 2938 | | | Bu | 0.08 | Y | N | |
| 378 | 2938 | | | Bg | 0.00 | N | Y | |
| 379 | 2938 | | | Au | 2.06 | Y | N | |
| 380 | 2938 | 2938 | 0 | Ag | 0.00 | N | Y | C3 sym methylene stretch |
| 381 | 2942 | | | Bu | 2.31 | Y | N | |
| 382 | 2942 | | | Bg | 0.00 | N | Y | |
| 383 | 2944 | | | Ag | 0.00 | N | Y | |
| 384 | 2944 | 2943 | 2 | Au | 0.77 | Y | N | C9 sym methyl stretch |
| 385 | 2949 | | | Au | 3.48 | Y | N | |
| 386 | 2949 | | | Bu | 1.28 | Y | N | |
| 387 | 2949 | | | Bg | 0.00 | N | Y | |
| 388 | 2949 | 2949 | 0 | Ag | 0.00 | N | Y | C2 sym methylene stretch |
| 389 | 2956 | | | Bu | 0.55 | Y | N | |
| 390 | 2956 | | | Ag | 0.00 | N | Y | |
| 391 | 2957 | | | Bg | 0.00 | N | Y | |
| 392 | 2957 | 2956 | 0 | Au | 0.38 | Y | N | C7 asym methylene stretch |
| 393 | 2963 | | | Ag | 0.00 | N | Y | |
| 394 | 2963 | | | Au | 0.12 | Y | N | |
| 395 | 2963 | | | Bg | 0.00 | N | Y | |
| 396 | 2963 | 2963 | 0 | Bu | 0.65 | Y | N | C3 asym methylene stretch |
| 397 | 2994 | | | Au | 0.02 | Y | N | |
| 398 | 2994 | | | Bg | 0.00 | N | Y | |
| 399 | 2994 | | | Ag | 0.00 | N | Y | |
| 400 | 2994 | 2994 | 0 | Bu | 0.46 | Y | N | C8 asym methylene stretch |
| 401 | 2995 | | | Ag | 0.00 | N | Y | |
| 402 | 2995 | | | Bu | 0.72 | Y | N | |
| 403 | 2995 | | | Au | 0.31 | Y | N | |
| 404 | 2995 | 2995 | 0 | Bg | 0.00 | N | Y | C4-H stretch |
| 405 | 3000 | | | Ag | 0.00 | N | Y | |
| 406 | 3000 | | | Au | 1.68 | Y | N | |
| 407 | 3000 | | | Bu | 0.99 | Y | N | |
| 408 | 3000 | 3000 | 0 | Bg | 0.00 | N | Y | C2 asym methylene stretch |
| 409 | 3017 | | | Ag | 0.00 | N | Y | |
| 410 | 3017 | | | Bg | 0.00 | N | Y | |
| 411 | 3018 | | | Bu | 0.60 | Y | N | |
| 412 | 3018 | 3017 | 1 | Au | 0.23 | Y | N | C1 asym methyl stretch |
| 413 | 3020 | | | Ag | 0.00 | N | Y | |
| 414 | 3020 | | | Bg | 0.00 | N | Y | |
| 415 | 3020 | | | Au | 0.04 | Y | N | |
| 416 | 3020 | 3020 | 0 | Bu | 0.63 | Y | N | C3 asym methylene stretch |
| 417 | 3027 | | | Au | 0.05 | Y | N | |
| 418 | 3027 | | | Ag | 0.00 | N | Y | |
| 419 | 3029 | | | Bg | 0.00 | N | Y | |
| 420 | 3029 | 3028 | 2 | Bu | 0.58 | Y | N | C9 asym methyl stretch |
| 421 | 3042 | | | Bu | 0.15 | Y | N | |
| 422 | 3042 | | | Au | 0.04 | Y | N | |
| 423 | 3042 | | | Ag | 0.00 | N | Y | |
| 424 | 3042 | 3042 | 1 | Bg | 0.00 | N | Y | C1 asym methyl stretch |
| 425 | 3051 | | | Bu | 0.28 | Y | N | |
| 426 | 3051 | | | Au | 1.22 | Y | N | |
| 427 | 3052 | | | Ag | 0.00 | N | Y | |
| 428 | 3052 | 3052 | 0 | Bg | 0.00 | N | Y | C6-H stretch |
| 429 | 3052 | | | Au | 0.11 | Y | N | |
| 430 | 3052 | | | Ag | 0.00 | N | Y | |
| 431 | 3053 | | | Bg | 0.00 | N | Y | |
| 432 | 3053 | 3052 | 1 | Bu | 0.29 | Y | N | C9 asym methyl stretch |
| 433 | 3060 | | | Bu | 0.22 | Y | N | |
| 434 | 3060 | | | Au | 0.07 | Y | N | |
| 435 | 3060 | | | Bg | 0.00 | N | Y | |
| 436 | 3060 | 3060 | 0 | Ag | 0.00 | N | Y | C7 asym methylene stretch |
| 437 | 3128 | | | Ag | 0.00 | N | Y | |
| 438 | 3129 | | | Au | 20.64 | Y | N | |
| 439 | 3139 | | | Bg | 0.00 | N | Y | |
| 440 | 3145 | 3135 | 17 | Bu | 83.98 | Y | N | NH3 asym stretch |
| 441 | 3390 | | | Bu | 2.39 | Y | N | |
| 442 | 3390 | | | Bg | 0.00 | N | Y | |
| 443 | 3390 | | | Ag | 0.00 | N | Y | |
| 444 | 3391 | 3390 | 0 | Au | 17.34 | Y | N | N1-H stretch |
